# Comparison of lifestyle, cognitive function, mental health, and quality of life between hospitalized older adults with COVID-19 and non-COVID-19 in South Korea: a cross-sectional study

**DOI:** 10.1186/s12877-023-04646-y

**Published:** 2024-04-02

**Authors:** Jae Hyu Jung, Ji-Hyuk Park, Kang-Hyun Park

**Affiliations:** 1 Department of Occupational Therapy, Gyeonggi Provincial Medical Center, Anseong, Korea; 2 Department of Occupational Therapy, College of Software and Digital Healthcare Convergence, Yonsei University, Wonju, Korea; 3 Department of Occupational Therapy, Baekseok University, Cheonan, Korea

**Keywords:** Memory, Cognitive impairment, Mental health, Depression, Insomnia, Quality of life

## Abstract

**Background:**

The coronavirus disease (COVID-19) pandemic has greatly impacted older adults, resulting in many deaths. The impact of lifestyle and mental health on vulnerable groups, such as older adults, can be large and long lasting. Therefore, this study aimed to investigate the effects of COVID-19 confirmation on cognition, lifestyle, mental health, and quality of life in adults aged 55 years.

**Methods:**

The sample consisted of 111 people in the COVID group and 189 people in the non-COVID group aged over 55 years in South Korea. An online survey was conducted between January and May 2022. Participants responded to the following assessment tools: Yonsei Lifestyle Profile, Prospective and Retrospective Memory (PRMQ), Subjective Memory Complaints Questionnaire (SMCQ), Visual Analogue Scale, Patient Health Questionnaire-9 (PHQ-9), Insomnia Severity Index (ISI), Fear of COVID-19 Scale (FCV-19 S), and the World Health Organization Quality of Life Scale abbreviated version (WHOQOL-BREF). Differences in lifestyle, cognition, depression, anxiety, and quality of life were compared between the two groups.

**Results:**

There were significant differences in physical activity, diet, the total score of the PRMQ, PM (a sub-score of the PRMQ), PHQ-9, Korean version of the ISI (ISI-K), and WHOQOL-BREF scores between the COVID and non-COVID groups. However, there were no significant differences in activity participation, Self-Rating Anxiety Scale (SAS), or FCV-19 S between groups.

**Conclusions:**

The study confirms that COVID-19 negatively affects memory, physical activity, diet, quality of life, depression, and insomnia in the older adults. Therefore, this study implicated that prevention and intervention strategies required improving the memory, lifestyle, and mental health of older adults with COVID-19.

**Trial registration:**

The study was conducted in accordance with the Declaration of Helsinki, and approved by the Institutional Review Board of Yonsei university in Korea (Registration number: 1041849-202112-SB-226-03, Date of registration: 01042022).

**Supplementary Information:**

The online version contains supplementary material available at 10.1186/s12877-023-04646-y.

## Background

COVID-19 was declared a pandemic by the World Health Organization (WHO) on March 11, 2020 [1]. The WHO Health Organization has identified COVID-19 as a public health emergency. This is because COVID-19 is characterized by rapid contamination and mortality and has a wide range of clinical manifestations in patients with COVID-19. COVID-19 can affect the lower respiratory tract in humans and can cause diseases ranging from simple colds to severe infections with up to 50% lethality [2].

According to multinational data from clinical studies, retrospective mining of electronic health records, case reports, and various surveys, COVID-19 has the capacity to damage the brain [3]. In several studies, patients showed neurological manifestations, with central involvement being more common, including dizziness, headache, altered level of consciousness, stroke, ataxia, and epilepsy [4]. Regarding more specific cognitive impairment, patients with COVID-19 demonstrate attention and executive function impairment in the acute stage [5] and other neuropsychological impairments, such as memory and verbal fluency [6]. In systematic reviews, subjective cognitive dysfunction was found to be 23.8% [7].

Prospective memory refers to the ability to plan future events and execute them successfully [8]. For example, remembering and meeting family appointments and remembering the shopping list and making purchases are prospective memories. On the other hand, retrospective memory means remembering past events. For example, memories of riding a bicycle with father as a child or talking about stories at mealtime are retrospective memories [9]. Previous research has demonstrated that RM is a prerequisite for PM, but not vice versa. When RM is damaged, PM tends to be low, but RM is preserved when PM is damaged [9]. Prospective memory affects a wide range of daily activities in older adults. Therefore, prospective memory is an important factor in maintaining independence and autonomy [10]. We need to find out whether COVID-19 affects prospective memory.

Social isolation with COVID-19 has had a negative impact on the lifestyle, mental health, and quality of life of many older adults [11–13]. Tosato et al., (2022) reported that 56.3% older adult has changed in lifestyle after the COVID-19 pandemic, and their quality of life worsened compared to before COVID-19. Visits to relative and physical activity among lifestyle showed the most changes [12]. Additionally, studies in Italy, Spain, France, and the United States showed that people felt more depressed and lonelier after the pandemic [14, 15]. In a Canadian study, the odds of depressive symptoms doubled during the pandemic compared to the pre-pandemic period [16]. Most studies have been conducted on changes in lifestyle, mental health, and quality of life of older adults in covid-19 pandemic. There is a lack of research on changes in lifestyle, mental health, and quality of life due to confirmed COVID-19.

Although there is much evidence regarding the correlation between COVID-19 and cognitive decline, evaluations and interventions related to cognitive impairment in patients with COVID-19 are currently lacking. Moreover, older adults aged > 55 years tend to be linked to more severe forms of COVID-19 and, therefore, potentially more severe cognitive impairment [17]. Previous studies have reported that COVID-19 negatively affects cognition, lifestyle, mental health, and quality of life [4, 18, 19]. However, these studies only partially interpreted the relationship between COVID-19 and these variables.

Therefore, the purpose of the present study was to investigate the effects of COVID-19 confirmation on cognition, lifestyle, mental health, and quality of life in adults aged 55 years or older by comparing older adults with and without COVID-19 infection.

We hypothesized that:

There will be differences in the lifestyles of hospitalized older adults with COVID-19 and non-COVID-19.

The memory, mental health, and quality of life of hospitalized older adults with COVID-19 will be worse than that of non-COVID-19.

## Methods

### Participants and data collection

We conducted a prospective study of patients hospitalized for COVID-19 at local hospital, South Korea, from January to May 2022. The inclusion criteria were positive SARS-CoV-2 polymerase chain reaction (PCR) results by nasopharyngeal or tracheal testing, hospitalization for COVID-19, and age 55 years. The exclusion criteria were a lack of Korean language proficiency and dementia. Older adults with COVID-19 responded to a Google questionnaire via mobile phone. Older adults without COVID-19 were recruited using an online survey and they also answered the questionnaire via mobile phone. The research team provided information about the purpose of the study, and informed consent was obtained from all participants before participating. This study was approved by the Institutional Review Board of Yonsei university in Korea (1041849-202112-SB-226-03).

### Measurements

#### Lifestyle indicator

The Yonsei Lifestyle Profile-BREF Lifestyle of older adults with and without COVID-19 was measured using the Yonsei Lifestyle Profile-BREF (YLP-BREF) questionnaire [20]. The YLP-BREF comprises 21 items that measure multifaceted lifestyle factors, including physical activity, activity participation, and nutrition. The YLP-BREF showed high internal reliability with a Cronbach’s alpha of 0.83. In this study, Cronbach’s alpha was 0.84. The questionnaire is provided in Supplementary File S1.

#### Cognitive function indicators

The Prospective and Retrospective Memory Questionnaire (PRMQ) was used to measure self-reported memory problems. The PRMQ consists of 16 items that assess memory failure in everyday life [21]. Half of the PRMQ items inquire about PM (Prospective Memory), and the other half about RM (Retrospective Memory). The reliability of the Korean version of the PRMQ has been found to be acceptable. Each domain consists of eight items rated on a five-point Likert scale, ranging from 5 (very often) to 1 (never). The total scores ranged from 16 to 80. Higher scores indicated more memory complaints. In this study, Cronbach’s alpha was 0.92, indicating good internal consistency.

The Subjective Memory Complaints Questionnaire (SMCQ) was developed to evaluate subjective memory complains [22]. The SMCQ has 14 items, each of which is answered with either ‘yes’ or ‘no’. Higher SMCQ scores indicated more severe subjective memory complaints. In this study, Cronbach’s alpha was 0.79.

#### Mental health indicator

The Korean version of the insomnia severity index (ISI-K) is a self-report questionnaire that evaluates the nature, severity, and impact of insomnia [23]. A 5-point Likert scale ranging from 0 to 28 was used to rate each item. The higher the total score, the greater the severity of insomnia. In this study, Cronbach’s alpha was 0.87.

The Fear of COVID-19 Scale (FCV-19 S) was used to evaluate fear of COVID-19 among participants. The FCV-19 S consists of seven items evaluated on a 5-point Likert scale [24]. The Korean version of the FCV-19 S has acceptable psychometric properties [25]. In this study, Cronbach’s alpha was 0.89.

To measure self-reported depression and anxiety, the Visual Analog Scale (VAS) Depression and Visual Analog Scale (VAS) Anxiety were used. The participants were required to answer their level of depression and anxiety by selecting any point among the continuum set of possible values ranging from 0 (not all depressed or anxious) to 100 (most depressed or anxious I can imagine). The VAS has been demonstrated to perform well in the assessment of a variety of health outcomes, such as stress [26], depressive symptoms [27], and anxiety [28].

The Patient Health Questionnaire-9 (PHQ-9) is used to screen for depression in primary care and medical setting [29]. The PHQ-9 was developed as a self-administered diagnostic screening assessment tool used by healthcare professionals to evaluate and monitor depression severity [30]. The PHQ-9 consists of nine items, and the standard cutoff score for screening to identify possible major depression is 10 or above. In this study, Cronbach’s alpha was 0.93.

The Zung Self-Rating Anxiety Scale (SAS) is a self-report questionnaire consisting of 20 items regarding a variety of anxiety symptoms, including psychological and somatic symptoms [31]. Responses were given on a 4-point Likert scale ranging from 1 to 4. SAS has demonstrated satisfactory psychometric properties [31]. Cronbach’s alpha for the SAS was 0.79.

#### Quality of life indicator

To measure the quality of life among participants, the World Health Organization Quality of Life Scale abbreviated version (WHOQOL-BREF) was used. The measure consists of 26 items in four major domains: physical, psychological, social, and environmental factors [32]. The items were rated on a five-point Likert scale, and the raw domain scores were converted to a scale ranging from 0 to 100, with higher scores indicating a higher quality of life [33]. In this study, Cronbach’s alpha was 0.94.

### Data analysis

Descriptive statistics were used to analyze the participants’ demographic characteristics. Chi-squared test was used to analysis demographic characteristics in sex, education, work, and underlying disease and independent t-test was used to analysis education between two groups. Paired t-test was used to compare lifestyle between before COVID pandemic and after COVID pandemic within groups. Independent t-test was used to compare lifestyle, cognitive function, mental health, and quality of life between groups. The confidence interval was set at 95%. The *p*-value was two-sided, and statistical significance was set at *p* <.05. All the statistical analyses were performed using SAS version 9.4 (SAS Institute Inc., Cary, NC, USA).

## Results

### Characteristics of the study population

Table 1 presents the general characteristics of the study population. The average age of COVID-19 patients was 68.63, and 35.14% were male, compared to 61.41 years and 48.68% male among non-COVID subjects. Regarding education, COVID-19 patients had the highest level of education under middle school (68.47%), and non-COVID subjects had the highest level of education (68.25%). In both groups, more than 55% of the participants were employed. COVID patients had underlying diseases such as hypertension (44.14%), hyperlipidemia (30.63%), diabetes (24.32%), cardiovascular disease (18.02%), and cerebrovascular disease (7.21%). In the non-COVID group, hypertension (35.45%) was the most common, diabetes and hyperlipidemia were the same at 10%, cardiovascular disease was 2.12%, and no subjects had cerebrovascular disease. There were significant differences in sex (*p* =.022^*^), education (*p* <.0001^*^*), work (*p* <.0001^*^*), diabetes (*p* =.002^*^), hyperlipidemia (*p* <.0001^*^*), cerebrovascular disease (*p* =.0003^*^*), and cardiovascular disease (*p* <.0001^*^*) between the two groups.

**Table 1:** General characteristics

	Classification	COVID Group (*N* = 111)	non-COVID Group (*N* = 189)	p
Sex, n (%)	Male	39(35.14)	92(48.68)	0.022^*^
	Female	72(64.86)	97(51.32)	
Age (year), M (SD)		68.63(7.87)	61.41(5.80)	< 0.0001^**^
Education (year), n (%)	Under middle school	76(68.47)	6(3.18)	< 0.0001^**^
	High school	22(19.82)	54(28.57)	
	Over college	13(11.71)	129(68.25)	
Work, n (%)	Yes	62(55.86)	108(57.14)	< 0.0001^**^
	No	38(34.23)	97(42.86)	
	No response	11(9.91)	0(00.00)	
Underlying disease, n (%)	Hypertension	49(44.14)	67(35.45)	0.135
	Diabetes	27(24.32)	20(10.58)	0.002^*^
	Hyperlipidemia	34(30.63)	20(10.58)	< 0.0001^**^
	Cerebrovascular disease	8(7.21)	0(00.00)	^+^0.0003^**^
	Cardiovascular disease	20(18.02)	4(2.12)	< 0.001^**^

COVID Group = Older adults infected with COVID-19; UNCOVID Group = Older adults not infected with COVID-19; M = Mean; SD = Standard deviation

Independent t-tests, chi-squared tests, and two-tailed Fisher’s exact tests were performed

^+^ = Fisher’s extract

*p <.05, **p <.001

### Lifestyle of covid and non-covid groups

When comparing the lifestyles of the COVID group and non-COVID group, significant differences were observed in physical activity (*p* <.0001^*^*) and diet (*p* <.0001^*^*). However, there was no significant difference in activity participation (*p* =.456) between the two groups. Within the group, physical activity (*p* <.0001^*^*), activity participation (*p* <.0001^*^*), and diet (*p* <.0001^*^*) were significantly decreased in the COVID group when compared before and after COVID confirmation. In the non-COVID group, physical activity (*p* <.0001^*^*) and activity participation (*p* <.0001^*^*) significantly decreased, but there was no significant change in diet (*p* =.303) when comparing before and after the COVID pandemic (Table 2).

**Table 2:** Comparisons of changes in lifestyle within and between groups

Variables		COVID Group				non-COVID Group				CI	p
		M (SD)	Diff	CI	p	M (SD)	Diff	CI	p		
**YLP**											
Physical Activity	Pre	8.94(2.12)	3.77(2.10)	[3.77, 4.17]	< 0.0001^**^	11.23(3.44)	1.41(3.17)	(0.95, 1.86)	< 0.001^**^	[-3.03, -1.70]	< 0.0001^**^
	Post	5.16(0.60)				9.83(3.40)					
Activity Participation	Pre	6.21(2.17)	1.89(2.06)	[1.50, 2.28]	< 0.0001^**^	8.48(2.34)	1.71(2.04)	(1.42, 2.00)	< 0.001^**^	[-0.66, 0.30]	0.465
	Post	4.32(0.59)				6.77(1.90)					
Diet	Pre	34.02(4.46)	4.74(5.03)	[3.79, 5.68]	< 0.0001^**^	33.35(7.77)	0.34(4.58)	(-0.31, 1.00)	0.30	[-5.51, -3.28]	< 0.0001^**^
	Post	29.28(3.34)				33.01(7.72)					

YLP = The Yonsei Lifestyle Profile; COVID Group = Old adult who infected coronavirus disease 2019; UNCOVID Group = Old adult who non-infected coronavirus disease 2019; Diff = Difference. Diff = pre – post; CI = Confidence interval

*p <.05, **p <.001

### Cognition, mental health, and quality of life of covid and non-covid groups

When comparing the cognition questionnaire between the two groups, there was a significant difference in the total scores of the PRMQ (*p* =.024^*^) and PM (*p* = < 0.0001^*^*), a sub-item of the PRMQ. However, there was no significant difference between RM (*p* =.296) in the PRMQ and the total SMCQ score (*p* =.296). In addition, there were significant differences in VAS scores for depression (*p* =.070^*^), PHQ-9 (*p* =.047^*^), ISI-K (*p* =.002^*^), and WHOQOL-BREF (*p* =.004^*^) between the groups, and no significant differences in VAS scores for anxiety (*p* =.754), SAS (*p* =.351), and FCV-19 S (*p* =.943) between the groups (Table 3).

**Table 3:** Comparisons of mental health, quality of life, cognition between groups

Variables	COVID Group	non-COVID Group	CI	p
	M (SD)	M (SD)		
**Cognition**				
PRMQ	32.60(9.49)	30.17(8.68)	[-4.55, -0.32]	0.024^*^
PM	18.48(5.65)	15.47(4.35)	[-4.15, -1.86]	< 0.0001^**^
RM	14.13(4.49)	14.70(4.61)	[-0.50, 1.65]	0.296
SMCQ	2.97(2.65)	2.64(2.55)	[-0.3, 0.29]	0.296
**Mental health**				
VAS_Depression	4.87(2.13)	4.38(2.39)	[-1.04, 0.04]	0.070
PHQ-9	5.11(4.00)	4.14(4.10)	[-1.93, -0.01]	0.047^*^
VAS_Anxiety	5.30(2.62)	5.21(2.03)	[-0.62, 0.44]	0.754
SAS	37.05(7.36)	36.23(7.34)	[-2.55, 0.91]	0.351
ISI-K	10.70(5.20)	8.58(6.25)	[-3.51, -0.74]	0.002^*^
FCV-19 S	17.43(5.36)	17.48(5.94)	[-1.30, 1.40]	0.943
**Quality of Life**				
WHOQOL-BREF	83.91(14.13)	84.0(14.00)	[-8.48, -1.67]	0.004^*^

COVID Group = Old adult who infected coronavirus disease 2019; UNCOVID Group = Old adult who non-infected coronavirus disease 2019; M = mean; SD = standard deviation; CI = Confidence interval; PRMQ = Korean Version of Prospective and Retrospective Memory Questionnaire; PM = Prospective memory; RM = Retrospective memory; SMCQ = Subjective Memory Complaints Questionnaire; VAS_Depression = Visual analogue scale of depression; PHQ-9 = patient health questionnaire-9; VAS_Anxiety = Visual analogue scale of anxiety; SAS = Zung self-rating anxiety scale; ISI-K = Korean version of the insomnia severity index; FCV-19 S = fear of COVID-19 scale; WHOQOL-BREF = World Health Organization quality of life assessment instrument-BREF

*p <.05, **p <.001

## Discussion

The COVID-19 pandemic has severely impacted the lifestyle, cognitive function, mental health, and quality of life of older adults. There were significant effects on COVID-19 of physical activity and diet on lifestyle, depression, and insomnia in terms of mental health and quality of life. In addition, only subjective discomfort with prospective memory was reported to differ significantly between the groups.

There were significant differences in physical activity and diet between the two groups, but no significant differences were observed in activity participation between the two groups. In many countries, social distancing has been ongoing for a long time during the COVID pandemic [34]. Due to social distancing, schools were closed, teleworking was implemented, and mobility was limited in public spaces [35]. Therefore, there were many restrictions on the activities of those who were not infected with COVID-19. The COVID group scored 1.89 and the non-COVID group scored 1.69 in the score of activity participation, indicating that both groups had very low scores. Therefore, there was no significant difference between the two groups.

Physical activity and diet have fewer spatial restrictions than active participation. Therefore, the physical activity and diet scores were relatively high in the non-COVID group. On the other hand, in the case of the COVID group, since they were hospitalized, there were significant spatial restrictions on physical activity, and it was difficult for them to choose a diet by themselves because meals were provided at the hospital. Therefore, physical activity and diet differed significantly between the two groups. Because reduced activity and mobility in older adults during the lockdown can have a negative impact on frailty and wellbeing [34], relevant interventions are needed.

The total scores of the PRMQ and PM and the sub-score of the PRMQ were significantly lower in the COVID group, but the RM of the PRMQ and the total score of the SMCQ were not significantly different between the two groups. Prospective memory includes retrospective memory and several cognitive processes [36]. In addition, cognitive function declines earlier in prospective memory than in retrospective memory, and older adults complain more about prospective memory than about retrospective memory [37, 38]. Systematic reviews also did not confirm any changes in long-term memory by COVID-19, but COVID-19 patients showed lower performance in verbal short-term memory tasks [39]. Self-reported cognitive impairment is associated with decline in mental health, such as anxiety, depression, and PTSD [40]. Stress can contribute to cognitive impairment [41] and depression is associated with working memory deficits [42]. According to previous studies, there is a correlation between depression, anxiety, and overall cognitive function [43] and there was an interaction between depression and cognitive function on quality of life [44]. In this study, COVID-19 confirmation had a negative effect on depression, insomnia, and quality of life, like in previous studies [45]. It can be interpreted that COVID-19 has a negative impact on prospective memory, depression, insomnia, and quality of life. However, there was no significant effect on anxiety or COVID-19 fear. The COVID pandemic reported anxiety and fear not only in confirmed cases but also in the public [46, 47]. The results of this study also suggest that there was no significant difference in anxiety and fear between the COVID group, which is a confirmed case, and the non-COVID group, which is the public.

This study had some limitations. We used self-reported measures from an online survey. Self-reported surveys can cause socially desirable responses, recall bias, and misunderstandings of questions. In addition, it has been implemented online, and it may not be accessible to some older populations. However, we collected data in the same manner and minimized bias by using a large sample. In this study, the subjective memory complaints (SMCs) of the participants were measured, not objective outcome measures. Unlike objective assessments, subjective complaints tend to be overestimated with age [48]. Therefore, it is necessary to evaluate the cognitive function of participants using standardized assessment tools. Nevertheless, SCMs are associated with objectively measured cognitive performance [49]. Finally, this study was conducted only in Korea; therefore, a cautious interpretation of the results is needed.

This study is meaningful in that lifestyle, cognition, mental health, and quality of life in COVID-confirmed and non-confirmed older adults were identified. Furthermore, this study revealed that when faced with a pandemic, prevention and intervention strategies are needed for memory, lifestyle, depression, insomnia, and quality of life in older adults.

## Conclusions

Confirmation of COVID affected prospective memory, physical activity, diet, and quality of life, as well as increased depression and insomnia in older adults. In addition, the COVID pandemic has caused declines in physical activity and participation among older adults. Therefore, interventions are required to improve the memory, lifestyle, and mental health of older adults with COVID-19.

### Electronic supplementary material

Below is the link to the electronic supplementary material.

Supplementary Material 1

Supplementary Material 2

## Data Availability

The datasets used and/or analysed during the current study available from the corresponding author on reasonable request.
