# Supplementary material for: Comparison of lifestyle, cognitive function, mental health, and quality of life between hospitalized older adults with COVID-19 and non-COVID-19 in South Korea: a cross-sectional study
Source: BMC Geriatr. 2024 Apr 2;24:306. doi: 10.1186/s12877-023-04646-y (PMC10986076; doi:10.1186/s12877-023-04646-y)
Supplement: Supplementary file 1 — Supplementary Material 1 [file 12877_2023_4646_MOESM1_ESM.docx]

Supplementary File

| **The Yonsei Lifestyle Profile-BREF (English version)**  **Ⅰ. Physical activity**  The following are questions about physical activity. Indicate how many times you participate in the following activities per week.  Please consider your engagement in these activities before and after the onset of the COVID-19 pandemic and mark (∨) your answer on the box next to the choice that is most applicable for you.  **1.This is a question about aerobic exercise in the last week.**  (※Aerobic exercise: A relatively long, continuous breathing exercise, such as jogging, aerobics, jump rope, etc.)  How many days did you engage in aerobic exercises in the last week?  □**Never** □**1–2 days** □**3–4 days** □**5–6 days** □**Every day**  **2.This is a question about anaerobic exercise in the last week.**  (※ Anaerobic exercise: Short-duration workouts, such as sprinting and push-ups, which leave one short of breath, and are difficult to sustain.)  How many days did you engage in anaerobic exercise in the last week?  □**Never** □**1–2 days** □**3–4 days** □**5–6 days** □**Every day**  **3.This is a question about low-intensity physical activity in the last week.**  (※ Low-intensity physical activity: physical activity equivalent to 2**–**2.9 MET, such as gardening, house-cleaning, fishing, etc.)  How many days did you engage in low-intensity physical activity in the last week?  □**Never** □**1–2 days** □**3–4 days** □**5–6 days** □**Every day**  **4.This is a question about moderate-intensity physical activity in the last week.**  (※ Moderate-intensity physical activity: physical activity equivalent to 3–5.9 MET. such as swimming, doubles tennis, badminton, etc.)  How many days did you engage in moderate-intensity physical activity in the last week?  □**Never** □**1–2 days** □**3–4 days** □**5–6 days** □**Every day**  **5.This is a question about high-intensity physical activity in the last week.**  (※ High-intensity physical activity: physical activity equivalent to 6–9.9 MET, such as running, climbing, cycling, etc.)  How many days did you engage in high-intensity physical activity in the last week?  □**Never** □**1–2 days** □**3–4 days** □**5–6 days** □**Every day**  **Ⅱ. Activity participation**  The following are questions about your participation in certain activities. Indicate how often you have participated in the following physical activities in the last week.  Please consider your engagement in these activities before and after the onset of the COVID-19 pandemic, and mark (∨) your answer on the box next to the choice that is most applicable for you.  **1. This is a question about leisure activity in the last week.**  How many days did you participate in leisure activities in the last week?  □**Never** □**1–2 days** □**3–4 days** □**5–6 days** □**Every day**  **2. This is a question about social activity in the last week.**  How many days did you participate in social activities in the last week?  □**Never** □**1–2 days** □**3–4 days** □**5–6 days** □**Every day**  3. **This is a question about work in the last week.**  How many days did you participate in work in the last week?  □**Never** □**1–2 days** □**3–4 days** □**5–6 days** □**Every day**    **4.This is a question about education in the last week.**  How many days did you spend engaged in educational activities in the last week?  □**Never** □**1–2 days** □**3–4 days** □**5–6 days** □**Every day**  **Ⅲ. Nutrition**  The following are questions about food intake. Indicate how many times a week the following types of foods have been consumed in the last week.  Please consider your food consumption before and after the onset of the COVID-19 pandemic and mark (∨) your answer on the box next to the choice that is most applicable for you.  **1. Do you eat bread or flour food once or more times a day?**  □**Never** □**1–2 days** □**3–4 days** □**5–6 days** □**Every day**  **2. Do you eat sweet potatoes, potatoes, or corn once or more times a day?**  □**Never** □**1–2 days** □**3–4 days** □**5–6 days** □**Every day**  **3. Do you eat lean meat or chicken breast once or more times a day? * Meat: a loaf**  □**Never** □**1–2 days** □**3–4 days** □**5–6 days** □**Every day**  **4. Do you eat fish or tofu two or more times a day?**  □**Never** □**1–2 days** □**3–4 days** □**5–6 days** □**Every day**  **5. Do you eat beans or eggs once or more times a day?**  □**Never** □**1–2 days** □**3–4 days** □**5–6 days** □**Every day**  **6. Do you eat sesame or perilla oil once or more a day?**  □**Never** □**1–2 days** □**3–4 days** □**5–6 days** □**Every day**  **7. Do you eat nuts once or more times a day? * Nuts: pine nuts, sesame seeds, walnuts, peanuts, almonds, etc.**  □**Never** □**1–2 days** □**3–4 days** □**5–6 days** □**Every day**  **8. Do you eat green vegetables or kimchi once or more times a day? * Green vegetables: leek, spinach, radish, cabbage, etc.**  □**Never** □**1–2 days** □**3–4 days** □**5–6 days** □**Every day**  **9. Do you eat fruit once or more times a day? *Fruits: Apples, pears, melons, watermelons, tangerines, grapes, etc.**  □**Never** □**1–2 days** □**3–4 days** □**5–6 days** □**Every day**  **10. Do you eat seaweed once or more times a day? *Seaweed: Seaweed, kelp, seaweed, laver, etc.**  □**Never** □**1–2 days** □**3–4 days** □**5–6 days** □**Every day**  **11. Do you eat white milk (soy milk) or yoplait (yogurt) once or more times a day?**  □**Never** □**1–2 days** □**3–4 days** □**5–6 days** □**Every day**  **12. Do you eat cheese once or more times a day?**  □**Never** □**1–2 days** □**3–4 days** □**5–6 days** □**Every day**  **13. Do you eat anchovies or small prawns once or more times a day?**  □**Never** □**1–2 days** □**3–4 days** □**5–6 days** □**Every day** |
| --- |
