# Supplementary material for: Comparison of lifestyle, cognitive function, mental health, and quality of life between hospitalized older adults with COVID-19 and non-COVID-19 in South Korea: a cross-sectional study
Source: BMC Geriatr. 2024 Apr 2;24:306. doi: 10.1186/s12877-023-04646-y (PMC10986076; doi:10.1186/s12877-023-04646-y)

Group=1

Gender	Frequency	Percent	Cumulative Frequency	Cumulative Percent
1	92	48.68	92	48.68
2	97	51.32	189	100.00

Study	Frequency	Percent	Cumulative Frequency	Cumulative Percent
2	2	1.06	2	1.06
3	4	2.12	6	3.17
4	54	28.57	60	31.75
5	129	68.25	189	100.00

Occupation	Frequency	Percent	Cumulative Frequency	Cumulative Percent
1	108	57.14	108	57.14
2	81	42.86	189	100.00

HTN	Frequency	Percent	Cumulative Frequency	Cumulative Percent
1	122	64.55	122	64.55
2	67	35.45	189	100.00

DM	Frequency	Percent	Cumulative Frequency	Cumulative Percent
1	169	89.42	169	89.42
2	20	10.58	189	100.00

HL	Frequency	Percent	Cumulative Frequency	Cumulative Percent
1	169	89.42	169	89.42
2	20	10.58	189	100.00

Cerebrovascular	Frequency	Percent	Cumulative Frequency	Cumulative Percent
1	189	100.00	189	100.00

Cardiovascular	Frequency	Percent	Cumulative Frequency	Cumulative Percent
1	185	97.88	185	97.88
2	4	2.12	189	100.00

Group=1

etc	Frequency	Percent	Cumulative Frequency	Cumulative Percent
□□□	32	16.93	32	16.93
1	156	82.54	188	99.47
B□	1	0.53	189	100.00

Group=2

Gender	Frequency	Percent	Cumulative Frequency	Cumulative Percent
1	39	35.14	39	35.14
2	72	64.86	111	100.00

Study	Frequency	Percent	Cumulative Frequency	Cumulative Percent
1	38	34.23	38	34.23
2	19	17.12	57	51.35
3	19	17.12	76	68.47
4	22	19.82	98	88.29
5	13	11.71	111	100.00

Occupation	Frequency	Percent	Cumulative Frequency	Cumulative Percent
1	62	55.86	62	55.86
2	38	34.23	100	90.09
3	11	9.91	111	100.00

HTN	Frequency	Percent	Cumulative Frequency	Cumulative Percent
1	62	55.86	62	55.86
2	49	44.14	111	100.00

DM	Frequency	Percent	Cumulative Frequency	Cumulative Percent
1	84	75.68	84	75.68
2	27	24.32	111	100.00

HL	Frequency	Percent	Cumulative Frequency	Cumulative Percent
1	77	69.37	77	69.37
2	34	30.63	111	100.00

Group=2

Cerebrovascular	Frequency	Percent	Cumulative Frequency	Cumulative Percent
**1**	103	92.79	103	92.79
**2**	8	7.21	111	100.00

Cardiovascular	Frequency	Percent	Cumulative Frequency	Cumulative Percent
**1**	91	81.98	91	81.98
**2**	20	18.02	111	100.00

etc	Frequency	Percent	Cumulative Frequency	Cumulative Percent
▯	21	18.92	21	18.92
**1**	90	81.08	111	100.00

Frequency<br>Expected<br>Percent<br>Row Pct<br>Col Pct

Table of Group by Gender

Group	Gender: 1	Gender: 2	Total
**1**	92<br>82.53<br>30.67<br>48.68<br>70.23	97<br>106.47<br>32.33<br>51.32<br>57.40	189<br><br>63.00
**2**	39<br>48.47<br>13.00<br>35.14<br>29.77	72<br>62.53<br>24.00<br>64.86<br>42.60	111<br><br>37.00
**Total**	131<br>43.67	169<br>56.33	300<br>100.00

Statistics for Table of Group by Gender

Statistic	DF	Value	Prob
Chi-Square	1	5.2134	0.0224
Likelihood Ratio Chi-Square	1	5.2667	0.0217
Continuity Adj. Chi-Square	1	4.6774	0.0306
Mantel-Haenszel Chi-Square	1	5.1960	0.0226
Phi Coefficient		0.1318	
Contingency Coefficient		0.1307	
Cramer's V		0.1318	

Fisher's Exact Test	
Cell (1,1) Frequency (F)	92
Left-sided Pr <= F	0.9921
Right-sided Pr >= F	0.0150
Table Probability (P)	0.0071
Two-sided Pr <= P	0.0298

Sample Size = 300

Frequency<br>Expected<br>Percent<br>Row Pct<br>Col Pct

Table of Group by Study						
	Study					
Group	1	2	3	4	5	Total
1	0<br>23.94<br>0.00<br>0.00<br>0.00	2<br>13.23<br>0.67<br>1.06<br>9.52	4<br>14.49<br>1.33<br>2.12<br>17.39	54<br>47.88<br>18.00<br>28.57<br>71.05	129<br>89.46<br>43.00<br>68.25<br>90.85	189<br><br>63.00
2	38<br>14.06<br>12.67<br>34.23<br>100.00	19<br>7.77<br>6.33<br>17.12<br>90.48	19<br>8.51<br>6.33<br>17.12<br>82.61	22<br>28.12<br>7.33<br>19.82<br>28.95	13<br>52.54<br>4.33<br>11.71<br>9.15	111<br><br>37.00
Total	38<br>12.67	21<br>7.00	23<br>7.67	76<br>25.33	142<br>47.33	300<br>100.00

Statistics for Table of Group by Study

Statistic	DF	Value	Prob
Chi-Square	4	160.3376	<.0001
Likelihood Ratio Chi-Square	4	182.5211	<.0001
Mantel-Haenszel Chi-Square	1	151.8376	<.0001
Phi Coefficient		0.7311	
Contingency Coefficient		0.5902	
Cramer's V		0.7311	

Sample Size = 300

Frequency<br>Expected<br>Percent<br>Row Pct<br>Col Pct

Table of Group by Occupation				
	Occupation			
Group	1	2	3	Total
1	108<br>107.1<br>36.00<br>57.14<br>63.53	81<br>74.97<br>27.00<br>42.86<br>68.07	0<br>6.93<br>0.00<br>0.00<br>0.00	189<br><br>63.00
2	62<br>62.9<br>20.67<br>55.86<br>36.47	38<br>44.03<br>12.67<br>34.23<br>31.93	11<br>4.07<br>3.67<br>9.91<br>100.00	111<br><br>37.00
Total	170<br>56.67	119<br>39.67	11<br>3.67	300<br>100.00

Statistics for Table of Group by Occupation

Statistic	DF	Value	Prob
Chi-Square	2	20.0610	<.0001
Likelihood Ratio Chi-Square	2	23.2329	<.0001
Mantel-Haenszel Chi-Square	1	2.7100	0.0997
Phi Coefficient		0.2586	
Contingency Coefficient		0.2504	
Cramer's V		0.2586	

Sample Size = 300

Frequency<br>Expected<br>Percent<br>Row Pct<br>Col Pct

Table of Group by HTN

Group	HTN 1	HTN 2	Total
1	122<br>115.92<br>40.67<br>64.55<br>66.30	67<br>73.08<br>22.33<br>35.45<br>57.76	189<br><br>63.00
2	62<br>68.08<br>20.67<br>55.86<br>33.70	49<br>42.92<br>16.33<br>44.14<br>42.24	111<br><br>37.00
Total	184<br>61.33	116<br>38.67	300<br>100.00

Statistics for Table of Group by HTN

Statistic	DF	Value	Prob
Chi-Square	1	2.2290	0.1354
Likelihood Ratio Chi-Square	1	2.2171	0.1365
Continuity Adj. Chi-Square	1	1.8775	0.1706
Mantel-Haenszel Chi-Square	1	2.2216	0.1361
Phi Coefficient		0.0862	
Contingency Coefficient		0.0859	
Cramer's V		0.0862	

Fisher's Exact Test	
Cell (1,1) Frequency (F)	122
Left-sided Pr <= F	0.9466
Right-sided Pr >= F	0.0856
Table Probability (P)	0.0322
Two-sided Pr <= P	0.1425

Sample Size = 300

Frequency<br>Expected<br>Percent<br>Row Pct<br>Col Pct

Table of Group by DM			
	DM		
Group	1	2	Total
1	169<br>159.39<br>56.33<br>89.42<br>66.80	20<br>29.61<br>6.67<br>10.58<br>42.55	189<br><br>63.00
2	84<br>93.61<br>28.00<br>75.68<br>33.20	27<br>17.39<br>9.00<br>24.32<br>57.45	111<br><br>37.00
Total	253<br>84.33	47<br>15.67	300<br>100.00

Statistics for Table of Group by DM

Statistic	DF	Value	Prob
Chi-Square	1	9.9956	0.0016
Likelihood Ratio Chi-Square	1	9.6519	0.0019
Continuity Adj. Chi-Square	1	8.9825	0.0027
Mantel-Haenszel Chi-Square	1	9.9622	0.0016
Phi Coefficient		0.1825	
Contingency Coefficient		0.1796	
Cramer's V		0.1825	

Fisher's Exact Test	
Cell (1,1) Frequency (F)	169
Left-sided Pr <= F	0.9995
Right-sided Pr >= F	0.0016
Table Probability (P)	0.0010
Two-sided Pr <= P	0.0027

Sample Size = 300

Frequency<br>Expected<br>Percent<br>Row Pct<br>Col Pct

Table of Group by HL

	HL		
**Group**	**1**	**2**	**Total**
**1**	169<br>154.98<br>56.33<br>89.42<br>68.70	20<br>34.02<br>6.67<br>10.58<br>37.04	189<br><br>63.00
**2**	77<br>91.02<br>25.67<br>69.37<br>31.30	34<br>19.98<br>11.33<br>30.63<br>62.96	111<br><br>37.00
**Total**	246<br>82.00	54<br>18.00	300<br>100.00

Statistics for Table of Group by HL

Statistic	DF	Value	Prob
Chi-Square	1	19.0435	<.0001
Likelihood Ratio Chi-Square	1	18.4136	<.0001
Continuity Adj. Chi-Square	1	17.7094	<.0001
Mantel-Haenszel Chi-Square	1	18.9800	<.0001
Phi Coefficient		0.2519	
Contingency Coefficient		0.2443	
Cramer's V		0.2519	

Fisher's Exact Test	
Cell (1,1) Frequency (F)	169
Left-sided Pr <= F	1.0000
Right-sided Pr >= F	<.0001
Table Probability (P)	<.0001
Two-sided Pr <= P	<.0001

Sample Size = 300

Frequency<br>Expected<br>Percent<br>Row Pct<br>Col Pct

Table of Group by Cerebrovascular			
	Cerebrovascular		
Group	1	2	Total
1	189<br>183.96<br>63.00<br>100.00<br>64.73	0<br>5.04<br>0.00<br>0.00<br>0.00	189<br><br>63.00
2	103<br>108.04<br>34.33<br>92.79<br>35.27	8<br>2.96<br>2.67<br>7.21<br>100.00	111<br><br>37.00
Total	292<br>97.33	8<br>2.67	300<br>100.00

Statistics for Table of Group by Cerebrovascular

Statistic	DF	Value	Prob
Chi-Square	1	13.9948	0.0002
Likelihood Ratio Chi-Square	1	16.2837	<.0001
Continuity Adj. Chi-Square	1	11.3558	0.0008
Mantel-Haenszel Chi-Square	1	13.9482	0.0002
Phi Coefficient		0.2160	
Contingency Coefficient		0.2111	
Cramer's V		0.2160	
WARNING: 25% of the cells have expected counts less than 5. Chi-Square may not be a valid test.			

Fisher's Exact Test	
Cell (1,1) Frequency (F)	189
Left-sided Pr <= F	1.0000
Right-sided Pr >= F	0.0003
Table Probability (P)	0.0003
Two-sided Pr <= P	0.0003

Sample Size = 300

Frequency<br>Expected<br>Percent<br>Row Pct<br>Col Pct

Table of Group by Cardiovascular			
	Cardiovascular		
Group	1	2	Total
1	185<br>173.88<br>61.67<br>97.88<br>67.03	4<br>15.12<br>1.33<br>2.12<br>16.67	189<br><br>63.00
2	91<br>102.12<br>30.33<br>81.98<br>32.97	20<br>8.88<br>6.67<br>18.02<br>83.33	111<br><br>37.00
Total	276<br>92.00	24<br>8.00	300<br>100.00

Statistics for Table of Group by Cardiovascular

Statistic	DF	Value	Prob
Chi-Square	1	24.0253	<.0001
Likelihood Ratio Chi-Square	1	23.7933	<.0001
Continuity Adj. Chi-Square	1	21.9133	<.0001
Mantel-Haenszel Chi-Square	1	23.9452	<.0001
Phi Coefficient		0.2830	
Contingency Coefficient		0.2723	
Cramer's V		0.2830	

Fisher's Exact Test	
Cell (1,1) Frequency (F)	185
Left-sided Pr <= F	1.0000
Right-sided Pr >= F	<.0001
Table Probability (P)	<.0001
Two-sided Pr <= P	<.0001

Sample Size = 300

Group	N Obs	Variable	N	Mean	Std Dev	Minimum	Maximum
1	189	Age	189	61.4126984	5.8043498	55.0000000	78.0000000
		YLPPA_PRE	189	11.2328042	3.4408038	5.0000000	25.0000000
		YLPPA_POST	189	9.8253968	3.3968368	5.0000000	22.0000000
		YLPPA_Diff	189	1.4074074	3.1737199	-9.0000000	12.0000000
		YLPAP_PRE	189	8.4761905	2.3351419	4.0000000	19.0000000
		YLPAP_POST	189	6.7671958	1.9043017	4.0000000	14.0000000
		YLPAP_Diff	189	1.7089947	2.0355807	-5.0000000	8.0000000
		YLPDIET_PRE	189	33.3492063	7.7682361	20.0000000	55.0000000
		YLPDIET_POST	189	33.0052910	7.7249922	16.0000000	55.0000000
		YLPDIET_Diff	189	0.3439153	4.5794671	-16.0000000	25.0000000
		VASD	189	4.3756614	2.3882782	1.0000000	10.0000000
		PHQ	189	4.1375661	4.0974975	0	20.0000000
		VASA	189	5.2063492	2.0276708	1.0000000	9.0000000
		SAS	189	36.2328042	7.3375153	23.0000000	70.0000000
		ISI	189	8.5767196	6.2544187	0	28.0000000
		FCV	189	17.4814815	5.9444168	7.0000000	34.0000000
		WHOQOL	189	84.0000000	13.9969602	49.0000000	118.0000000
		PRMQ	189	30.1693122	8.6800646	16.0000000	58.0000000
		PM	189	15.4708995	4.3485691	8.0000000	28.0000000
		RM	189	14.6984127	4.6102393	8.0000000	30.0000000
		SMCQ	189	2.6402116	2.5511099	0	14.0000000
2	111	Age	111	68.6306306	7.8727700	54.0000000	86.0000000
		YLPPA_PRE	111	8.9369369	2.1160825	4.0000000	18.0000000
		YLPPA_POST	111	5.1621622	0.5960558	5.0000000	9.0000000
		YLPPA_Diff	111	3.7747748	2.1049063	-1.0000000	13.0000000
		YLPAP_PRE	111	6.2072072	2.1705346	4.0000000	16.0000000
		YLPAP_POST	111	4.3153153	0.5874751	4.0000000	6.0000000
		YLPAP_Diff	111	1.8918919	2.0597941	-1.0000000	12.0000000
		YLPDIET_PRE	111	34.0180180	4.4558072	25.0000000	48.0000000
		YLPDIET_POST	111	29.2792793	3.3362232	16.0000000	41.0000000
		YLPDIET_Diff	111	4.7387387	5.0284856	-6.0000000	25.0000000
		VASD	111	4.8738739	2.1282392	0	10.0000000
		PHQ	111	5.1081081	4.0030701	0	19.0000000
		VASA	111	5.2972973	2.6236498	0	10.0000000
		SAS	111	37.0540541	7.3630994	21.0000000	56.0000000
		ISI	111	10.7027027	5.2006898	2.0000000	28.0000000
		FCV	111	17.4324324	5.3557473	7.0000000	35.0000000
		WHOQOL	111	89.0720721	15.2528458	54.0000000	123.0000000
		PRMQ	111	32.6036036	9.4851839	17.0000000	60.0000000
		PM	111	18.4774775	5.6469571	9.0000000	31.0000000
		RM	111	14.1261261	4.4926548	8.0000000	32.0000000
		SMCQ	111	2.9639640	2.6489378	0	10.0000000

Difference: YLPPA_PRE - YLPPA_POST

N	Mean	Std Dev	Std Err	Minimum	Maximum
189	1.4074	3.1737	0.2309	-9.0000	12.0000

Mean	95% CL Mean		Std Dev	95% CL Std Dev	
1.4074	0.9520	1.8628	3.1737	2.8828	3.5305

DF	t Value	Pr > |t|
188	6.10	<.0001

Difference: YLPPA_PRE - YLPPA_POST

Difference: YLPPA_PRE - YLPPA_POST

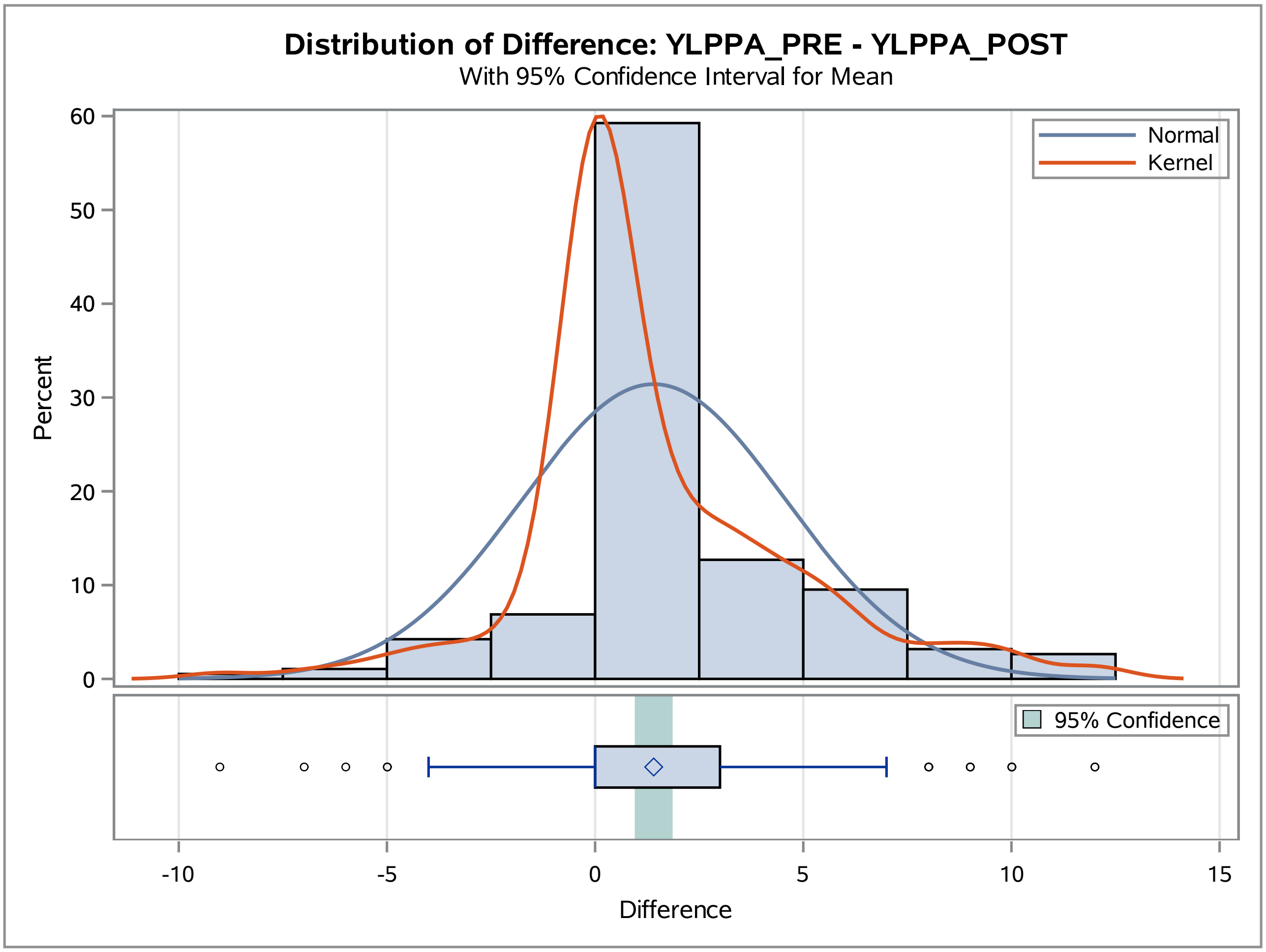

Difference: YLPPA_PRE - YLPPA_POST

Difference: YLPPA_PRE - YLPPA_POST

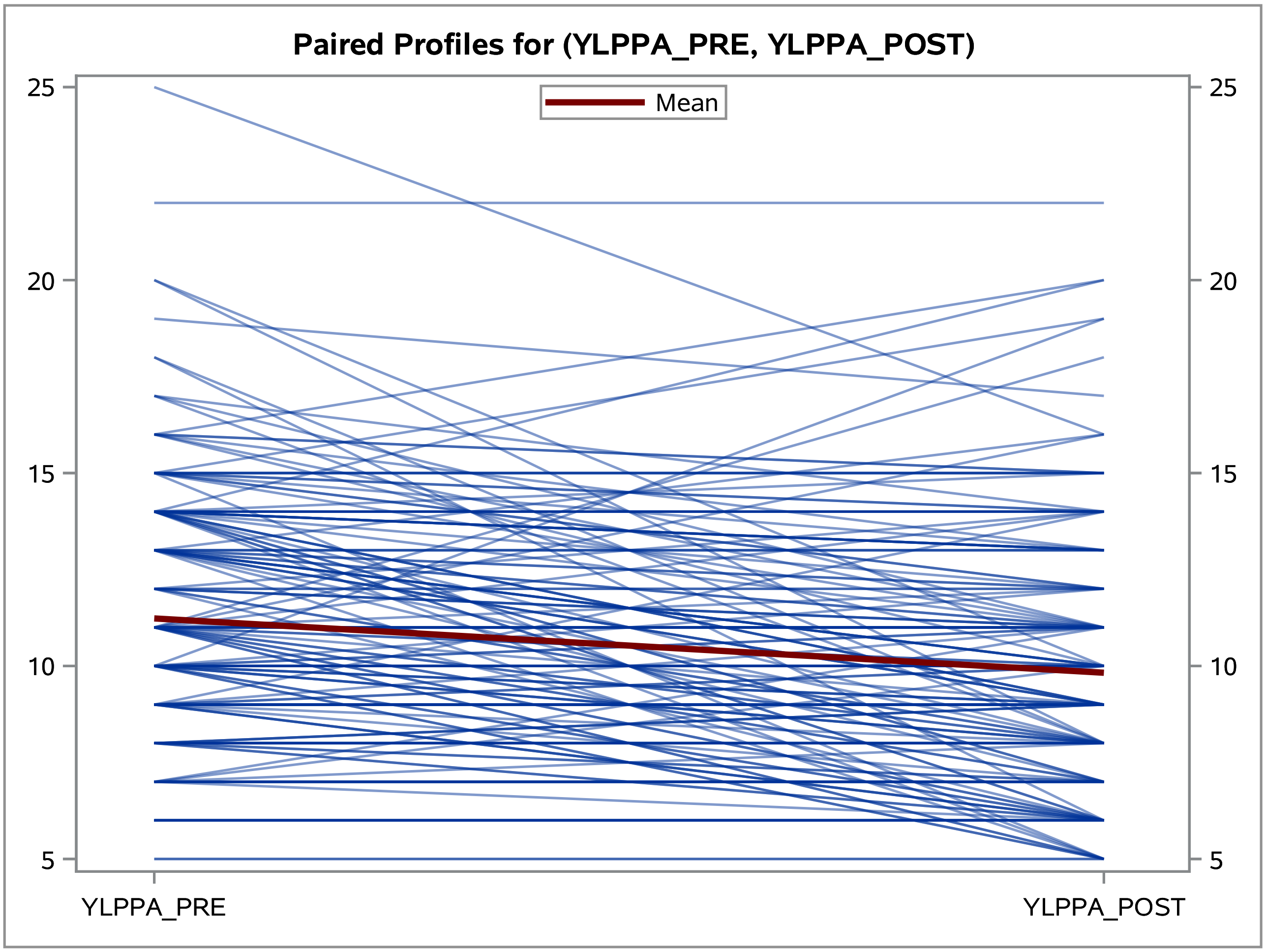

Difference: YLPPA_PRE - YLPPA_POST

Difference: YLPPA_PRE - YLPPA_POST

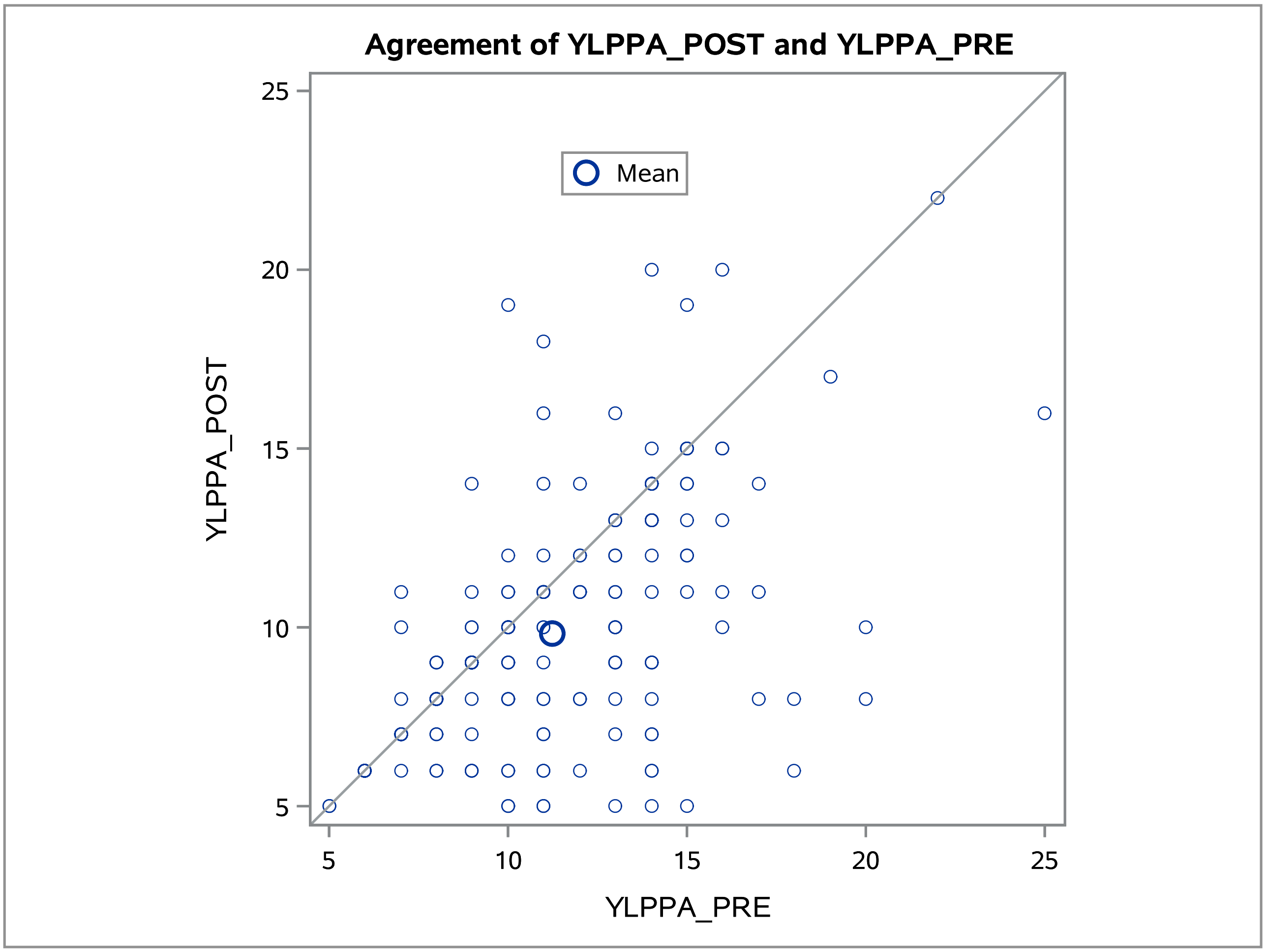

Difference: YLPPA_PRE - YLPPA_POST

Difference: YLPPA_PRE - YLPPA_POST

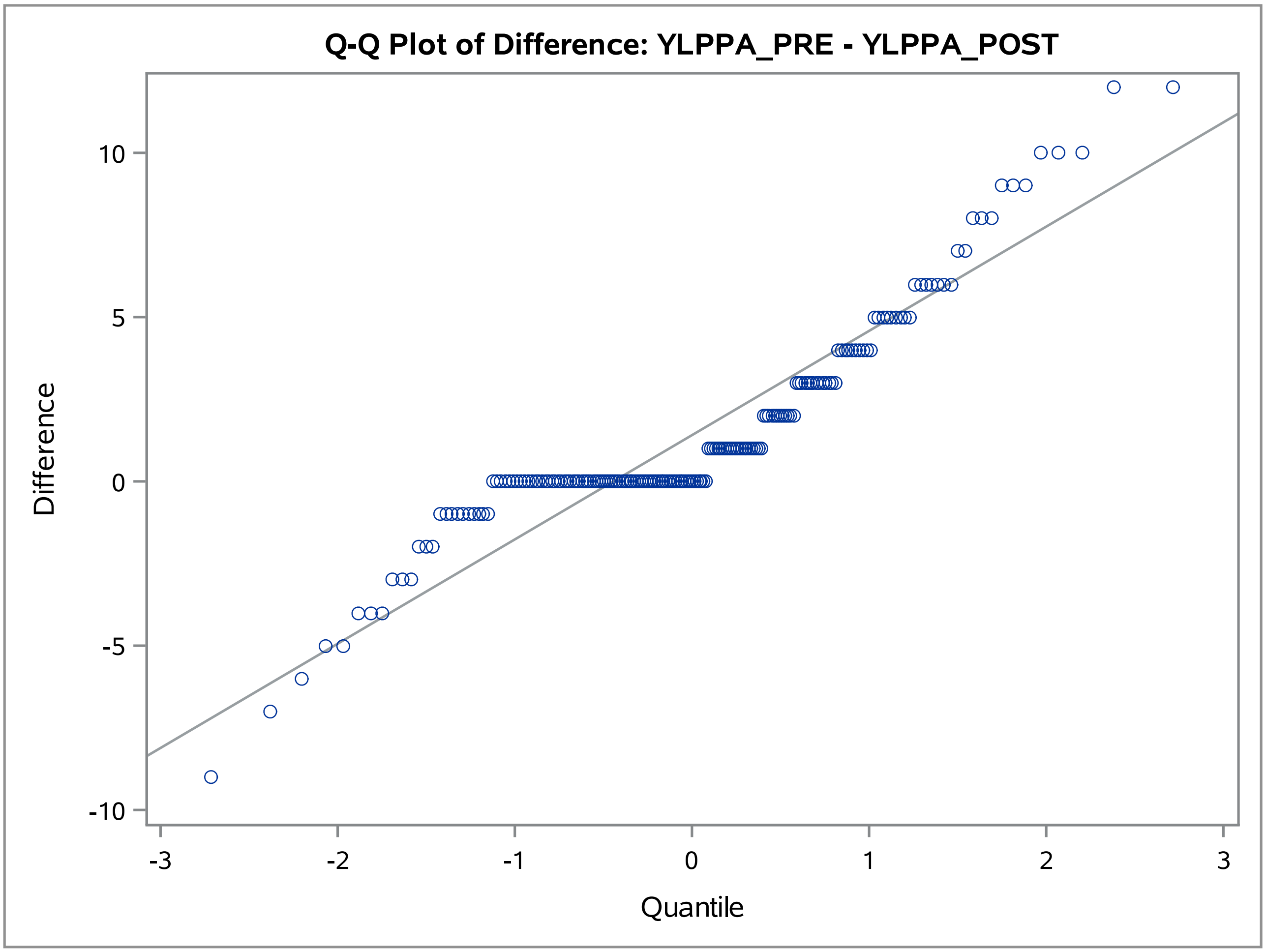

Difference: YLPPA_PRE - YLPPA_POST

N	Mean	Std Dev	Std Err	Minimum	Maximum
111	3.7748	2.1049	0.1998	-1.0000	13.0000

Mean	95% CL Mean		Std Dev	95% CL Std Dev	
3.7748	3.3788	4.1707	2.1049	1.8597	2.4251

DF	t Value	Pr > |t|
110	18.89	<.0001

Difference: YLPPA_PRE - YLPPA_POST

Difference: YLPPA_PRE - YLPPA_POST

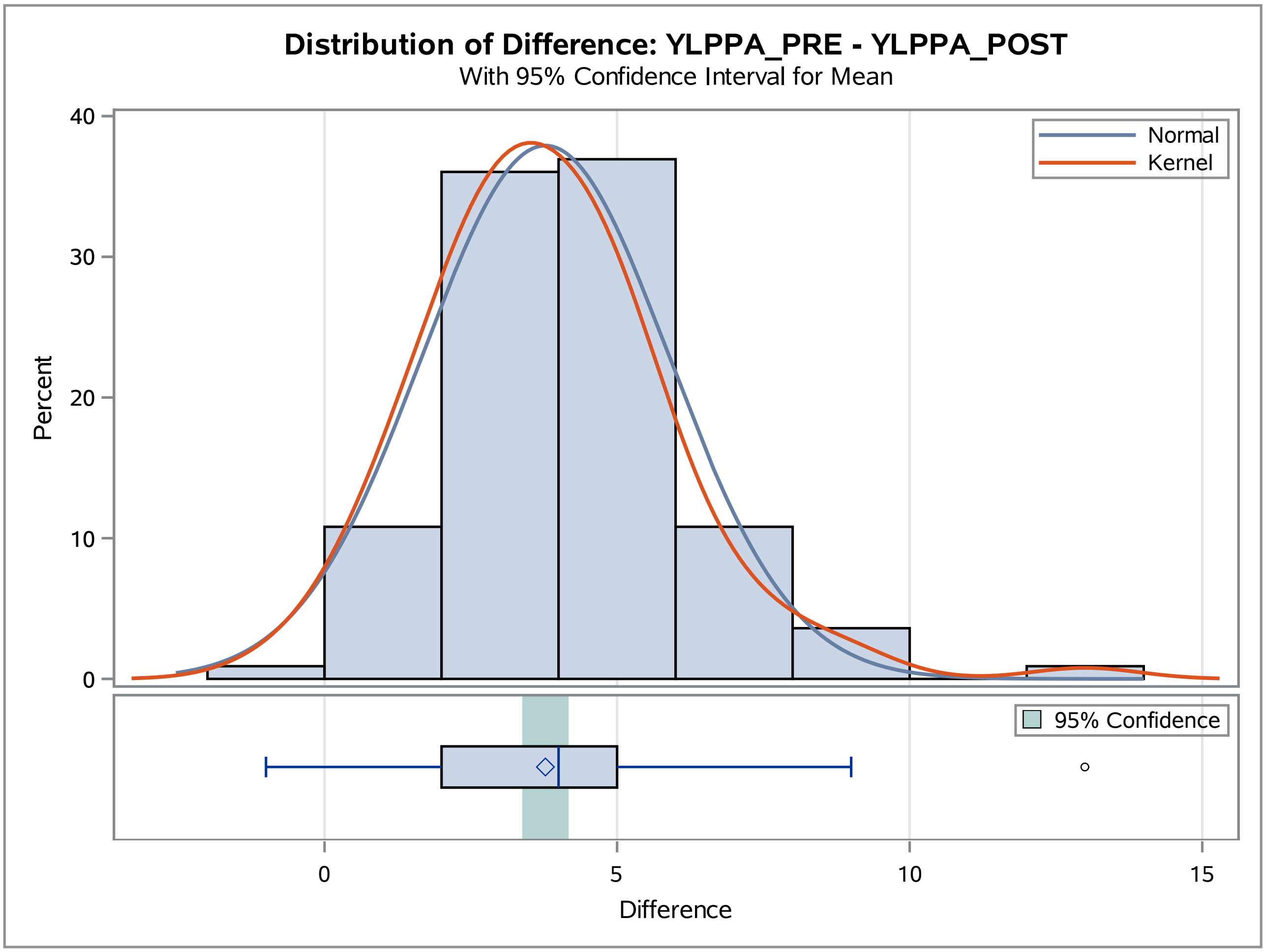

Difference: YLPPA_PRE - YLPPA_POST

Difference: YLPPA_PRE - YLPPA_POST

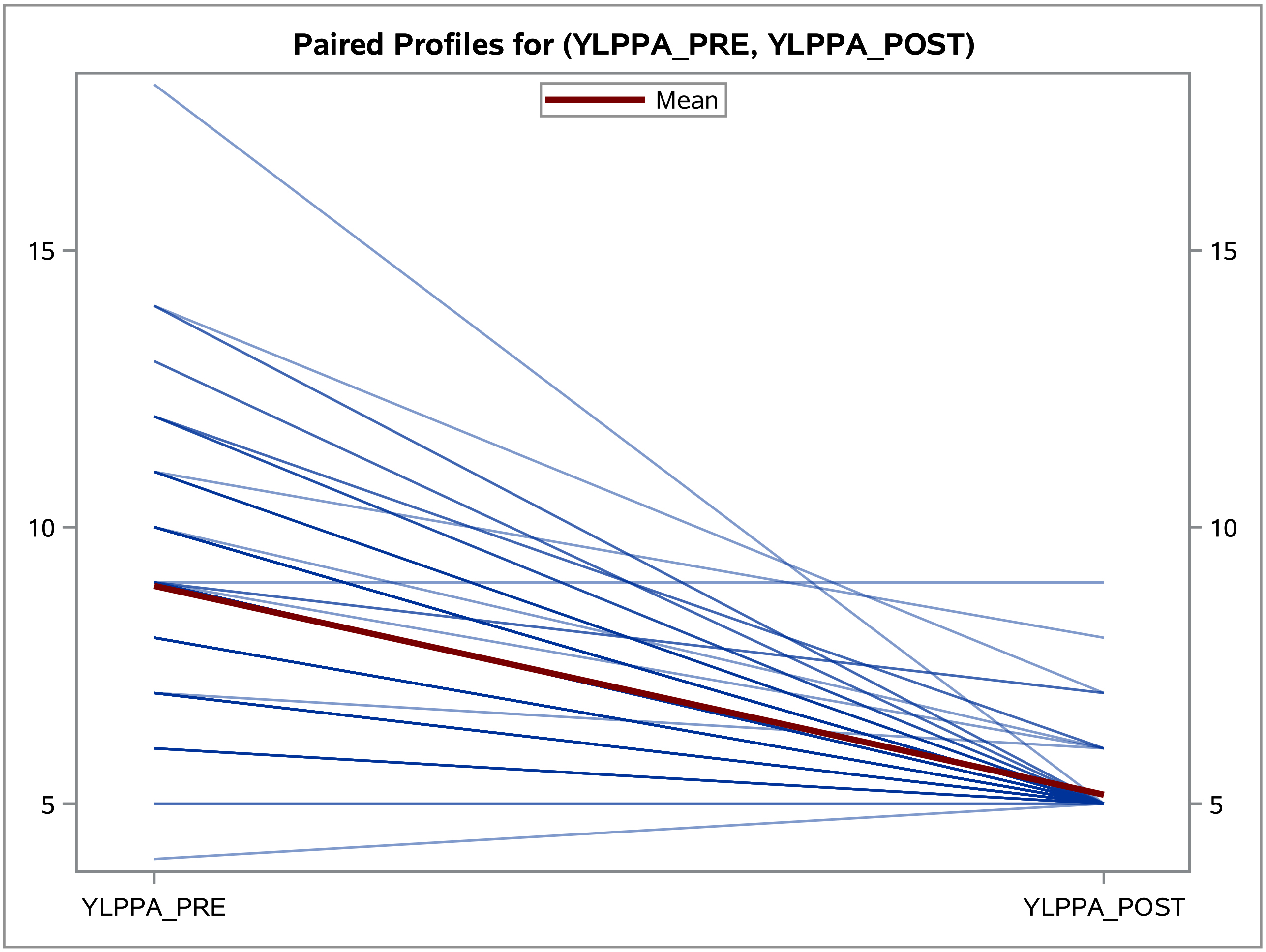

Difference: YLPPA_PRE - YLPPA_POST

Difference: YLPPA_PRE - YLPPA_POST

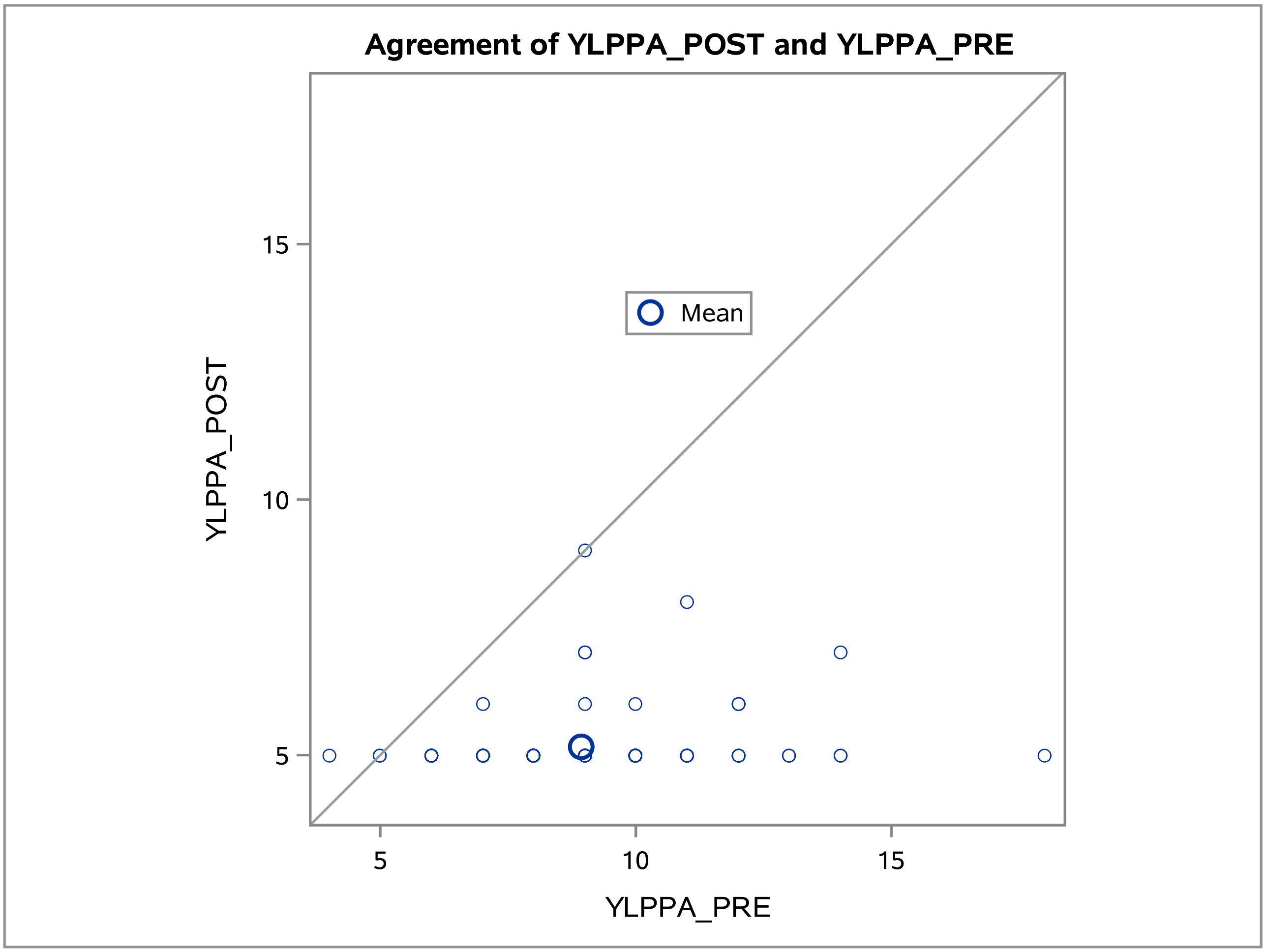

Difference: YLPPA_PRE - YLPPA_POST

Difference: YLPPA_PRE - YLPPA_POST

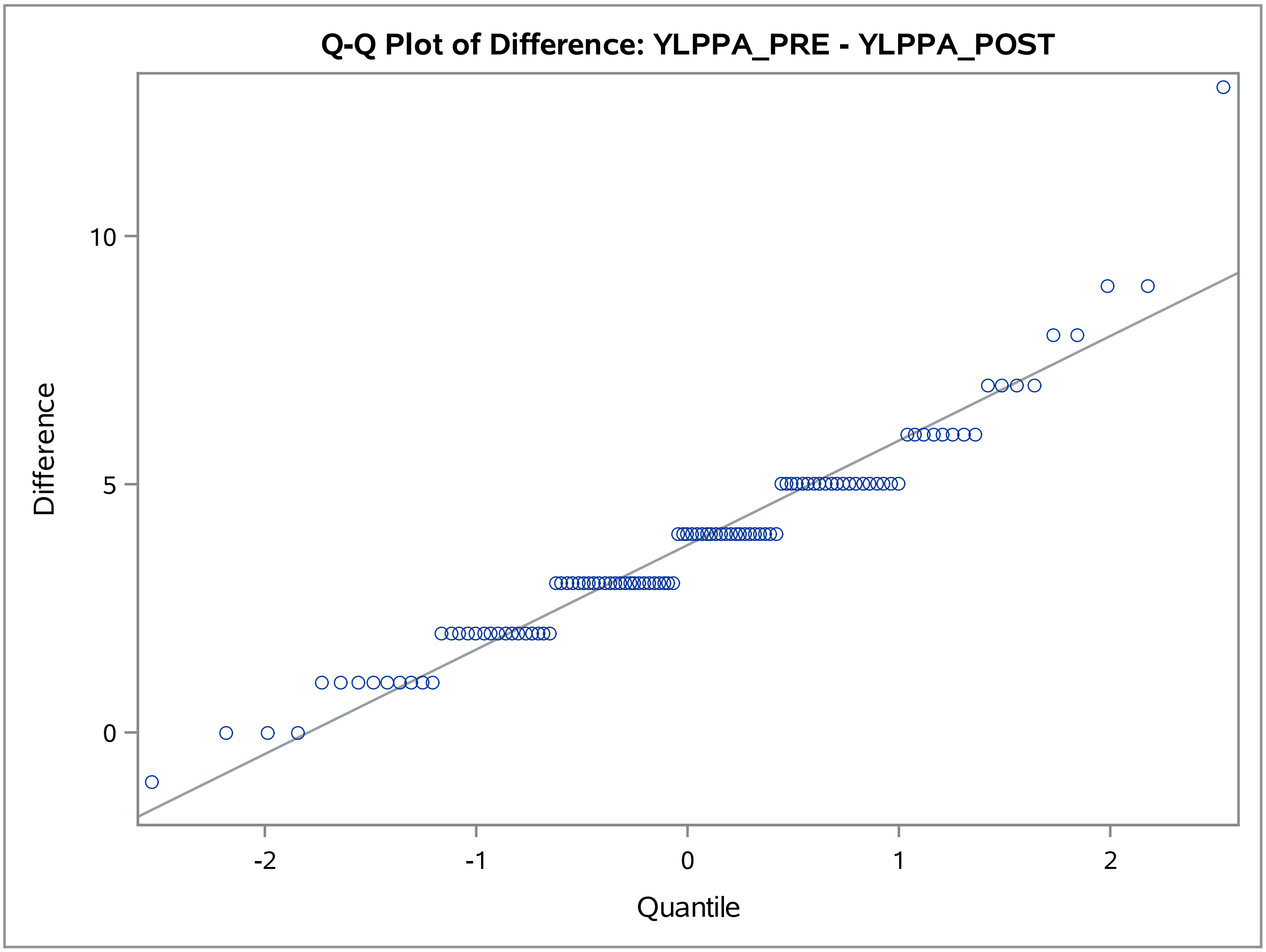

Difference: YLPAP_PRE - YLPAP_POST

N	Mean	Std Dev	Std Err	Minimum	Maximum
189	1.7090	2.0356	0.1481	-5.0000	8.0000

Mean	95% CL Mean		Std Dev	95% CL Std Dev	
1.7090	1.4169	2.0011	2.0356	1.8490	2.2644

DF	t Value	Pr > |t|
188	11.54	<.0001

Difference: YLPAP_PRE - YLPAP_POST

Difference: YLPAP_PRE - YLPAP_POST

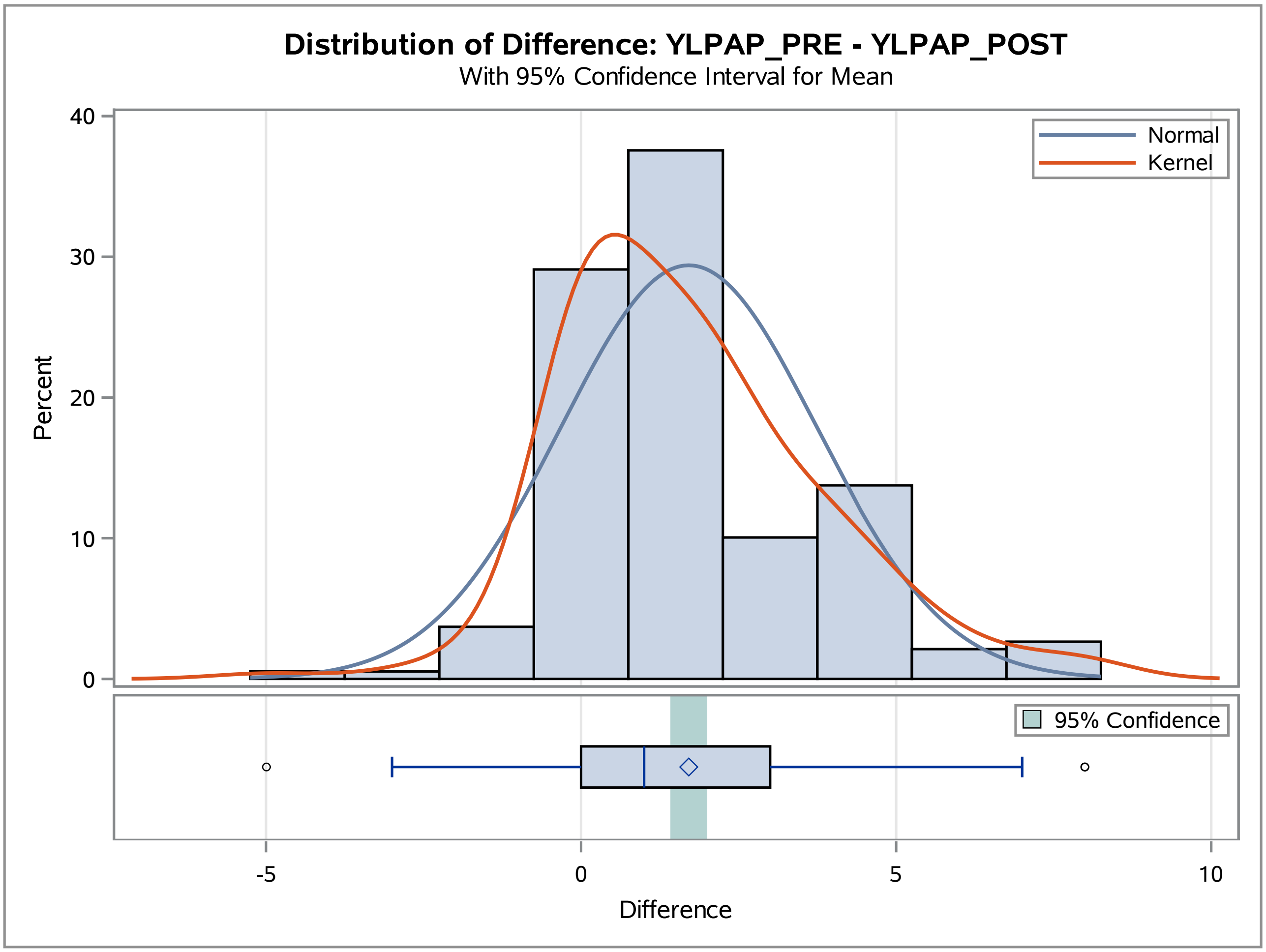

Difference: YLPAP_PRE - YLPAP_POST

Difference: YLPAP_PRE - YLPAP_POST

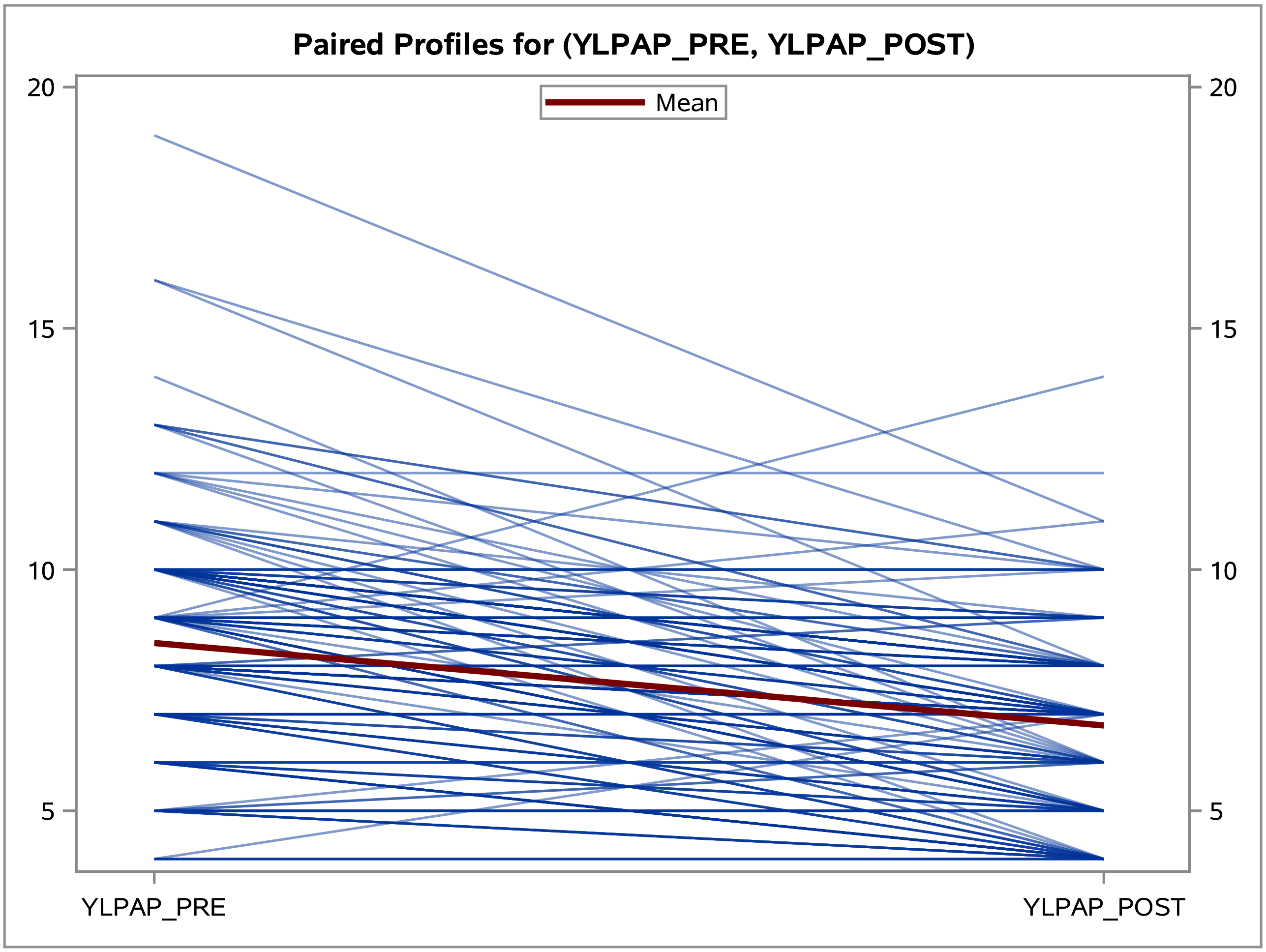

Difference: YLPAP_PRE - YLPAP_POST

Difference: YLPAP_PRE - YLPAP_POST

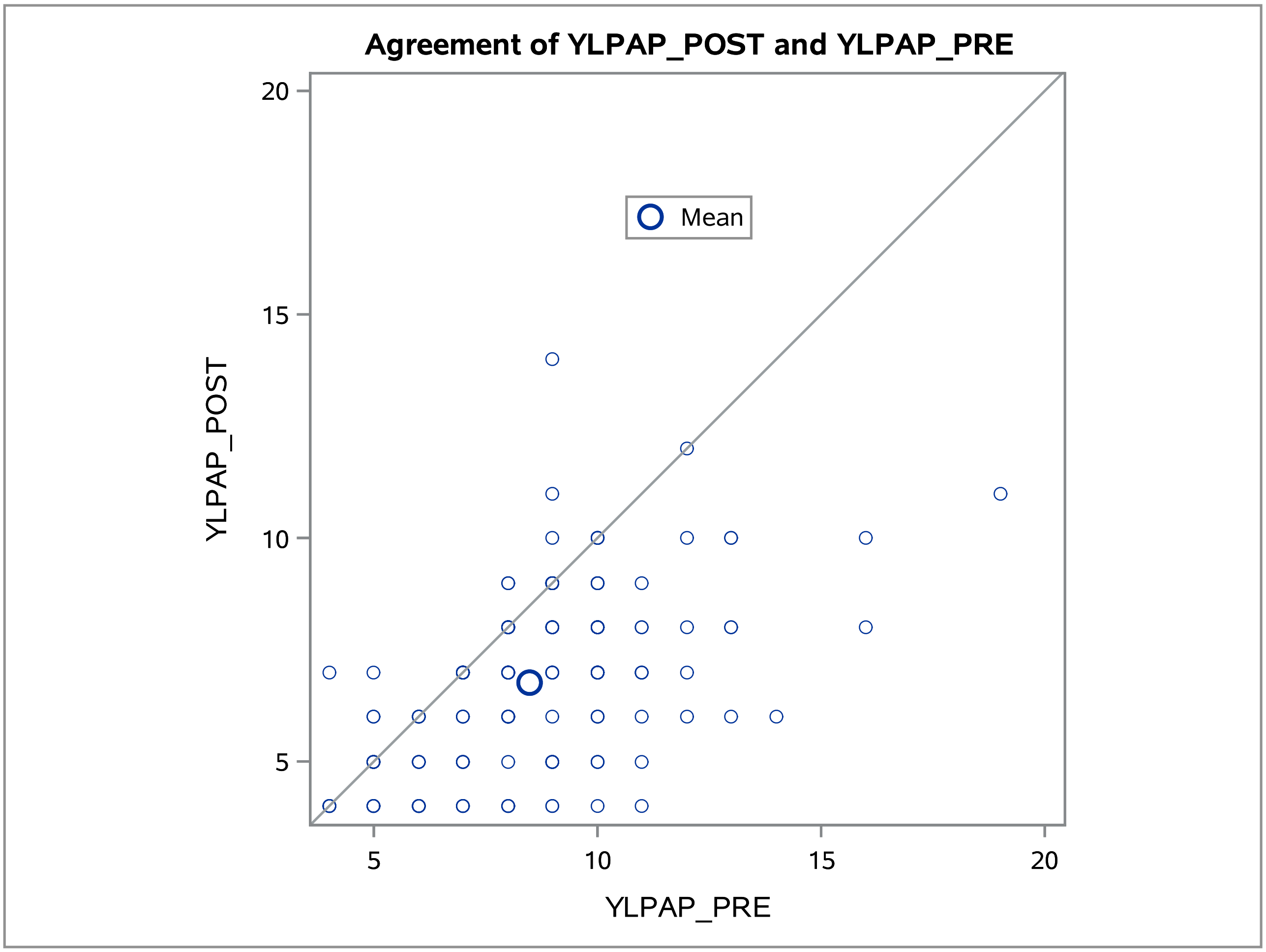

Difference: YLPAP_PRE - YLPAP_POST

Difference: YLPAP_PRE - YLPAP_POST

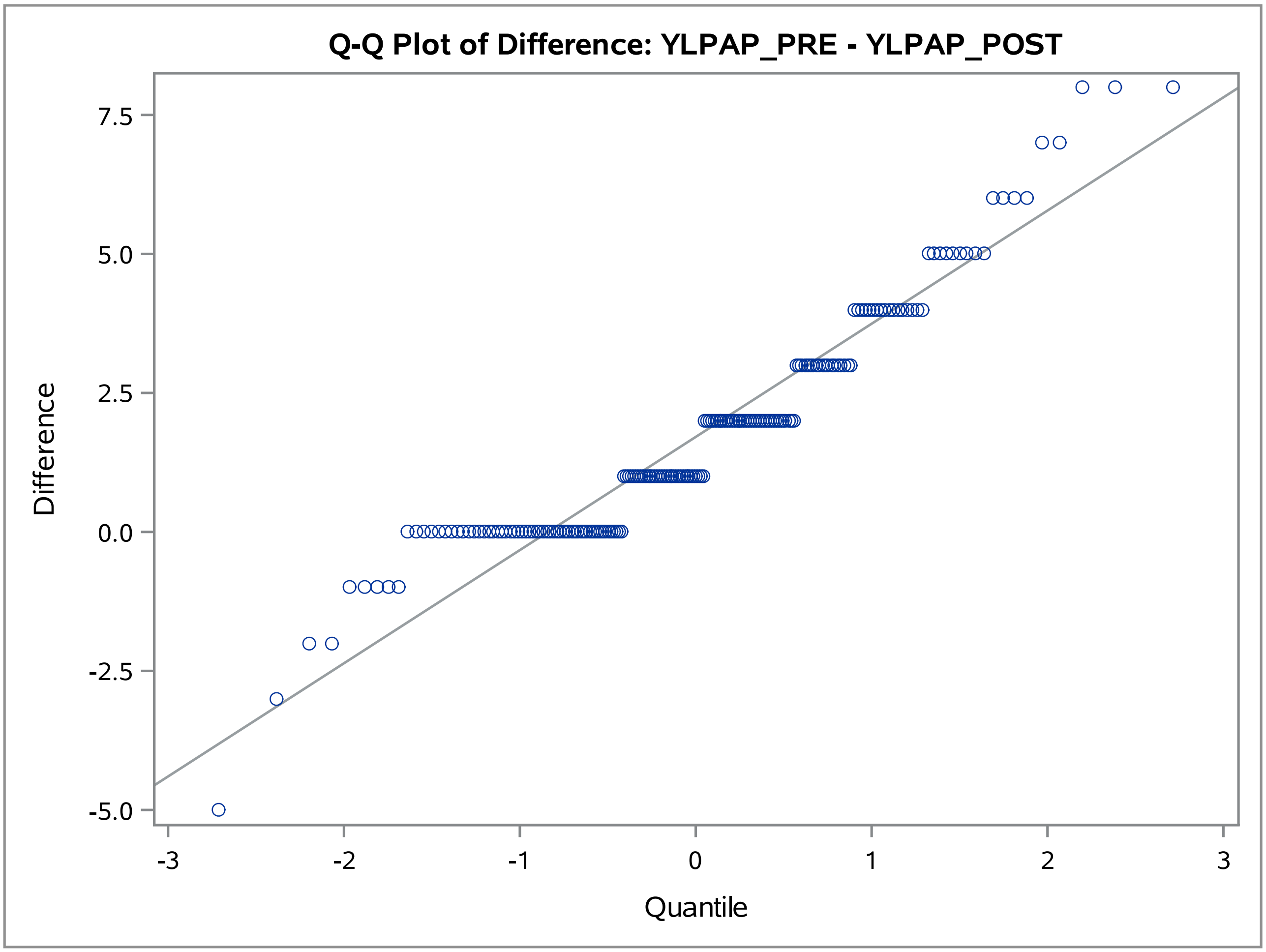

Difference: YLPAP_PRE - YLPAP_POST

N	Mean	Std Dev	Std Err	Minimum	Maximum
111	1.8919	2.0598	0.1955	-1.0000	12.0000

Mean	95% CL Mean		Std Dev	95% CL Std Dev	
1.8919	1.5044	2.2793	2.0598	1.8199	2.3732

DF	t Value	Pr > |t|
110	9.68	<.0001

Difference: YLPAP_PRE - YLPAP_POST

Difference: YLPAP_PRE - YLPAP_POST

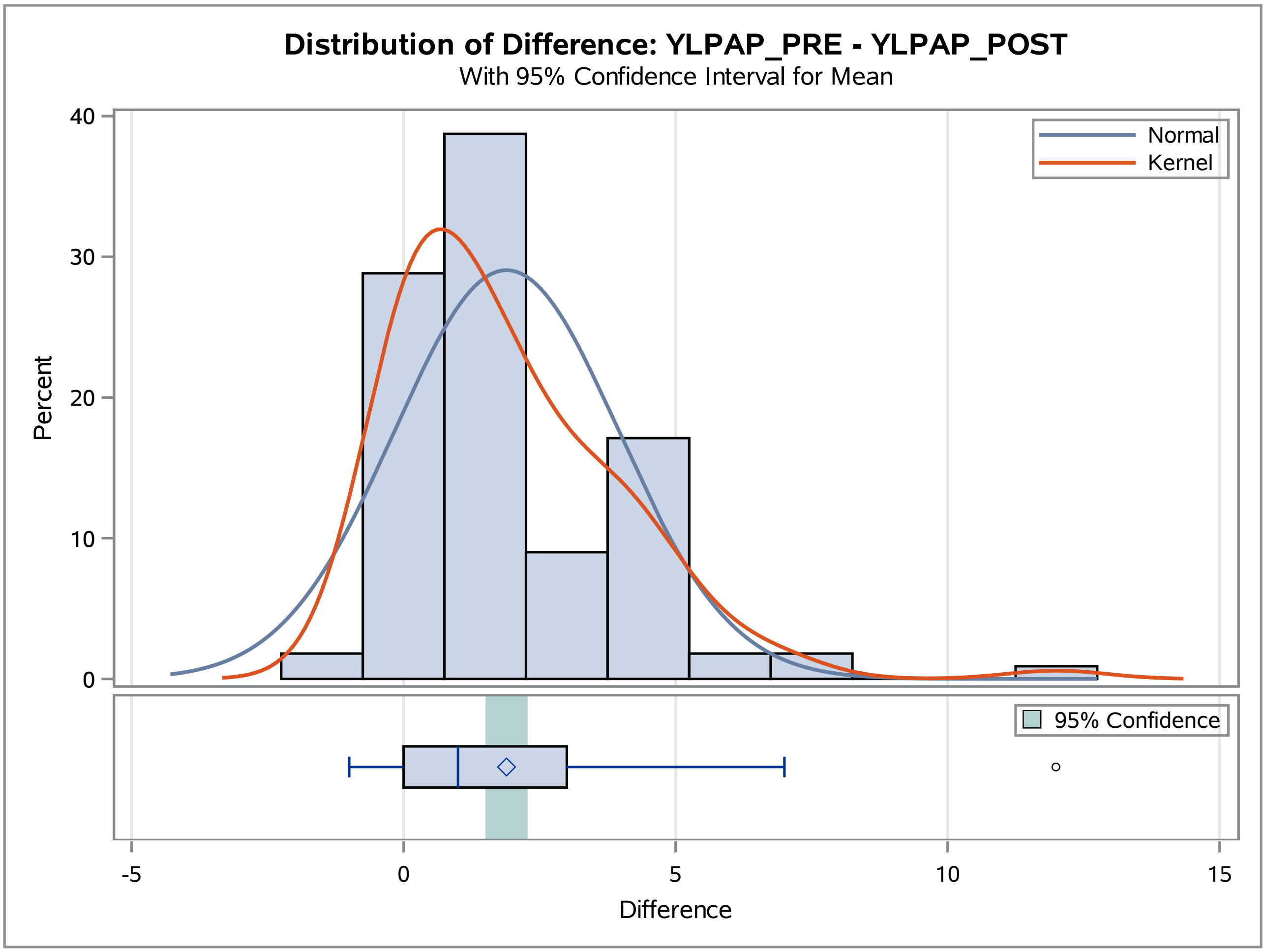

Difference: YLPAP_PRE - YLPAP_POST

Difference: YLPAP_PRE - YLPAP_POST

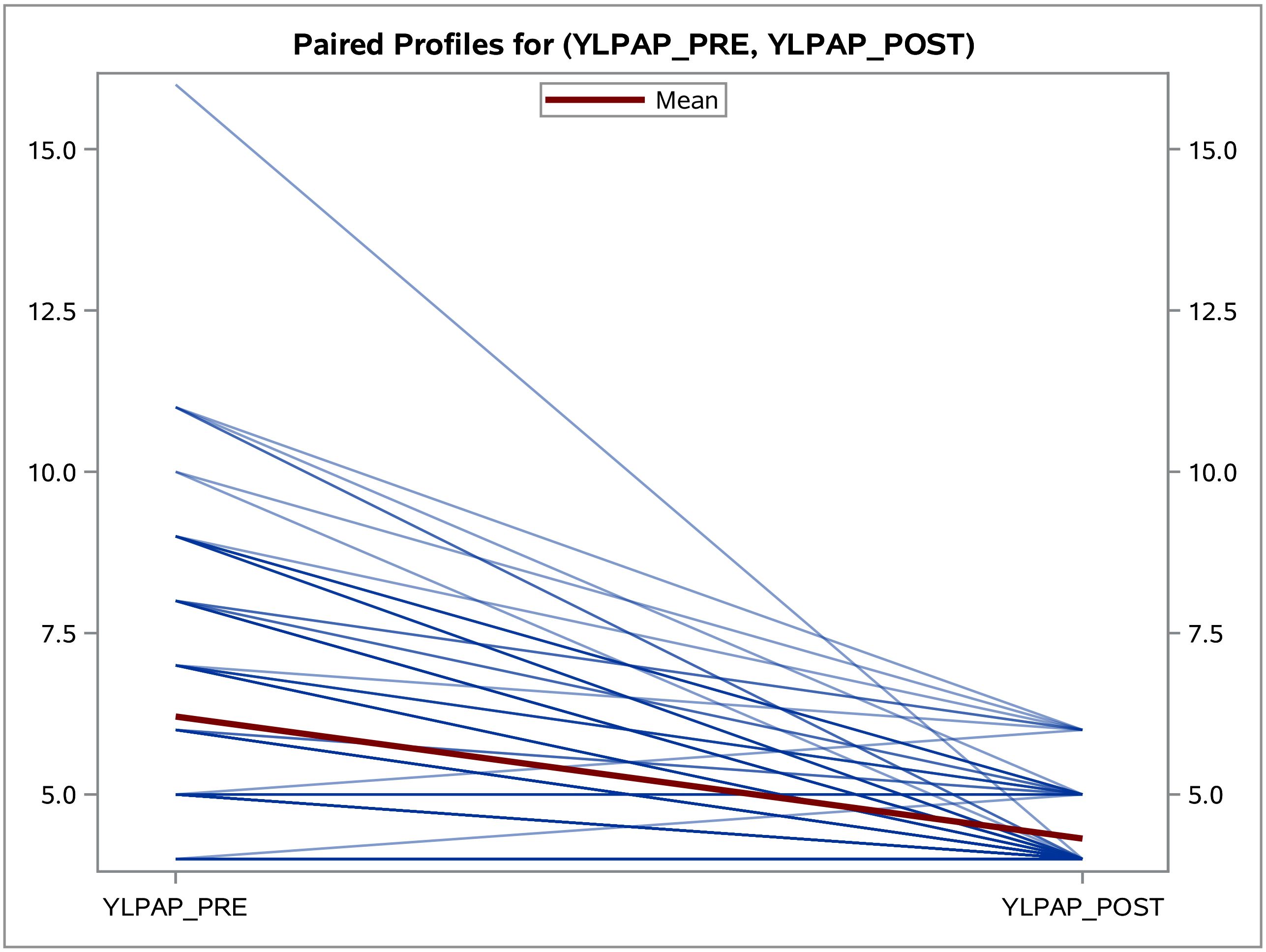

Difference: YLPAP_PRE - YLPAP_POST

Difference: YLPAP_PRE - YLPAP_POST

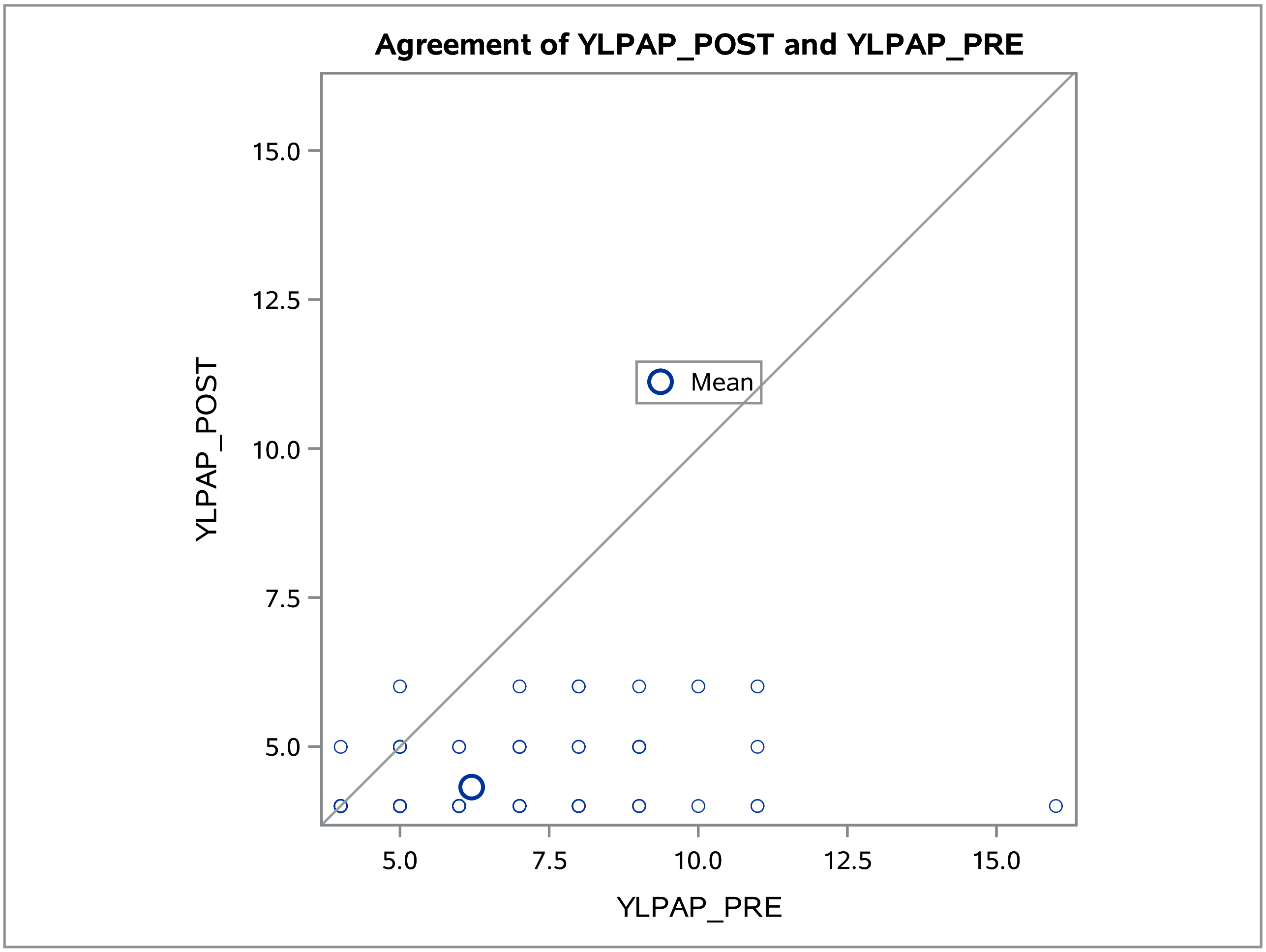

Difference: YLPAP_PRE - YLPAP_POST

Difference: YLPAP_PRE - YLPAP_POST

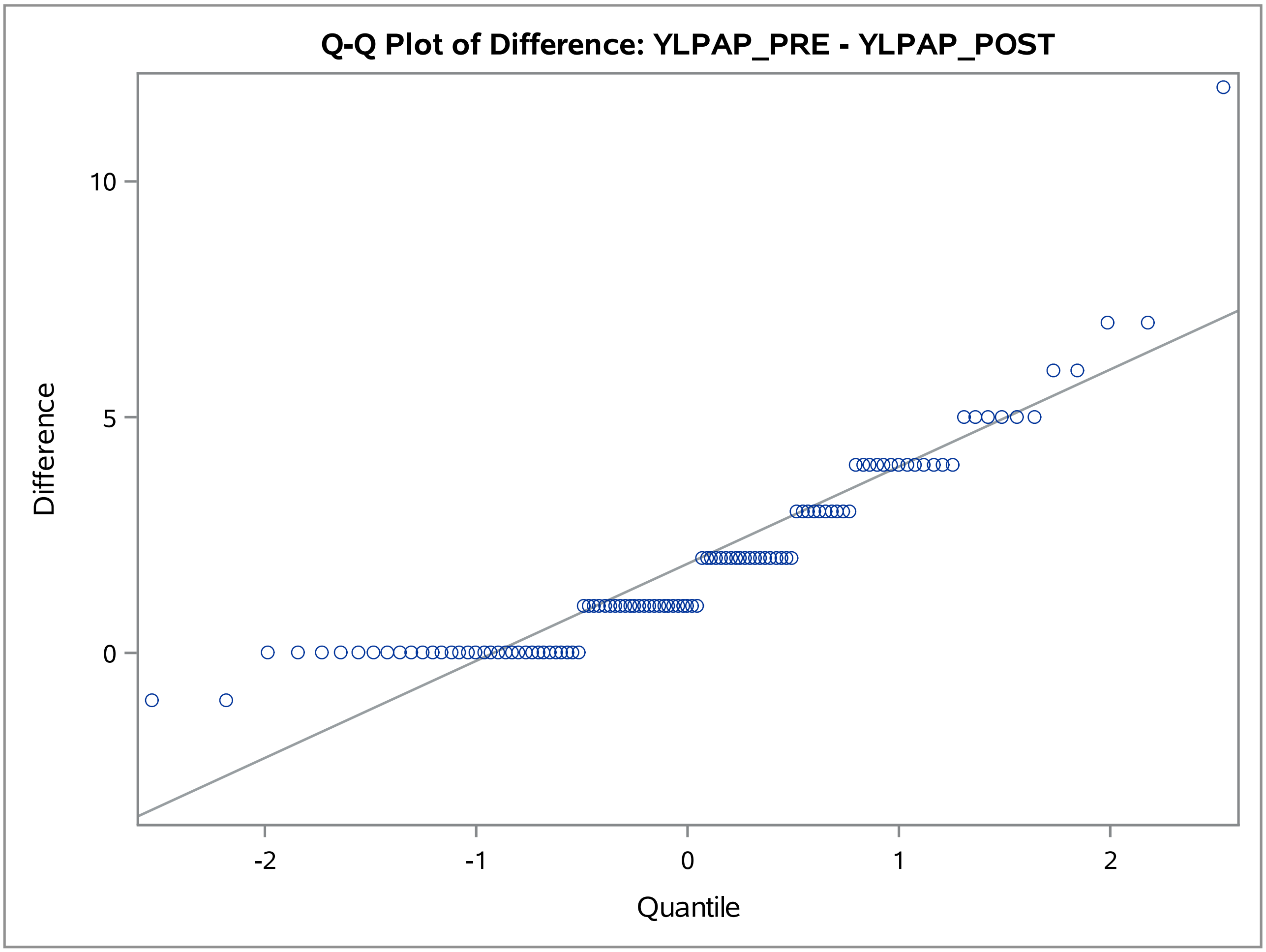

Difference: YLPDIET_PRE - YLPDIET_POST

N	Mean	Std Dev	Std Err	Minimum	Maximum
189	0.3439	4.5795	0.3331	-16.0000	25.0000

Mean	95% CL Mean		Std Dev	95% CL Std Dev	
0.3439	-0.3132	1.0010	4.5795	4.1597	5.0943

DF	t Value	Pr > |t|
188	1.03	0.3032

Difference: YLPDIET_PRE - YLPDIET_POST

Difference: YLPDIET_PRE - YLPDIET_POST

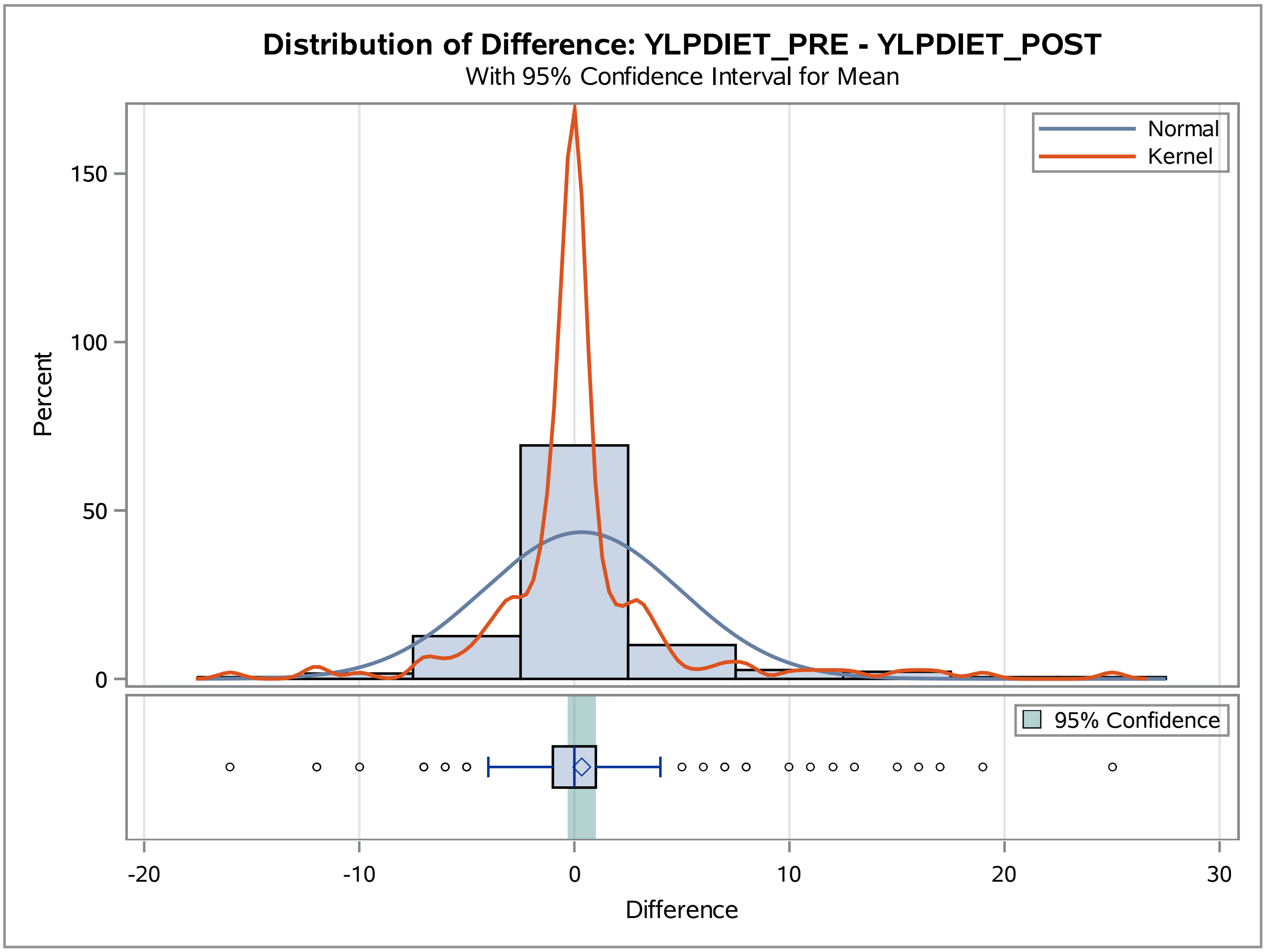

Difference: YLPDIET_PRE - YLPDIET_POST

Difference: YLPDIET_PRE - YLPDIET_POST

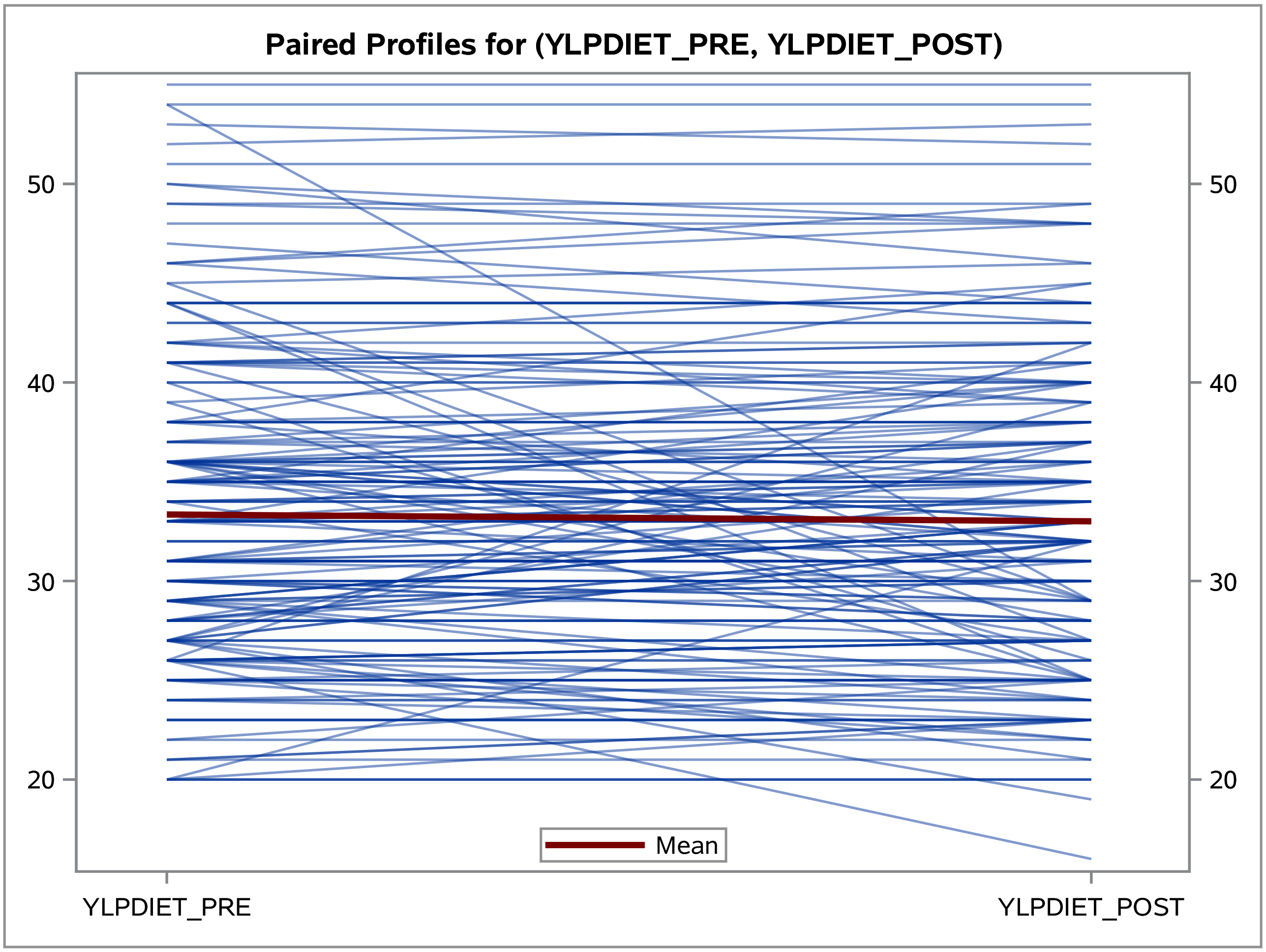

Difference: YLPDIET_PRE - YLPDIET_POST

Difference: YLPDIET_PRE - YLPDIET_POST

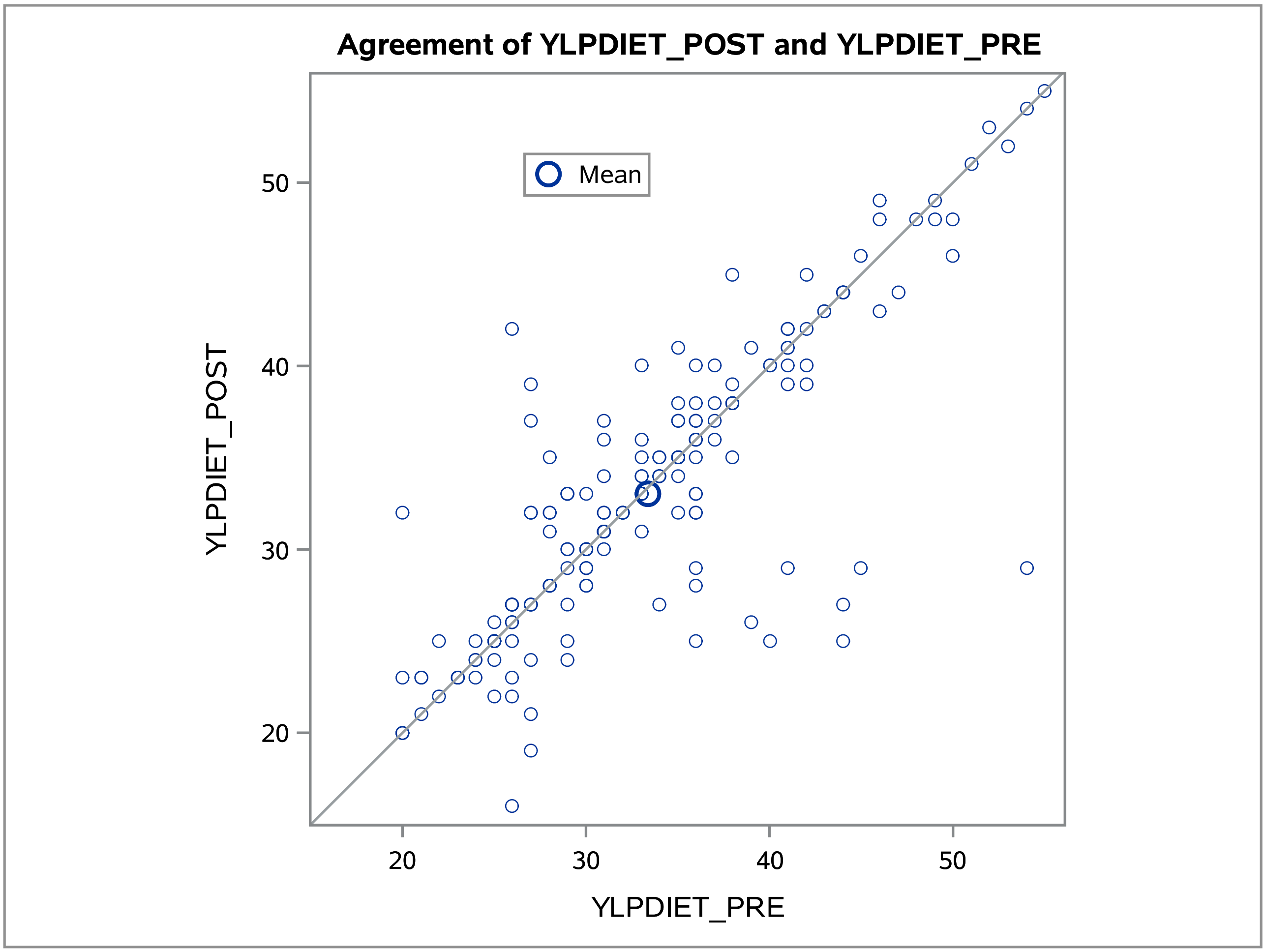

Difference: YLPDIET_PRE - YLPDIET_POST

Difference: YLPDIET_PRE - YLPDIET_POST

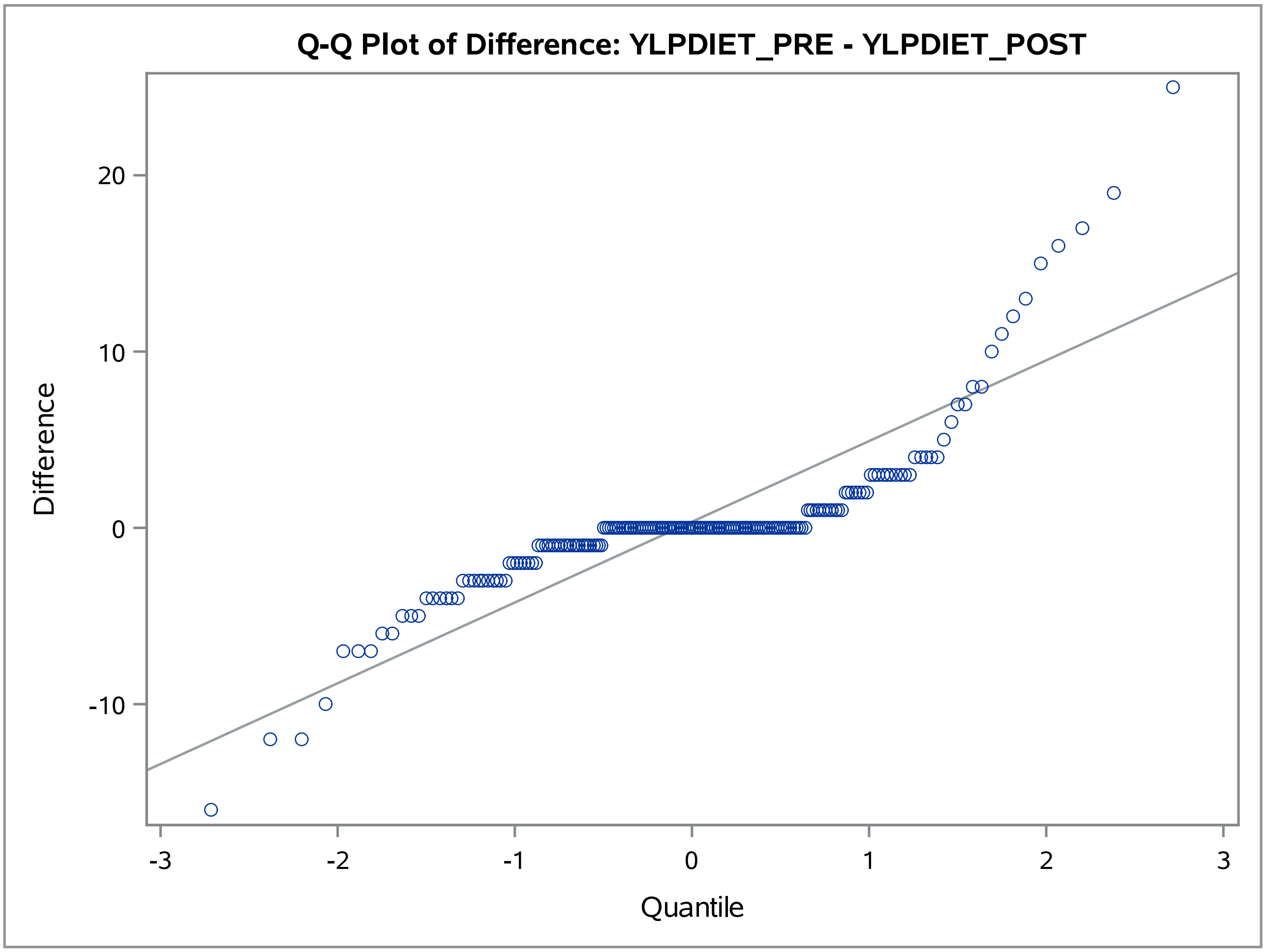

Difference: YLPDIET_PRE - YLPDIET_POST

N	Mean	Std Dev	Std Err	Minimum	Maximum
111	4.7387	5.0285	0.4773	-6.0000	25.0000

Mean	95% CL Mean		Std Dev	95% CL Std Dev	
4.7387	3.7929	5.6846	5.0285	4.4428	5.7935

DF	t Value	Pr > |t|
110	9.93	<.0001

Difference: YLPDIET_PRE - YLPDIET_POST

Difference: YLPDIET_PRE - YLPDIET_POST

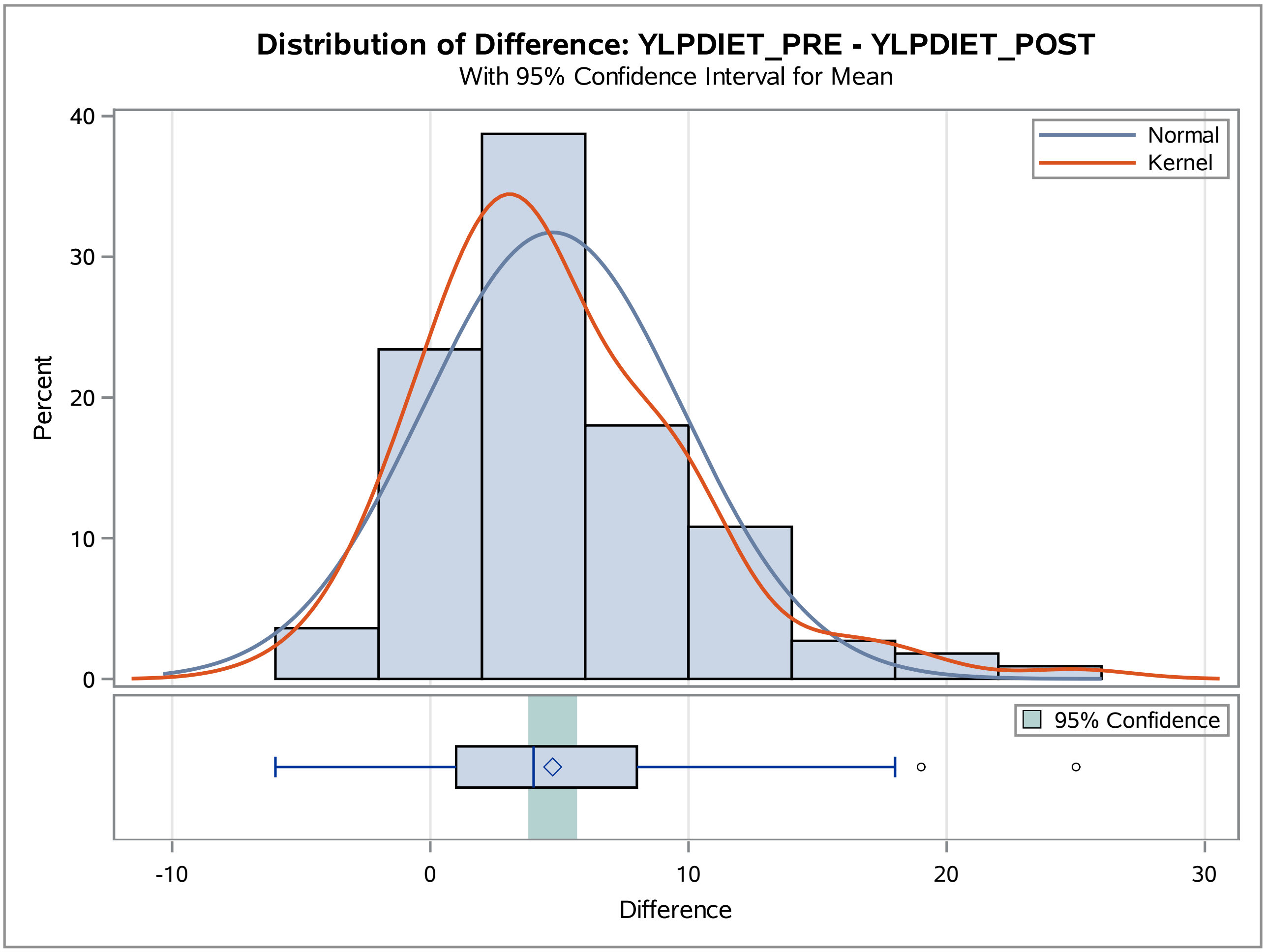

Difference: YLPDIET_PRE - YLPDIET_POST

Difference: YLPDIET_PRE - YLPDIET_POST

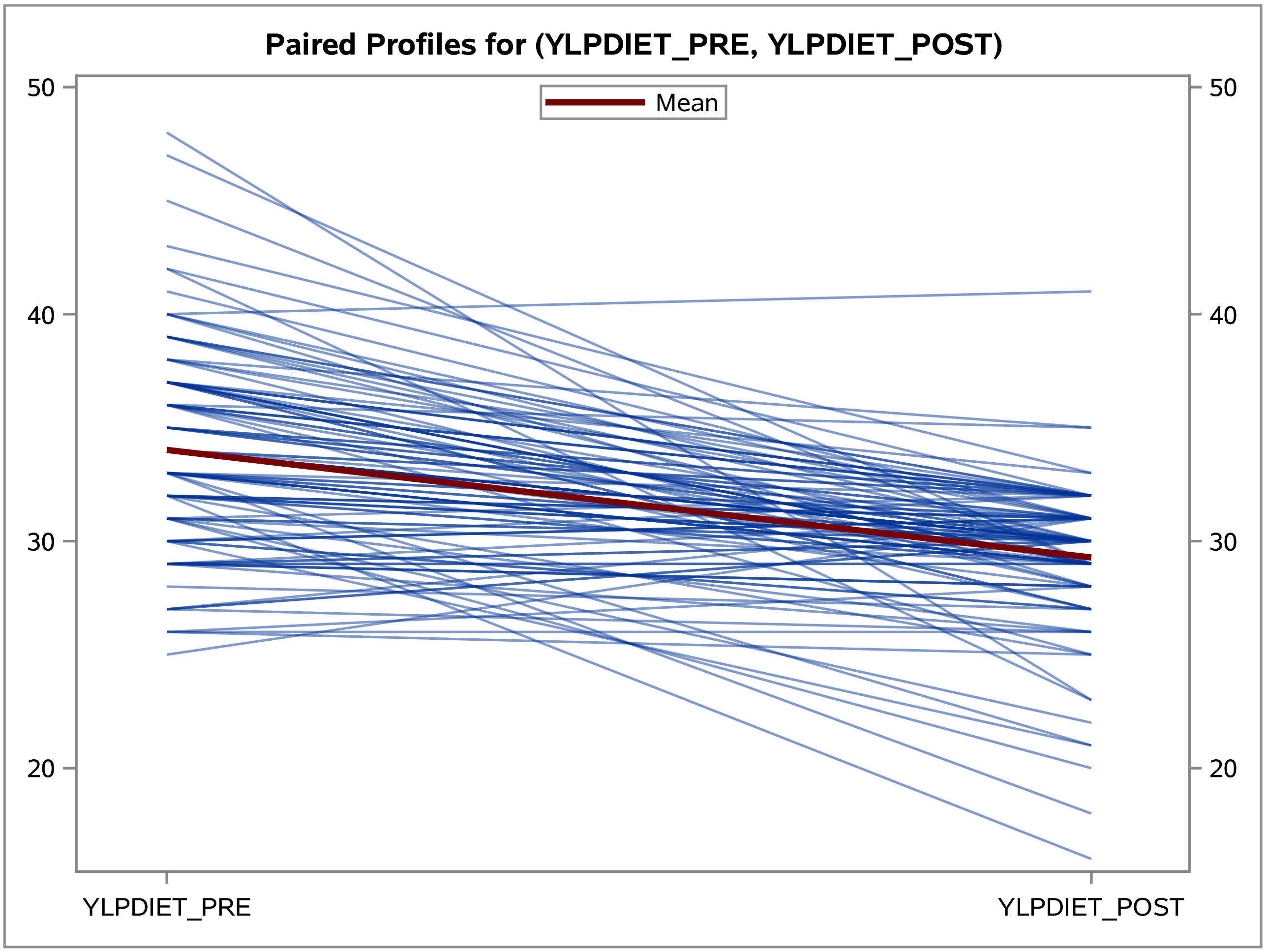

Difference: YLPDIET_PRE - YLPDIET_POST

Difference: YLPDIET_PRE - YLPDIET_POST

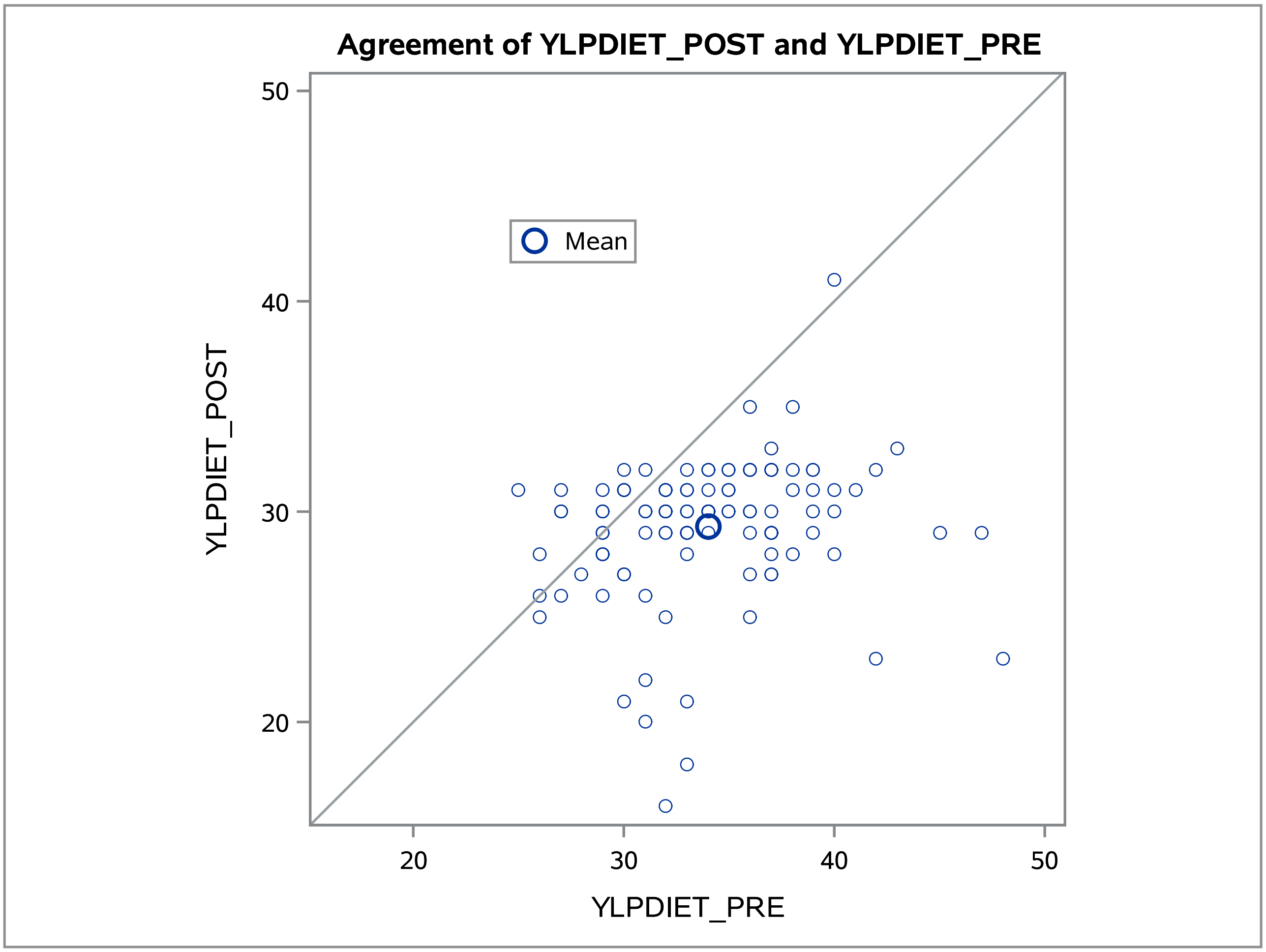

Difference: YLPDIET_PRE - YLPDIET_POST

Difference: YLPDIET_PRE - YLPDIET_POST

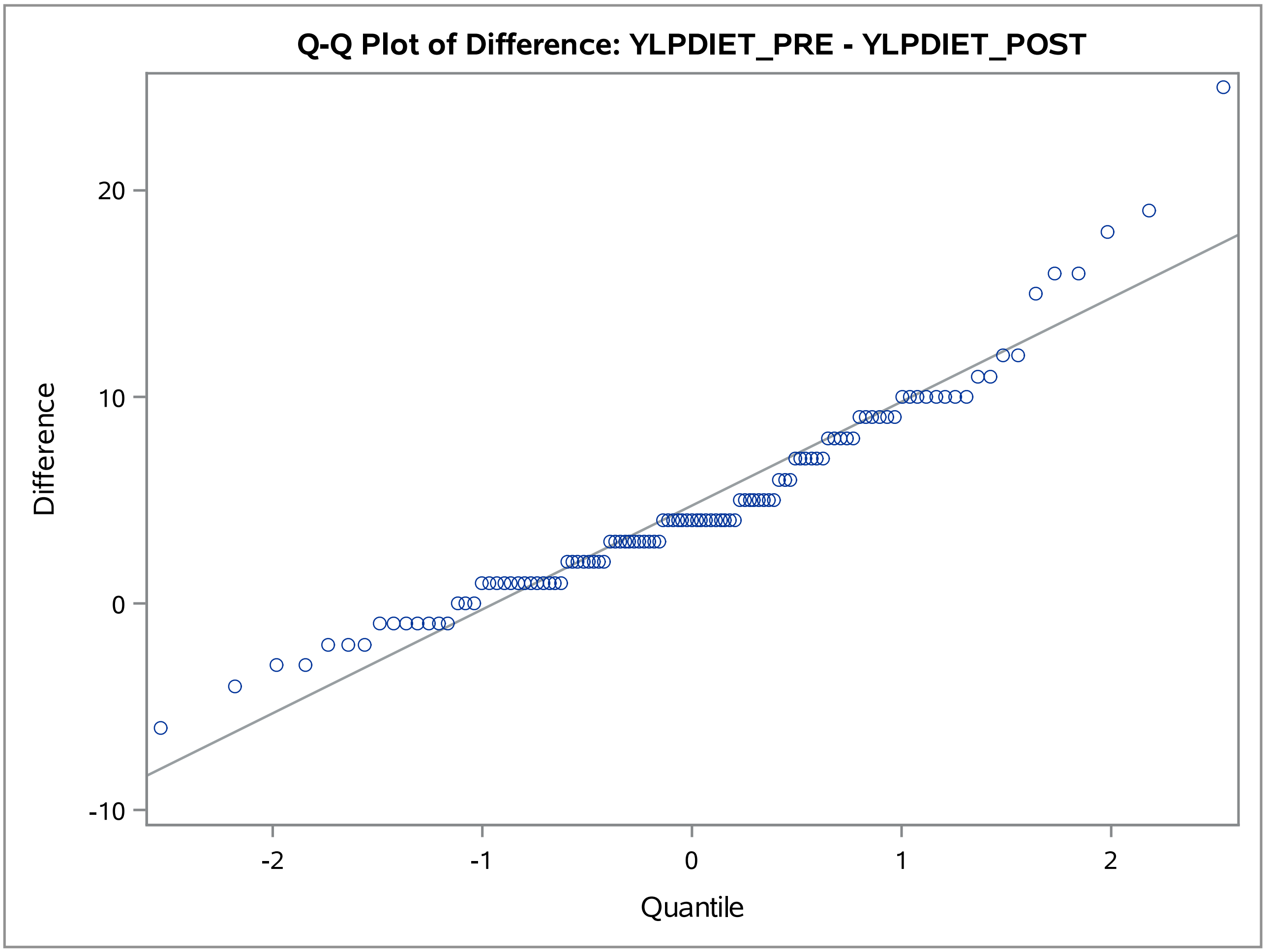

Variable: YLPPA_Diff
Group = 1

Moments			
N	189	Sum Weights	189
Mean	1.40740741	Sum Observations	266
Std Deviation	3.1737199	Variance	10.072498
Skewness	0.71839909	Kurtosis	1.9385526
Uncorrected SS	2268	Corrected SS	1893.62963
Coeff Variation	225.501151	Std Error Mean	0.23085415

Basic Statistical Measures			
Location		Variability	
Mean	1.407407	Std Deviation	3.17372
Median	0.000000	Variance	10.07250
Mode	0.000000	Range	21.00000
		Interquartile Range	3.00000

Variable: YLPPA_Diff
Group = 1

Tests for Location: Mu0=0				
**Test**	**Statistic**		**p Value**	
**Student's t**	**t**	6.096522	**Pr > |t|**	<.0001
**Sign**	**M**	32	**Pr >= |M|**	<.0001
**Signed Rank**	**S**	2018	**Pr >= |S|**	<.0001

Quantiles (Definition 5)	
**Level**	**Quantile**
**100% Max**	12
**99%**	12
**95%**	8
**90%**	6
**75% Q3**	3
**50% Median**	0
**25% Q1**	0
**10%**	-1
**5%**	-3
**1%**	-7
**0% Min**	-9

Extreme Observations			
**Lowest**		**Highest**	
**Value**	**Obs**	**Value**	**Obs**
-9	2	10	48
-7	155	10	70
-6	180	10	134
-5	87	12	117
-5	85	12	149

Variable: YLPPA_Diff
Group = 2

Moments			
**N**	111	**Sum Weights**	111
**Mean**	3.77477477	**Sum Observations**	419
**Std Deviation**	2.10490632	**Variance**	4.43063063
**Skewness**	0.9108472	**Kurtosis**	2.72407399

Variable: YLPPA_Diff
Group = 2

Moments			
Uncorrected SS	2069	Corrected SS	487.369369
Coeff Variation	55.7624348	Std Error Mean	0.19978887

Basic Statistical Measures			
Location		Variability	
Mean	3.774775	Std Deviation	2.10491
Median	4.000000	Variance	4.43063
Mode	3.000000	Range	14.00000
		Interquartile Range	3.00000

Tests for Location: Mu0=0				
Test	Statistic		p Value	
Student's t	t	18.89382	Pr > |t|	<.0001
Sign	M	53	Pr >= |M|	<.0001
Signed Rank	S	2937.5	Pr >= |S|	<.0001

Quantiles (Definition 5)	
Level	Quantile
100% Max	13
99%	9
95%	7
90%	6
75% Q3	5
50% Median	4
25% Q1	2
10%	1
5%	1
1%	0
0% Min	-1

Variable: YLPPA_Diff
Group = 2

Extreme Observations			
Lowest		Highest	
Value	Obs	Value	Obs
-1	220	8	198
0	300	8	288
0	226	9	212
0	201	9	231
1	295	13	195

Variable: YLPAP_Diff
Group = 1

Moments			
N	189	Sum Weights	189
Mean	1.70899471	Sum Observations	323
Std Deviation	2.03558072	Variance	4.14358888
Skewness	0.65760396	Kurtosis	1.05932527
Uncorrected SS	1331	Corrected SS	778.994709
Coeff Variation	119.109832	Std Error Mean	0.14806671

Basic Statistical Measures			
Location		Variability	
Mean	1.708995	Std Deviation	2.03558
Median	1.000000	Variance	4.14359
Mode	0.000000	Range	13.00000
		Interquartile Range	3.00000

Tests for Location: Mu0=0				
Test	Statistic		p Value	
Student's t	t	11.54206	Pr > |t|	<.0001
Sign	M	58	Pr >= |M|	<.0001
Signed Rank	S	4095.5	Pr >= |S|	<.0001

Variable: YLPAP_Diff
Group = 1

Quantiles (Definition 5)	
Level	Quantile
100% Max	8
99%	8
95%	5
90%	4
75% Q3	3
50% Median	1
25% Q1	0
10%	0
5%	0
1%	-3
0% Min	-5

Extreme Observations			
Lowest		Highest	
Value	Obs	Value	Obs
-5	2	7	23
-3	14	7	157
-2	97	8	34
-2	30	8	70
-1	185	8	71

Variable: YLPAP_Diff
Group = 2

Moments			
N	111	Sum Weights	111
Mean	1.89189189	Sum Observations	210
Std Deviation	2.05979413	Variance	4.24275184
Skewness	1.55881145	Kurtosis	4.25919186
Uncorrected SS	864	Corrected SS	466.702703
Coeff Variation	108.874832	Std Error Mean	0.19550701

Variable: YLPAP_Diff
Group = 2

Basic Statistical Measures			
Location		Variability	
Mean	1.891892	Std Deviation	2.05979
Median	1.000000	Variance	4.24275
Mode	0.000000	Range	13.00000
		Interquartile Range	3.00000

Tests for Location: Mu0=0				
Test	Statistic		p Value	
Student's t	t	9.67685	Pr > |t|	<.0001
Sign	M	37.5	Pr >= |M|	<.0001
Signed Rank	S	1553	Pr >= |S|	<.0001

Quantiles (Definition 5)	
Level	Quantile
100% Max	12
99%	7
95%	5
90%	4
75% Q3	3
50% Median	1
25% Q1	0
10%	0
5%	0
1%	-1
0% Min	-1

Extreme Observations			
Lowest		Highest	
Value	Obs	Value	Obs
-1	281	6	193
-1	269	6	235
0	300	7	195
0	299	7	230
0	298	12	192

Variable: YLPDIET_Diff
Group = 1

Moments			
N	189	Sum Weights	189
Mean	0.34391534	Sum Observations	65
Std Deviation	4.57946707	Variance	20.9715186
Skewness	1.60262288	Kurtosis	8.19798137
Uncorrected SS	3965	Corrected SS	3942.6455
Coeff Variation	1331.56812	Std Error Mean	0.33310721

Basic Statistical Measures			
Location		Variability	
Mean	0.343915	Std Deviation	4.57947
Median	0.000000	Variance	20.97152
Mode	0.000000	Range	41.00000
		Interquartile Range	2.00000

Tests for Location: Mu0=0				
Test	Statistic		p Value	
Student's t	t	1.032446	Pr > |t|	0.3032
Sign	M	-4.5	Pr >= |M|	0.4394
Signed Rank	S	36.5	Pr >= |S|	0.9098

Quantiles (Definition 5)	
Level	Quantile
100% Max	25
99%	19
95%	8
90%	4
75% Q3	1
50% Median	0
25% Q1	-1
10%	-3
5%	-5
1%	-12
0% Min	-16

Variable: YLPDIET_Diff
Group = 1

Extreme Observations			
Lowest		Highest	
Value	Obs	Value	Obs
-16	134	15	178
-12	180	16	149
-12	179	17	58
-10	87	19	34
-7	189	25	165

Variable: YLPDIET_Diff
Group = 2

Moments			
N	111	Sum Weights	111
Mean	4.73873874	Sum Observations	526
Std Deviation	5.02848561	Variance	25.2856675
Skewness	1.04271575	Kurtosis	2.11536323
Uncorrected SS	5274	Corrected SS	2781.42342
Coeff Variation	106.11443	Std Error Mean	0.47728273

Basic Statistical Measures			
Location		Variability	
Mean	4.738739	Std Deviation	5.02849
Median	4.000000	Variance	25.28567
Mode	4.000000	Range	31.00000
		Interquartile Range	7.00000

Tests for Location: Mu0=0				
Test	Statistic		p Value	
Student's t	t	9.928578	Pr > |t|	<.0001
Sign	M	40	Pr >= |M|	<.0001
Signed Rank	S	2591	Pr >= |S|	<.0001

Variable: YLPDIET_Diff
Group = 2

Quantiles (Definition 5)	
Level	Quantile
100% Max	25
99%	19
95%	15
90%	10
75% Q3	8
50% Median	4
25% Q1	1
10%	-1
5%	-2
1%	-4
0% Min	-6

Extreme Observations			
Lowest		Highest	
Value	Obs	Value	Obs
-6	276	16	217
-4	274	16	235
-3	297	18	195
-3	267	19	225
-2	300	25	222

Variable: Age

Group	N	Mean	Std Dev	Std Err	Minimum	Maximum
1	189	61.4127	5.8043	0.4222	55.0000	78.0000
2	111	68.6306	7.8728	0.7473	54.0000	86.0000
Diff (1-2)		-7.2179	6.6433	0.7944		

Group	Method	Mean	95% CL Mean		Std Dev	95% CL Std Dev	
1		61.4127	60.5798	62.2456	5.8043	5.2723	6.4568
2		68.6306	67.1498	70.1115	7.8728	6.9557	9.0705
Diff (1-2)	Pooled	-7.2179	-8.7813	-5.6545	6.6433	6.1501	7.2232
Diff (1-2)	Satterthwaite	-7.2179	-8.9115	-5.5244			

Variable: Age
Group = 2

Variable: Age

Method	Variances	DF	t Value	Pr > |t|
**Pooled**	Equal	298	-9.09	<.0001
**Satterthwaite**	Unequal	180.67	-8.41	<.0001

Equality of Variances				
**Method**	**Num DF**	**Den DF**	**F Value**	**Pr > F**
**Folded F**	110	188	1.84	0.0002

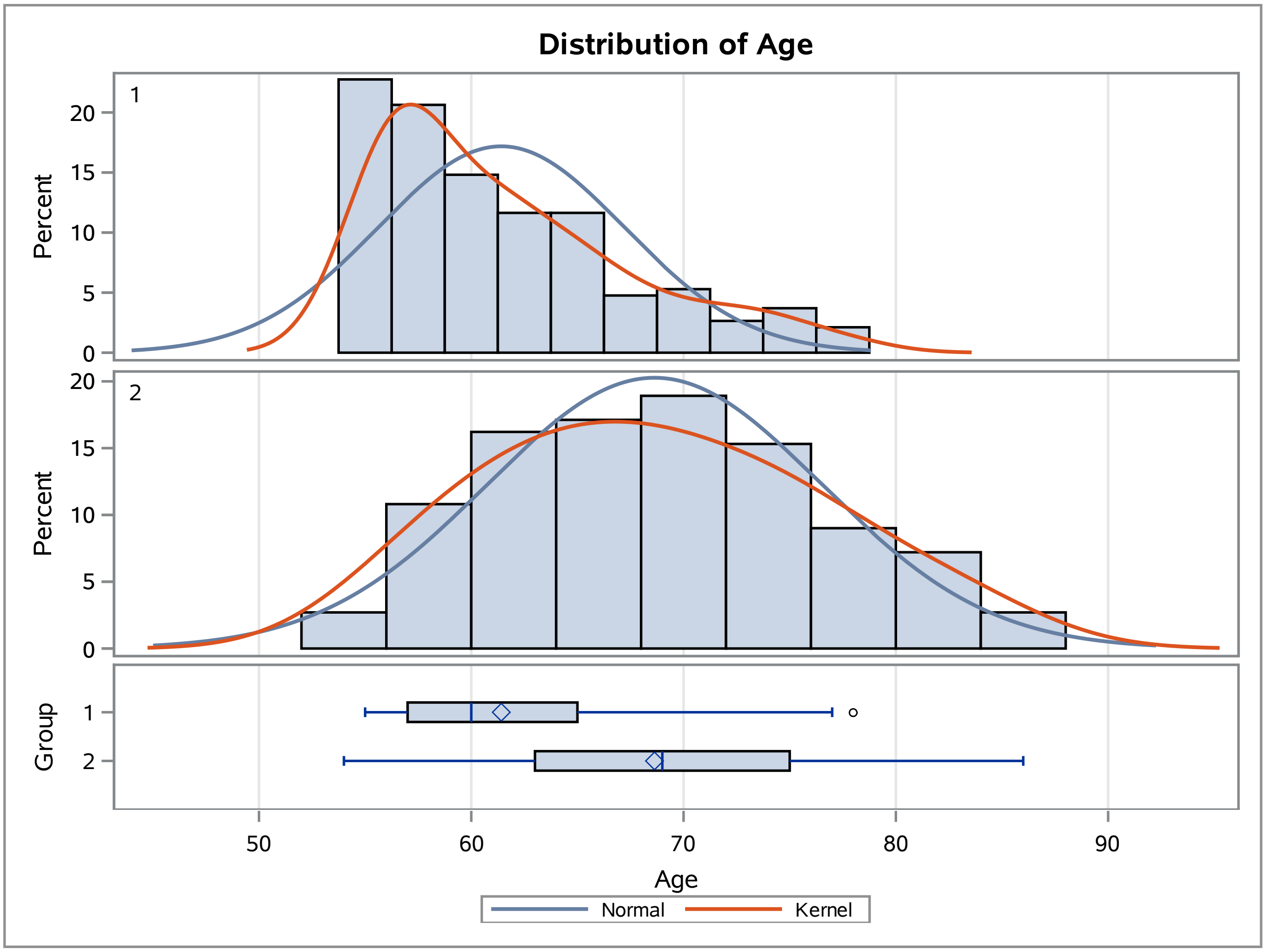

Variable: Age
Group = 2

Variable: Age

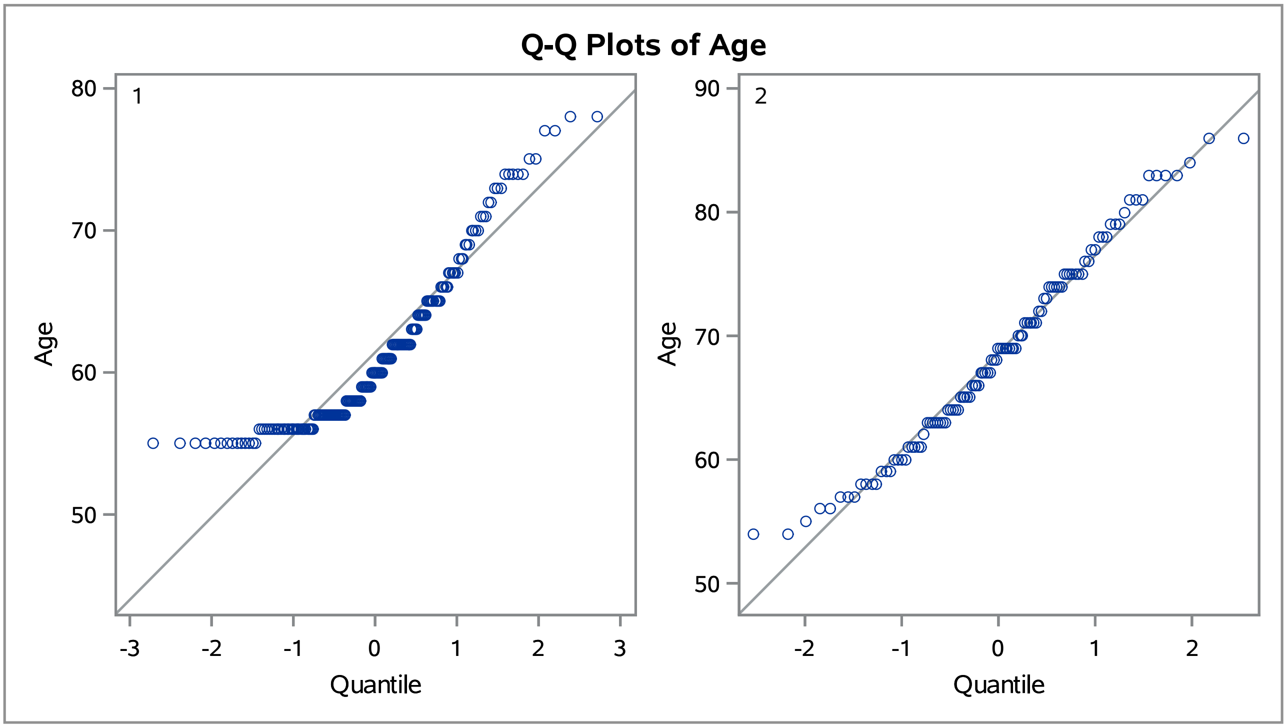

Variable: YLPPA_Diff

Group	N	Mean	Std Dev	Std Err	Minimum	Maximum
**1**	189	1.4074	3.1737	0.2309	-9.0000	12.0000
**2**	111	3.7748	2.1049	0.1998	-1.0000	13.0000
**Diff (1-2)**		-2.3674	2.8266	0.3380		

Group	Method	Mean	95% CL Mean		Std Dev	95% CL Std Dev	
**1**		1.4074	0.9520	1.8628	3.1737	2.8828	3.5305
**2**		3.7748	3.3788	4.1707	2.1049	1.8597	2.4251
**Diff (1-2)**	**Pooled**	-2.3674	-3.0326	-1.7022	2.8266	2.6168	3.0734
**Diff (1-2)**	**Satterthwaite**	-2.3674	-2.9682	-1.7665			

Method	Variances	DF	t Value	Pr > |t|
**Pooled**	Equal	298	-7.00	<.0001
**Satterthwaite**	Unequal	293.6	-7.75	<.0001

Equality of Variances				
**Method**	**Num DF**	**Den DF**	**F Value**	**Pr > F**
**Folded F**	188	110	2.27	<.0001

Variable: YLPPA_Diff
Group = 2

Variable: YLPPA_Diff

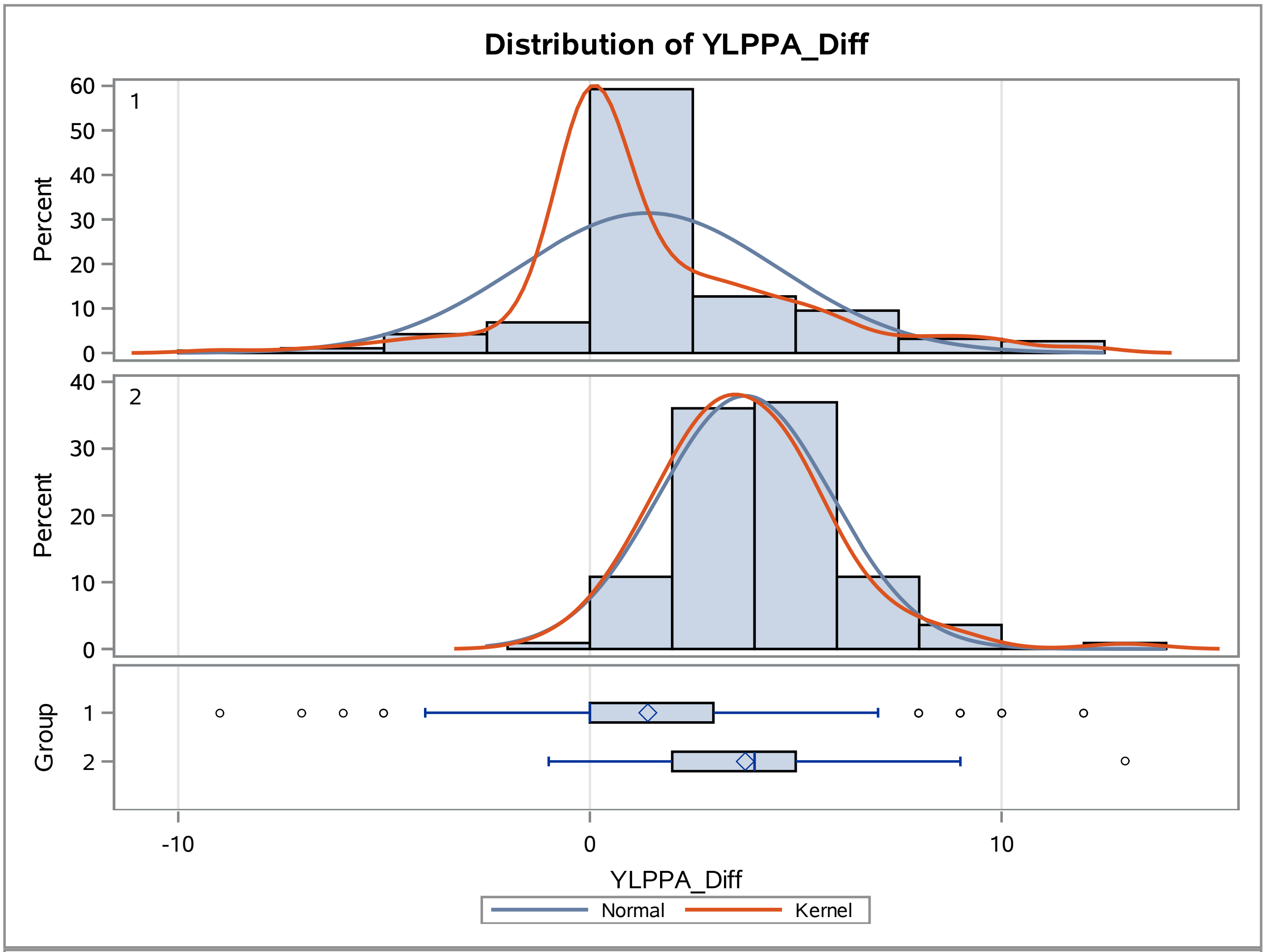

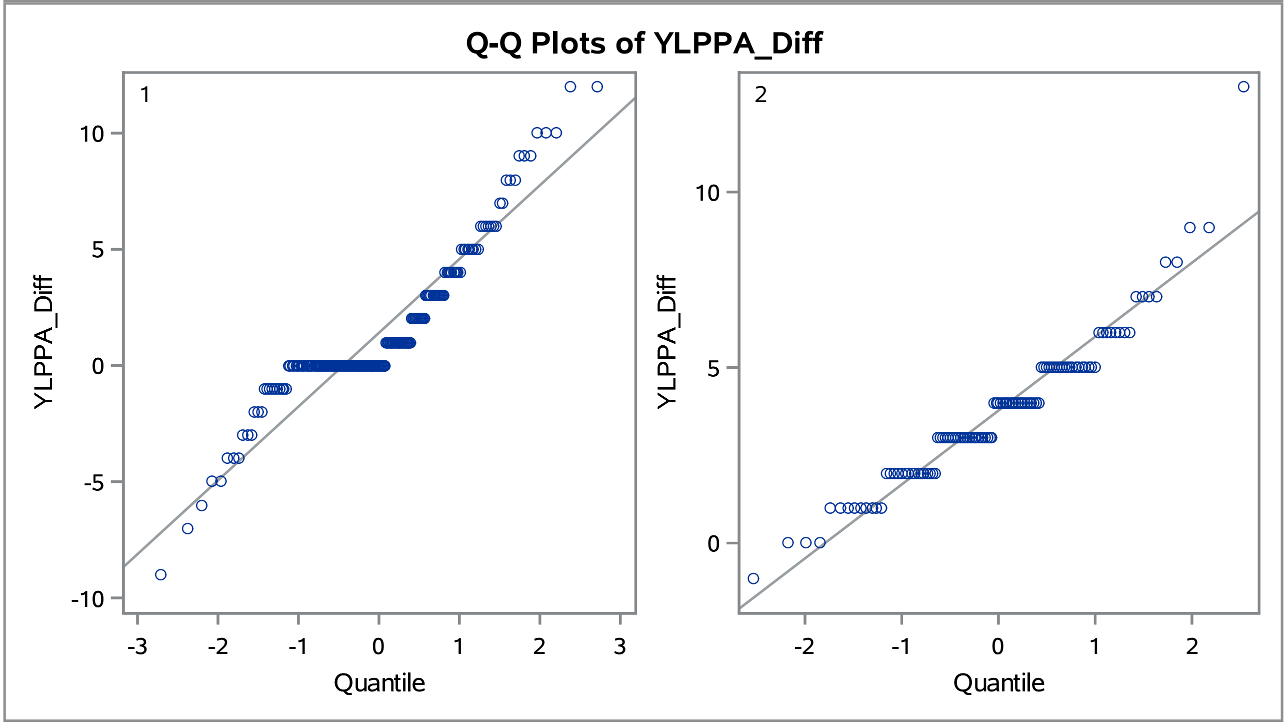

Variable: YLPAP_Diff
Group = 2

Variable: YLPAP_Diff

Variable: YLPAP_Diff

Group	N	Mean	Std Dev	Std Err	Minimum	Maximum
1	189	1.7090	2.0356	0.1481	-5.0000	8.0000
2	111	1.8919	2.0598	0.1955	-1.0000	12.0000
Diff (1-2)		-0.1829	2.0446	0.2445		

Group	Method	Mean	95% CL Mean		Std Dev	95% CL Std Dev	
1		1.7090	1.4169	2.0011	2.0356	1.8490	2.2644
2		1.8919	1.5044	2.2793	2.0598	1.8199	2.3732
Diff (1-2)	Pooled	-0.1829	-0.6640	0.2983	2.0446	1.8928	2.2230
Diff (1-2)	Satterthwaite	-0.1829	-0.6661	0.3003			

Method	Variances	DF	t Value	Pr > |t|
Pooled	Equal	298	-0.75	0.4550
Satterthwaite	Unequal	228.41	-0.75	0.4566

Equality of Variances				
Method	Num DF	Den DF	F Value	Pr > F
Folded F	110	188	1.02	0.8776

Variable: YLPAP_Diff
Group = 2

Variable: YLPAP_Diff

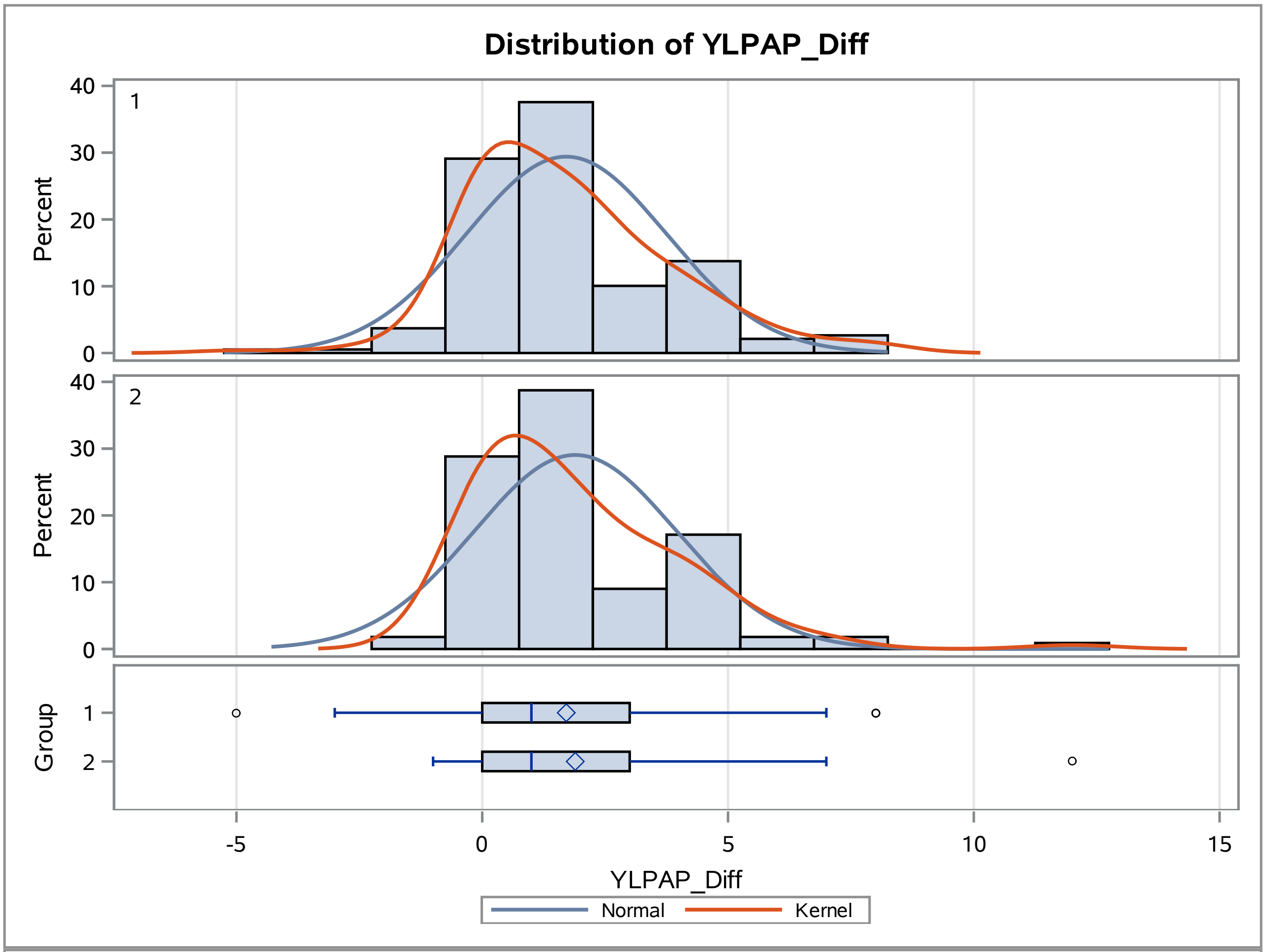

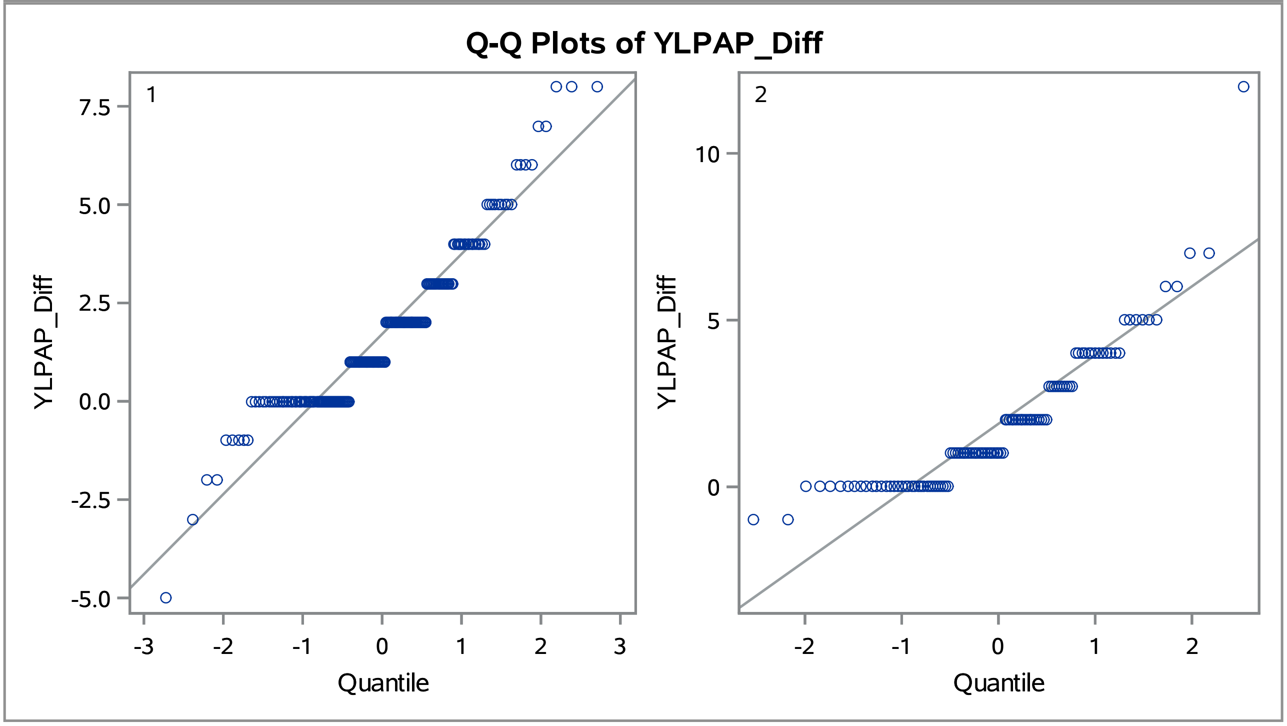

Variable: YLPDIET_Diff
Group = 2

Variable: YLPDIET_Diff

Variable: YLPDIET_Diff

Group	N	Mean	Std Dev	Std Err	Minimum	Maximum
1	189	0.3439	4.5795	0.3331	-16.0000	25.0000
2	111	4.7387	5.0285	0.4773	-6.0000	25.0000
Diff (1-2)		-4.3948	4.7502	0.5680		

Group	Method	Mean	95% CL Mean		Std Dev	95% CL Std Dev	
1		0.3439	-0.3132	1.0010	4.5795	4.1597	5.0943
2		4.7387	3.7929	5.6846	5.0285	4.4428	5.7935
Diff (1-2)	Pooled	-4.3948	-5.5127	-3.2770	4.7502	4.3975	5.1648
Diff (1-2)	Satterthwaite	-4.3948	-5.5421	-3.2476			

Method	Variances	DF	t Value	Pr > |t|
Pooled	Equal	298	-7.74	<.0001
Satterthwaite	Unequal	213.61	-7.55	<.0001

Equality of Variances				
Method	Num DF	Den DF	F Value	Pr > F
Folded F	110	188	1.21	0.2614

Variable: YLPDIET_Diff
Group = 2

Variable: YLPDIET_Diff

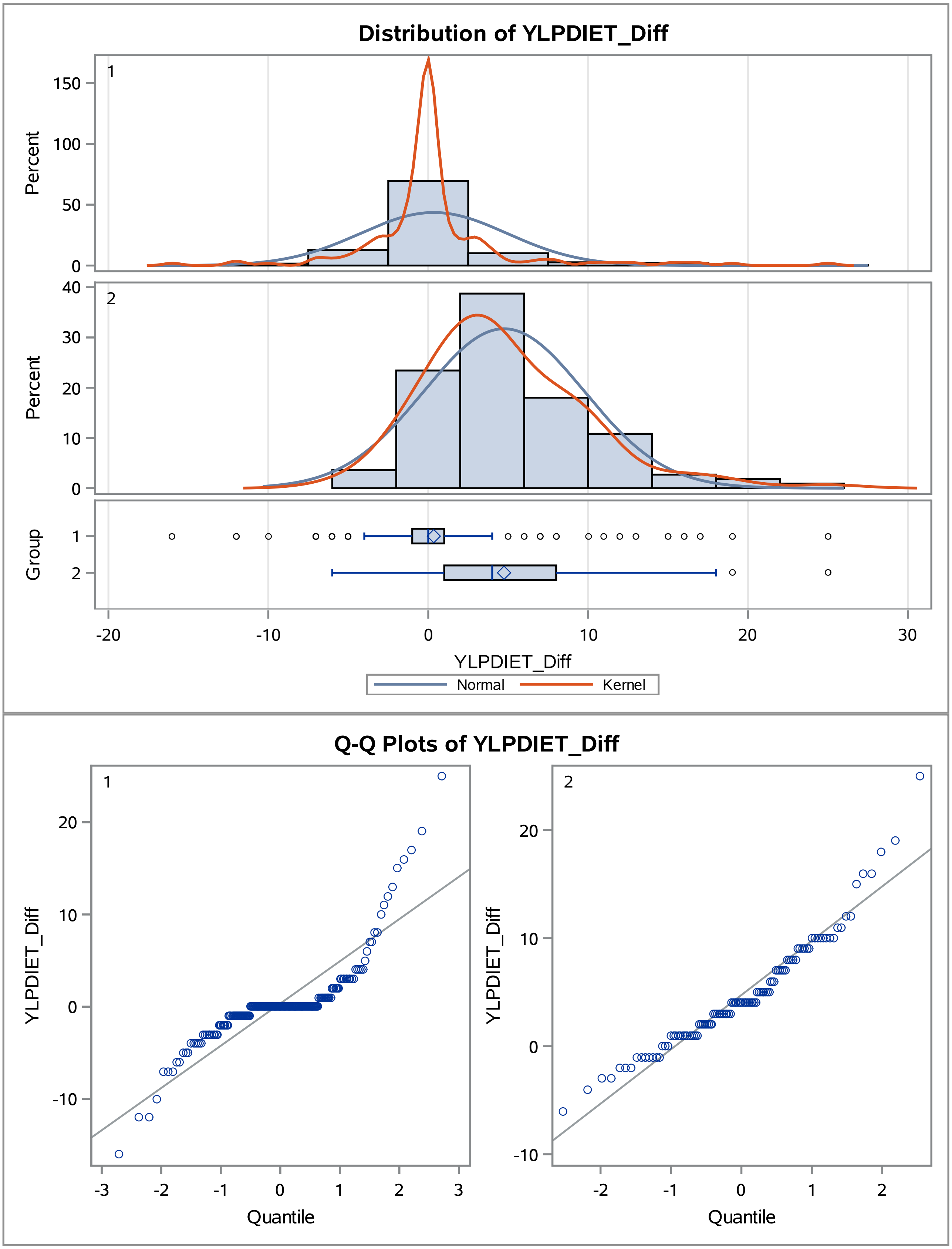

Variable: VASD
Group = 2

Variable: VASD

Variable: VASD

Group	N	Mean	Std Dev	Std Err	Minimum	Maximum
**1**	189	4.3757	2.3883	0.1737	1.0000	10.0000
**2**	111	4.8739	2.1282	0.2020	0	10.0000
**Diff (1-2)**		-0.4982	2.2957	0.2745		

Group	Method	Mean	95% CL Mean		Std Dev	95% CL Std Dev	
**1**		4.3757	4.0330	4.7184	2.3883	2.1693	2.6568
**2**		4.8739	4.4736	5.2742	2.1282	1.8803	2.4520
**Diff (1-2)**	**Pooled**	-0.4982	-1.0385	0.0420	2.2957	2.1253	2.4961
**Diff (1-2)**	**Satterthwaite**	-0.4982	-1.0229	0.0265			

Method	Variances	DF	t Value	Pr > |t|
**Pooled**	Equal	298	-1.81	0.0706
**Satterthwaite**	Unequal	252.17	-1.87	0.0626

Equality of Variances				
**Method**	**Num DF**	**Den DF**	**F Value**	**Pr > F**
**Folded F**	188	110	1.26	0.1848

Variable: VASD
Group = 2

Variable: VASD

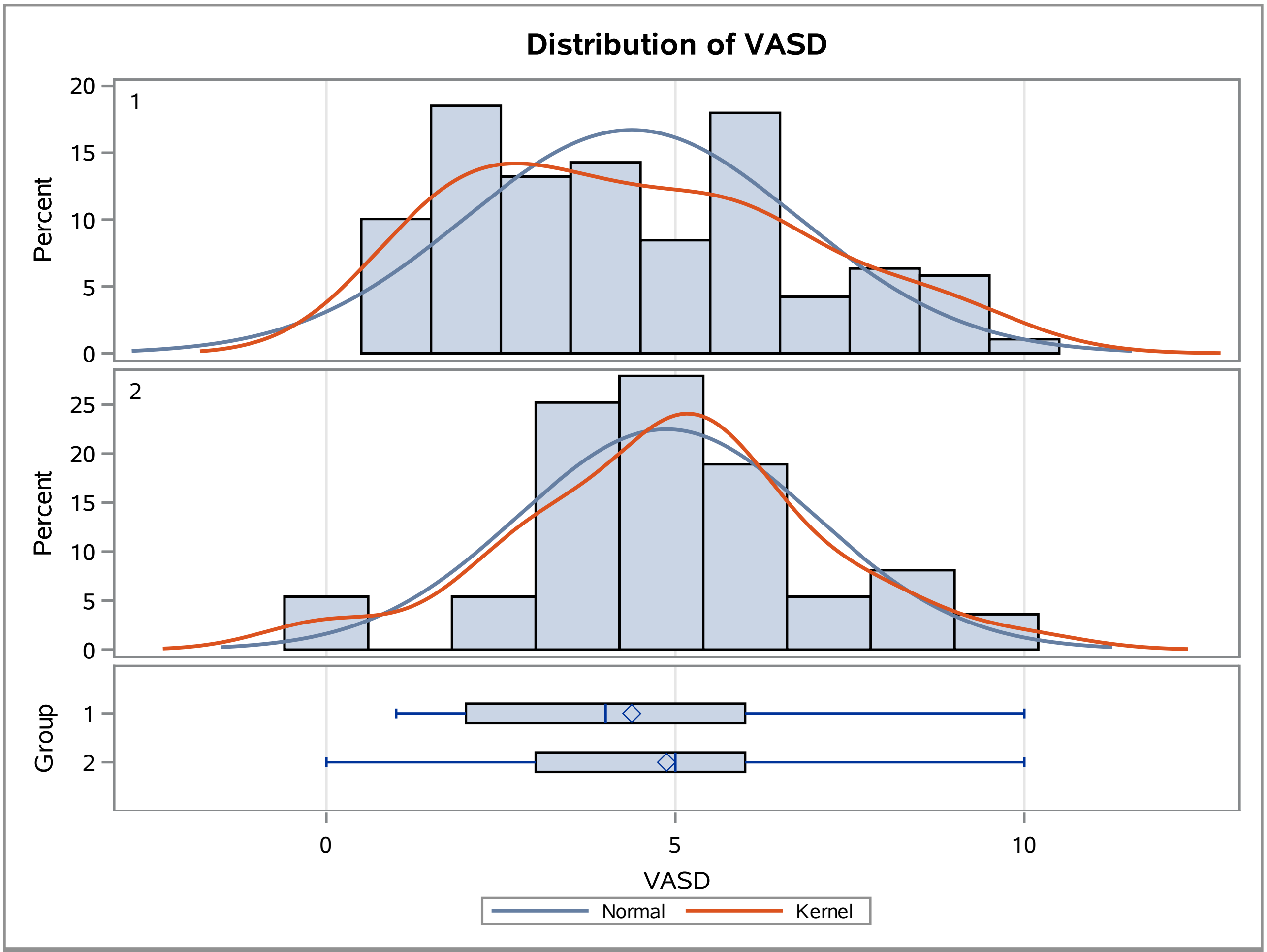

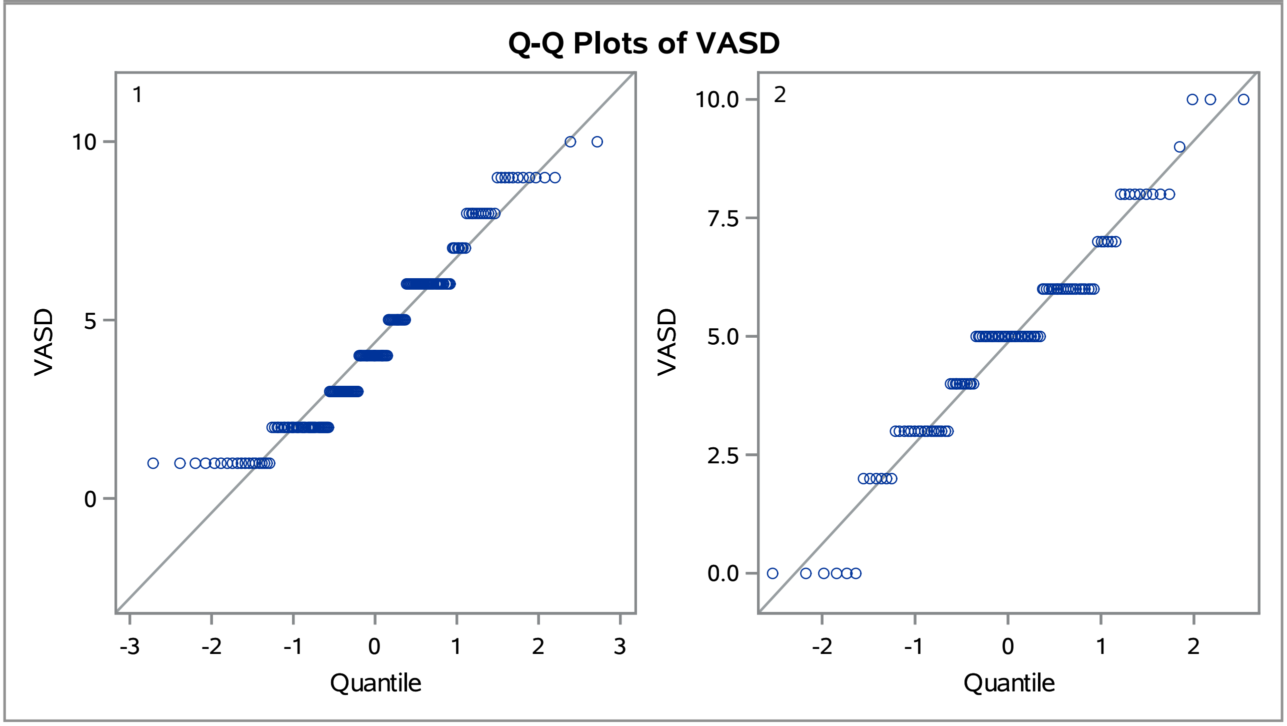

Variable: PHQ
Group = 2

Variable: PHQ

Variable: PHQ

Group	N	Mean	Std Dev	Std Err	Minimum	Maximum
**1**	189	4.1376	4.0975	0.2980	0	20.0000
**2**	111	5.1081	4.0031	0.3800	0	19.0000
**Diff (1-2)**		-0.9705	4.0629	0.4859		

Group	Method	Mean	95% CL Mean		Std Dev	95% CL Std Dev	
**1**		4.1376	3.5496	4.7255	4.0975	3.7219	4.5581
**2**		5.1081	4.3551	5.8611	4.0031	3.5368	4.6121
**Diff (1-2)**	**Pooled**	-0.9705	-1.9267	-0.0144	4.0629	3.7613	4.4175
**Diff (1-2)**	**Satterthwaite**	-0.9705	-1.9219	-0.0192			

Method	Variances	DF	t Value	Pr > |t|
**Pooled**	Equal	298	-2.00	0.0467
**Satterthwaite**	Unequal	234.97	-2.01	0.0456

Equality of Variances				
**Method**	**Num DF**	**Den DF**	**F Value**	**Pr > F**
**Folded F**	188	110	1.05	0.7958

Variable: PHQ
Group = 2

Variable: PHQ

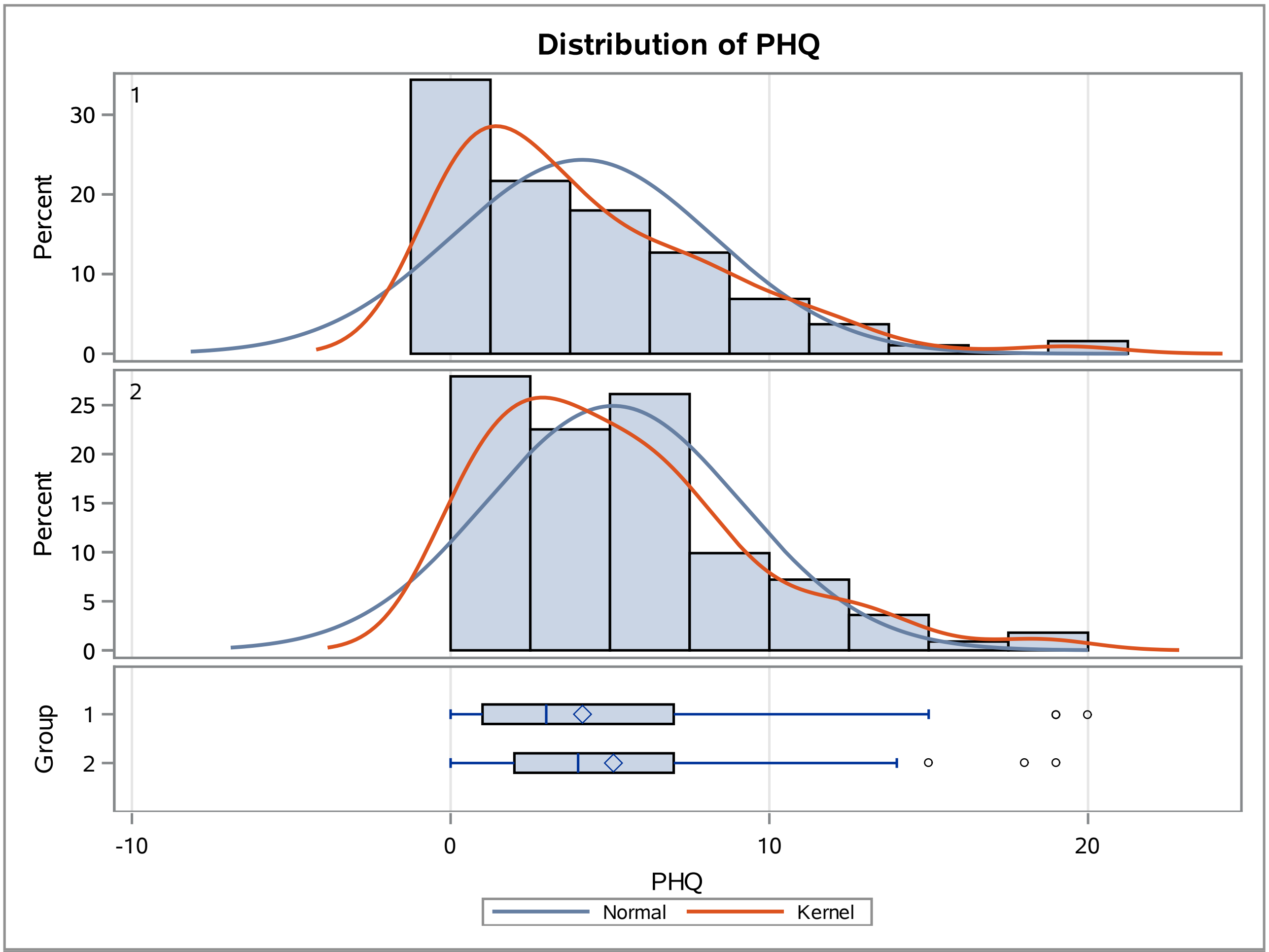

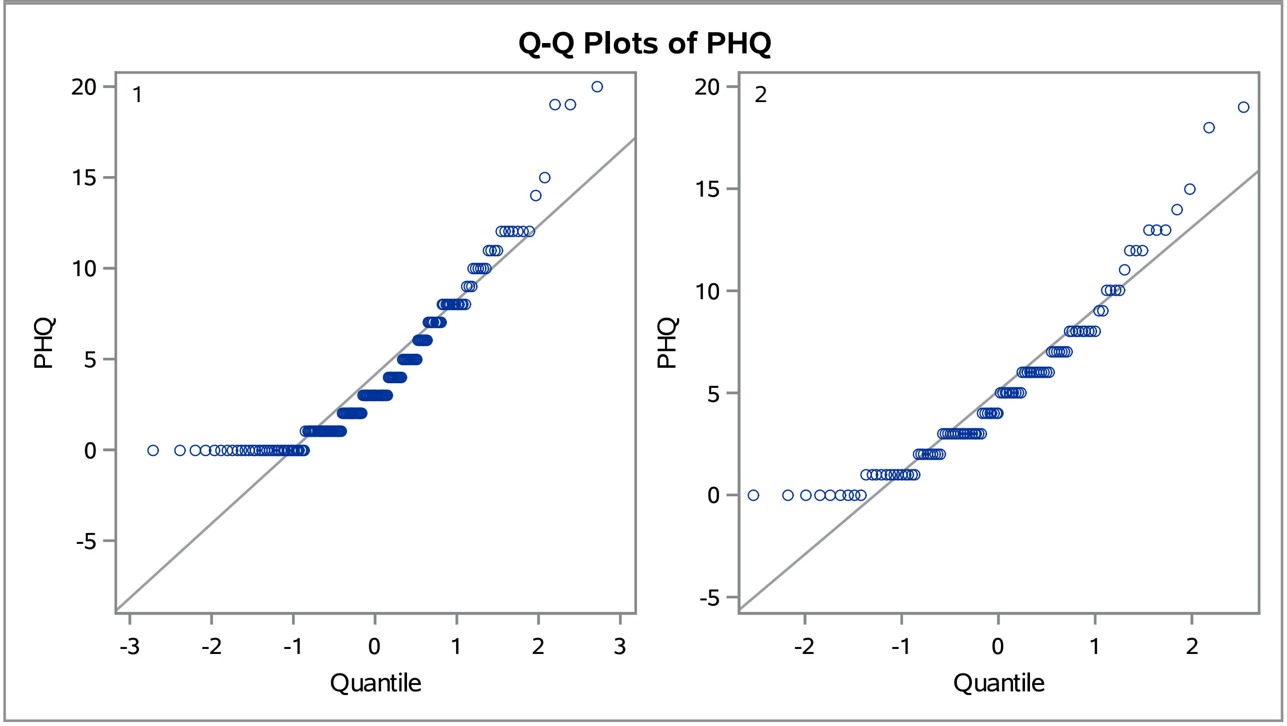

Variable: VASA
Group = 2

Variable: VASA

Variable: VASA

Group	N	Mean	Std Dev	Std Err	Minimum	Maximum
**1**	189	5.2063	2.0277	0.1475	1.0000	9.0000
**2**	111	5.2973	2.6236	0.2490	0	10.0000
**Diff (1-2)**		-0.0909	2.2660	0.2710		

Group	Method	Mean	95% CL Mean		Std Dev	95% CL Std Dev	
**1**		5.2063	4.9154	5.4973	2.0277	1.8418	2.2556
**2**		5.2973	4.8038	5.7908	2.6236	2.3180	3.0228
**Diff (1-2)**	**Pooled**	-0.0909	-0.6242	0.4423	2.2660	2.0978	2.4638
**Diff (1-2)**	**Satterthwaite**	-0.0909	-0.6619	0.4800			

Method	Variances	DF	t Value	Pr > |t|
**Pooled**	Equal	298	-0.34	0.7374
**Satterthwaite**	Unequal	187.23	-0.31	0.7537

Equality of Variances				
Method	Num DF	Den DF	F Value	Pr > F
**Folded F**	110	188	1.67	0.0019

Variable: VASA
Group = 2

Variable: VASA

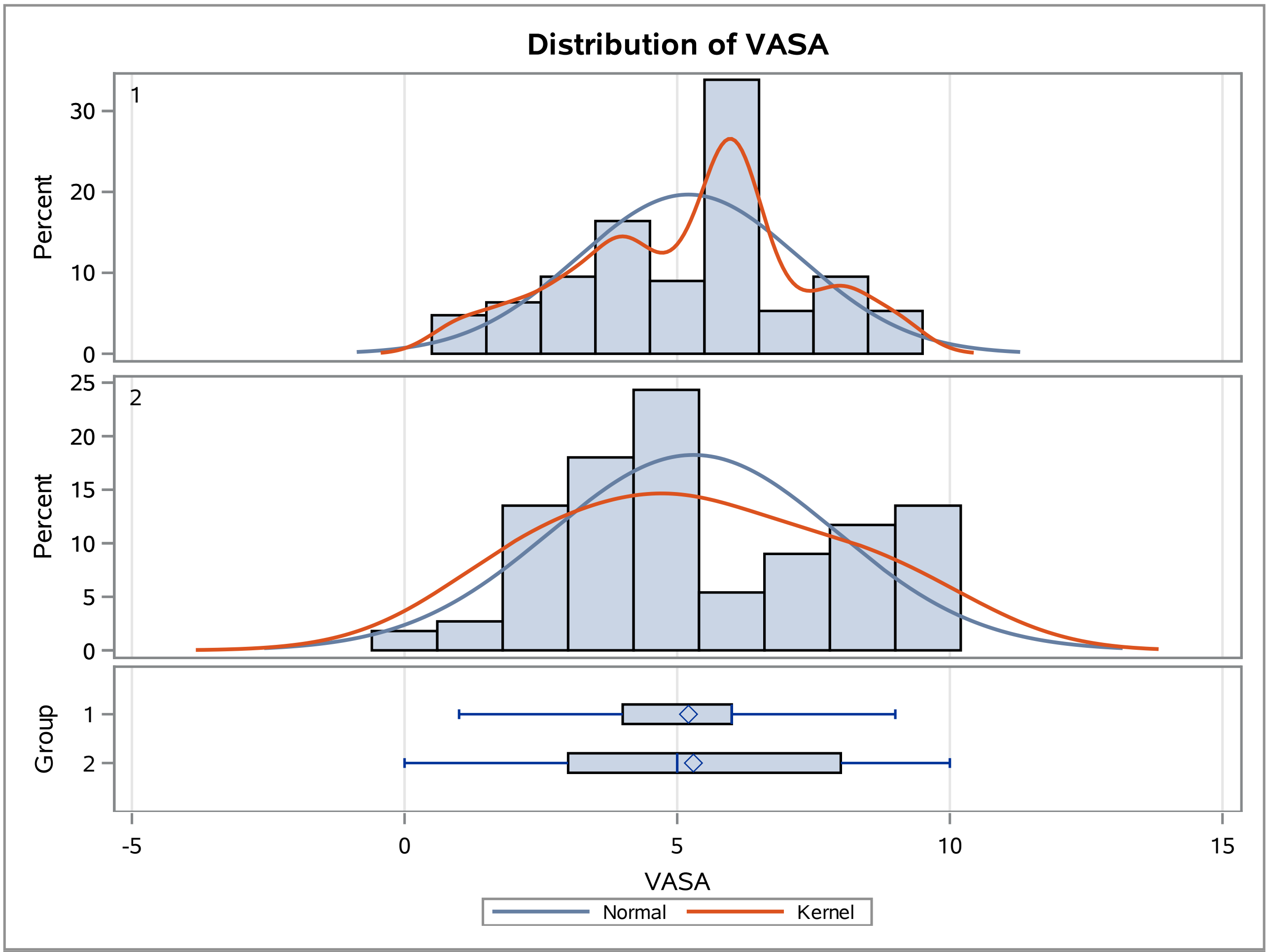

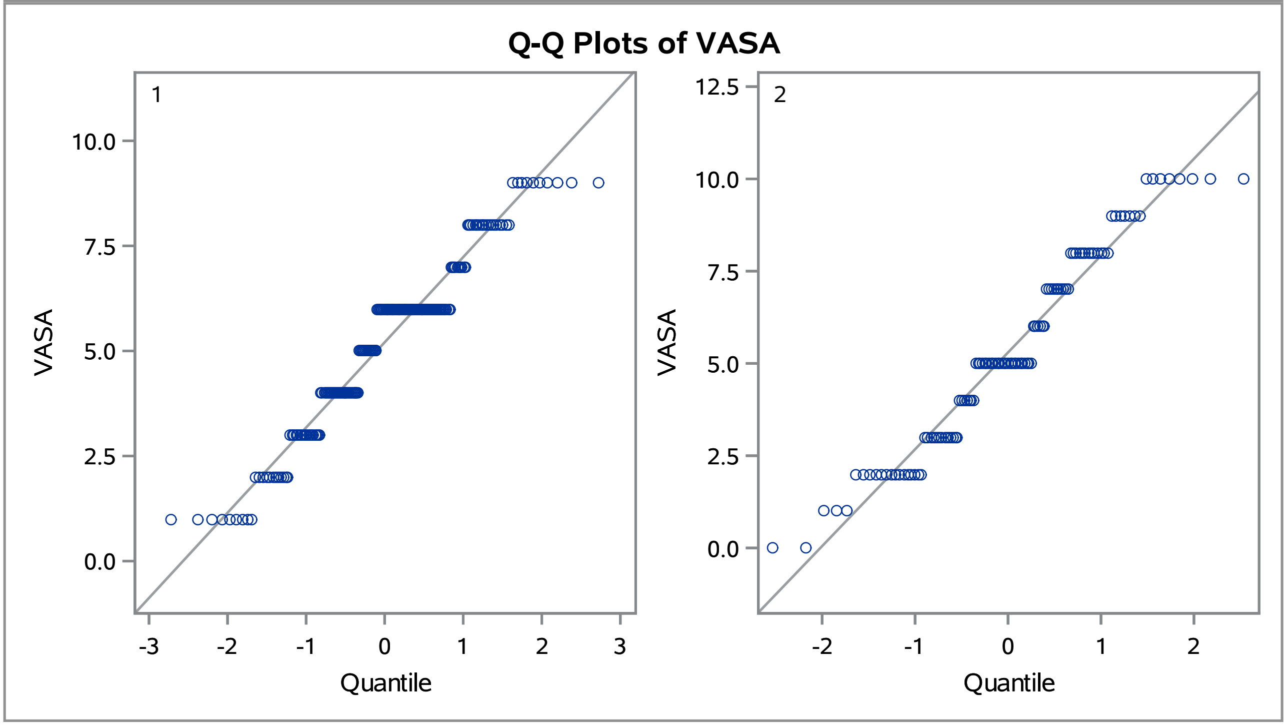

Variable: SAS
Group = 2

Variable: SAS

Variable: SAS

Group	N	Mean	Std Dev	Std Err	Minimum	Maximum
1	189	36.2328	7.3375	0.5337	23.0000	70.0000
2	111	37.0541	7.3631	0.6989	21.0000	56.0000
Diff (1-2)		-0.8212	7.3470	0.8786		

Group	Method	Mean	95% CL Mean		Std Dev	95% CL Std Dev	
1		36.2328	35.1799	37.2857	7.3375	6.6649	8.1624
2		37.0541	35.6690	38.4391	7.3631	6.5054	8.4833
Diff (1-2)	Pooled	-0.8212	-2.5502	0.9077	7.3470	6.8015	7.9883
Diff (1-2)	Satterthwaite	-0.8212	-2.5539	0.9114			

Method	Variances	DF	t Value	Pr > |t|
Pooled	Equal	298	-0.93	0.3507
Satterthwaite	Unequal	229.96	-0.93	0.3513

Equality of Variances				
Method	Num DF	Den DF	F Value	Pr > F
Folded F	110	188	1.01	0.9555

Variable: SAS
Group = 2

Variable: SAS

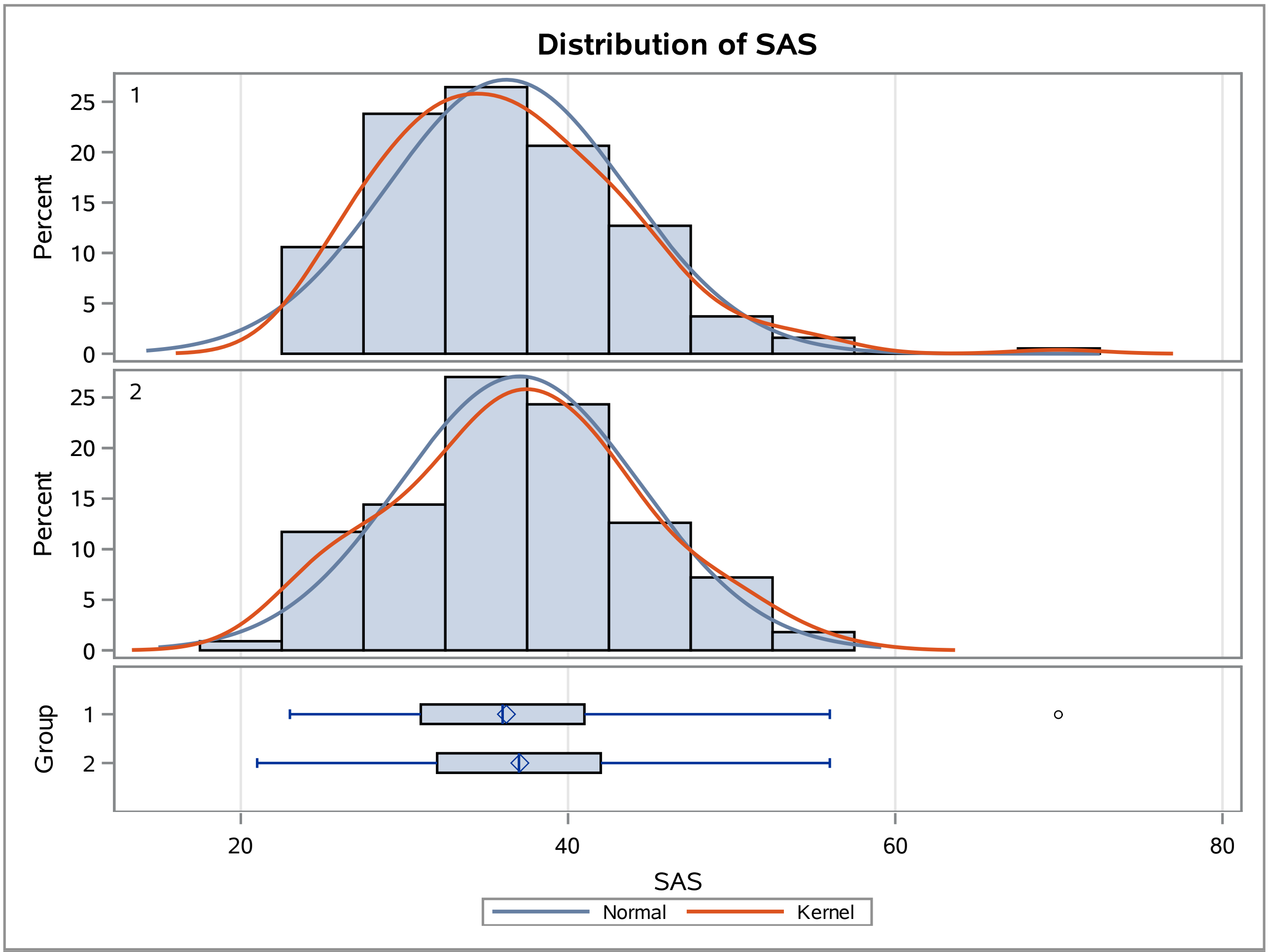

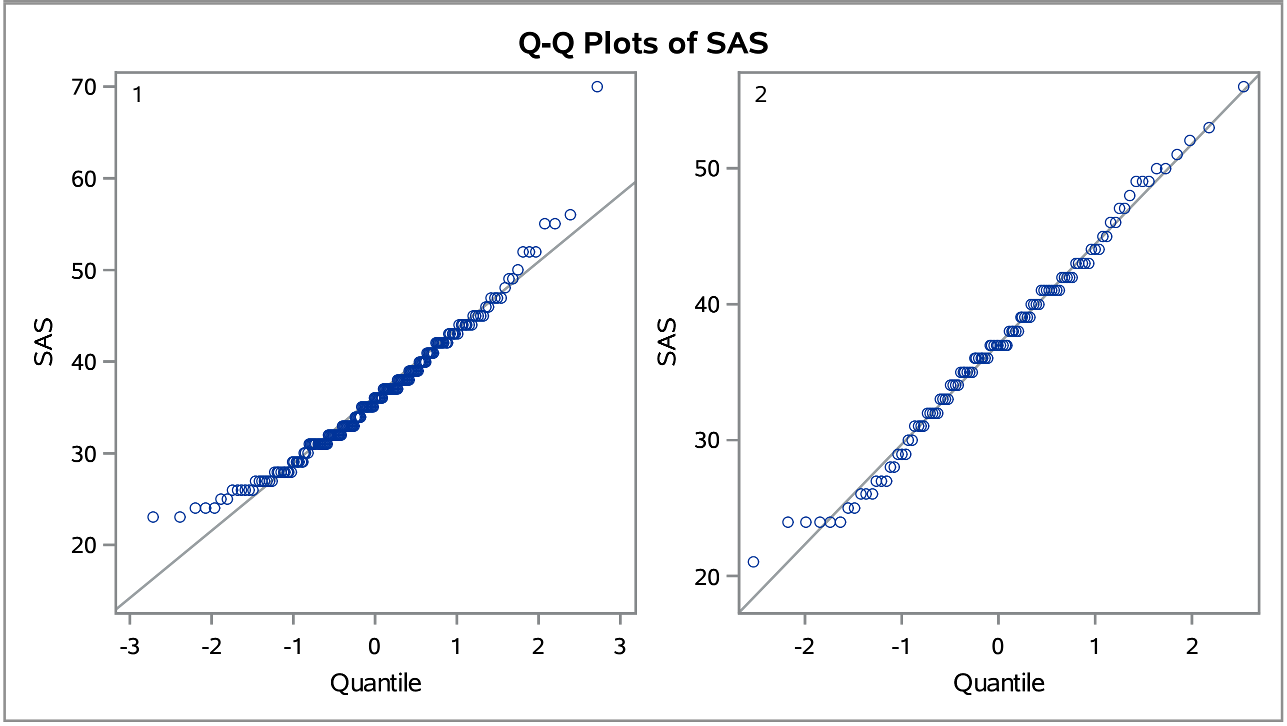

Variable: ISI
Group = 2

Variable: ISI

Variable: ISI

Group	N	Mean	Std Dev	Std Err	Minimum	Maximum
**1**	189	8.5767	6.2544	0.4549	0	28.0000
**2**	111	10.7027	5.2007	0.4936	2.0000	28.0000
**Diff (1-2)**		-2.1260	5.8875	0.7040		

Group	Method	Mean	95% CL Mean		Std Dev	95% CL Std Dev	
**1**		8.5767	7.6793	9.4742	6.2544	5.6811	6.9575
**2**		10.7027	9.7244	11.6810	5.2007	4.5949	5.9919
**Diff (1-2)**	**Pooled**	-2.1260	-3.5115	-0.7405	5.8875	5.4504	6.4014
**Diff (1-2)**	**Satterthwaite**	-2.1260	-3.4477	-0.8042			

Method	Variances	DF	t Value	Pr > |t|
**Pooled**	Equal	298	-3.02	0.0027
**Satterthwaite**	Unequal	264.55	-3.17	0.0017

Equality of Variances				
**Method**	**Num DF**	**Den DF**	**F Value**	**Pr > F**
**Folded F**	188	110	1.45	0.0344

Variable: ISI
Group = 2

Variable: ISI

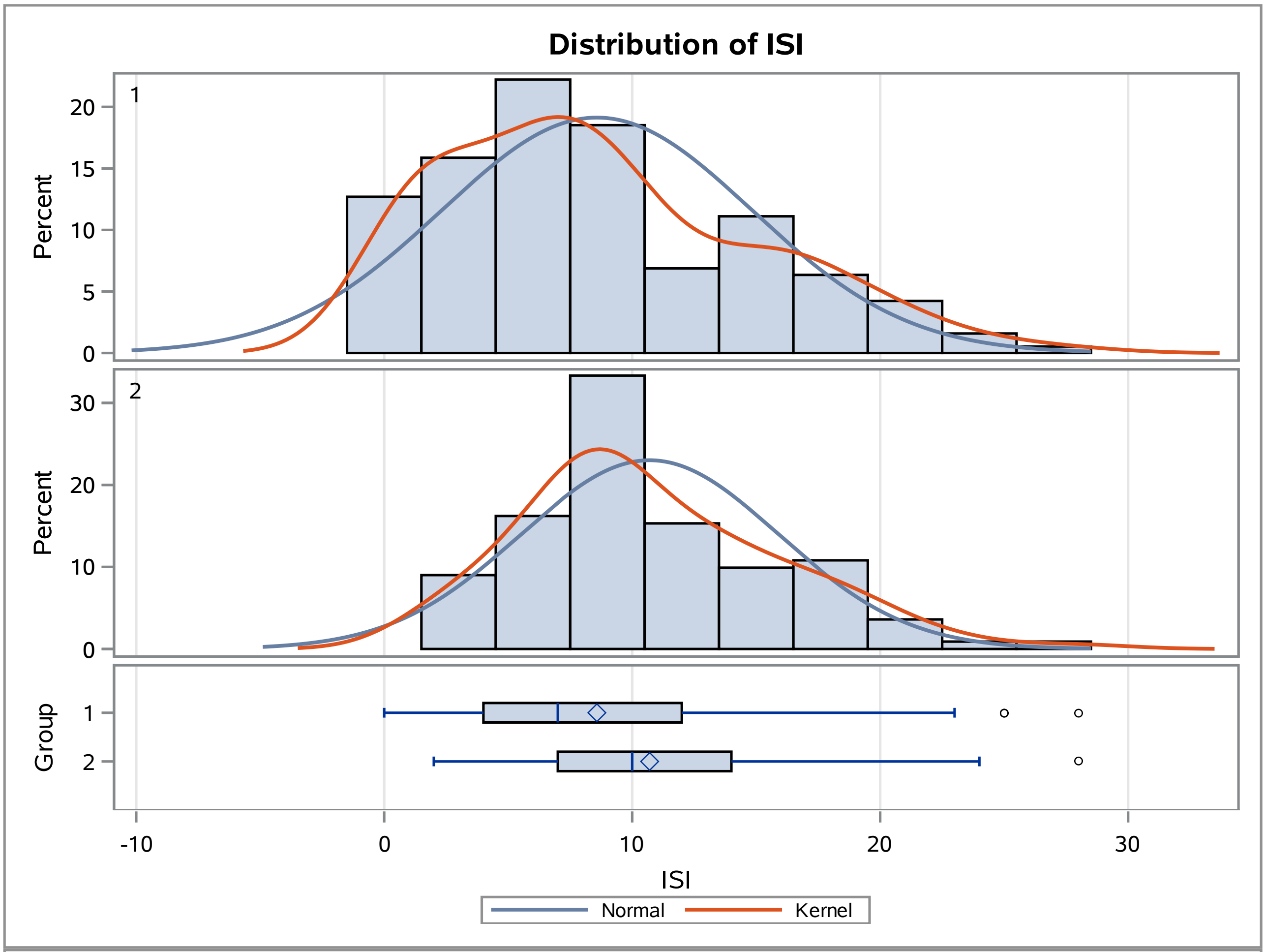

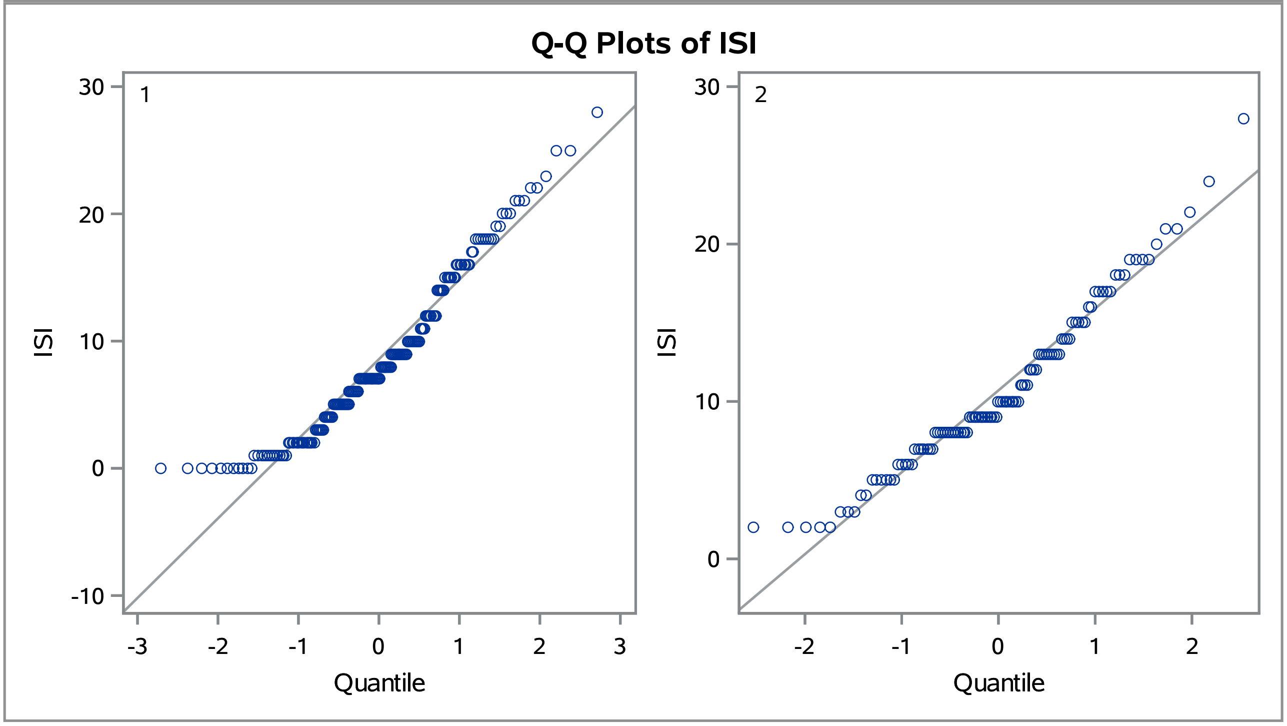

Variable: FCV
Group = 2

Variable: FCV

Variable: FCV

Group	N	Mean	Std Dev	Std Err	Minimum	Maximum
**1**	189	17.4815	5.9444	0.4324	7.0000	34.0000
**2**	111	17.4324	5.3557	0.5083	7.0000	35.0000
**Diff (1-2)**		0.0490	5.7342	0.6857		

Group	Method	Mean	95% CL Mean		Std Dev	95% CL Std Dev	
**1**		17.4815	16.6285	18.3344	5.9444	5.3995	6.6127
**2**		17.4324	16.4250	18.4399	5.3557	4.7319	6.1706
**Diff (1-2)**	**Pooled**	0.0490	-1.3004	1.3985	5.7342	5.3084	6.2347
**Diff (1-2)**	**Satterthwaite**	0.0490	-1.2653	1.3634			

Method	Variances	DF	t Value	Pr > |t|
**Pooled**	Equal	298	0.07	0.9430
**Satterthwaite**	Unequal	250.14	0.07	0.9415

Equality of Variances				
**Method**	**Num DF**	**Den DF**	**F Value**	**Pr > F**
**Folded F**	188	110	1.23	0.2303

Variable: FCV
Group = 2

Variable: FCV

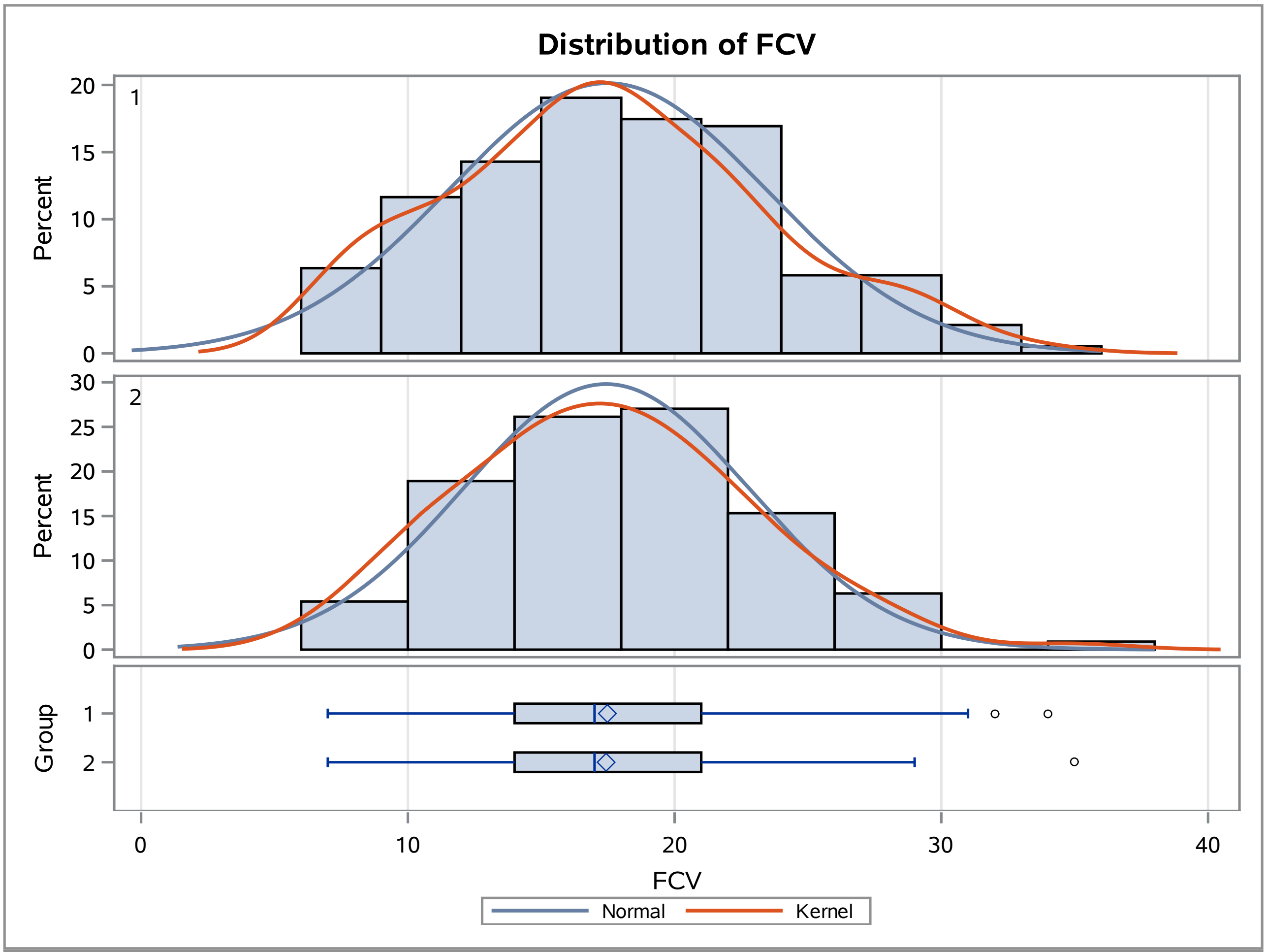

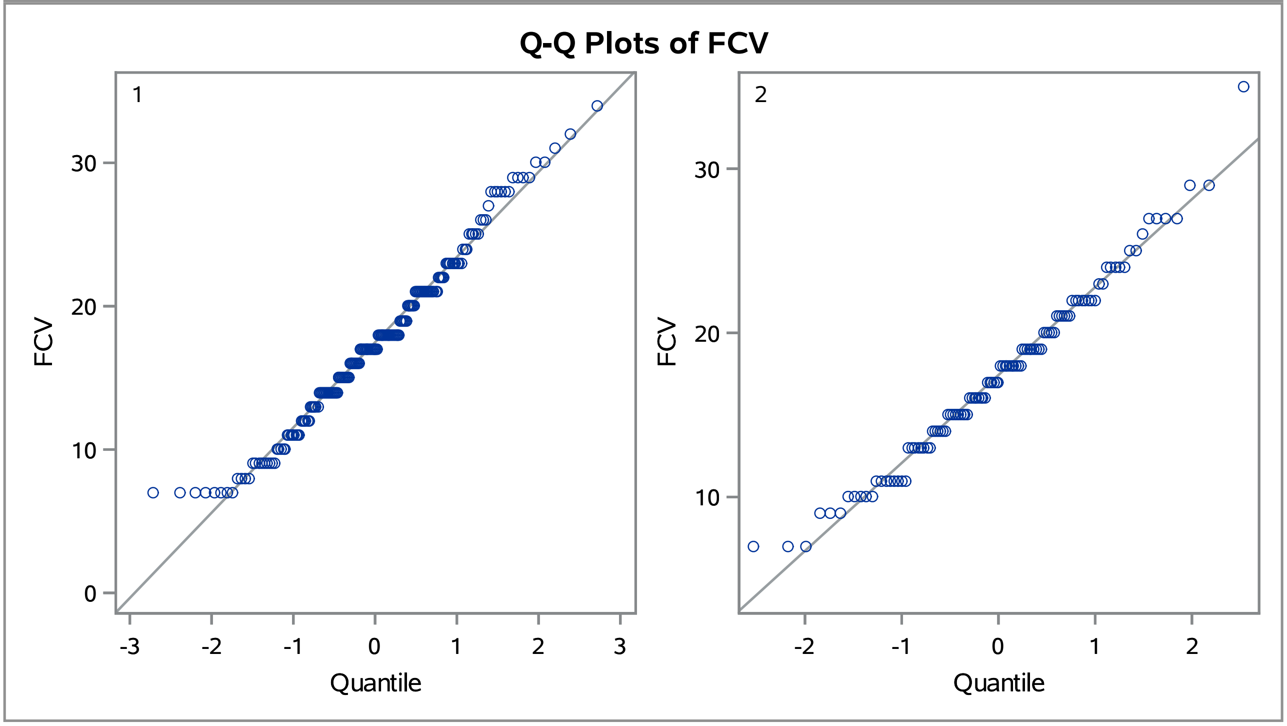

Variable: WHOQOL
Group = 2

Variable: WHOQOL

Variable: WHOQOL

Group	N	Mean	Std Dev	Std Err	Minimum	Maximum
1	189	84.0000	13.9970	1.0181	49.0000	118.0
2	111	89.0721	15.2528	1.4477	54.0000	123.0
Diff (1-2)		-5.0721	14.4732	1.7307		

Group	Method	Mean	95% CL Mean		Std Dev	95% CL Std Dev	
1		84.0000	81.9916	86.0084	13.9970	12.7138	15.5704
2		89.0721	86.2030	91.9411	15.2528	13.4762	17.5734
Diff (1-2)	Pooled	-5.0721	-8.4781	-1.6660	14.4732	13.3987	15.7366
Diff (1-2)	Satterthwaite	-5.0721	-8.5606	-1.5835			

Method	Variances	DF	t Value	Pr > |t|
Pooled	Equal	298	-2.93	0.0036
Satterthwaite	Unequal	214.95	-2.87	0.0046

Equality of Variances				
Method	Num DF	Den DF	F Value	Pr > F
Folded F	110	188	1.19	0.3019

Variable: WHOQOL
Group = 2

Variable: WHOQOL

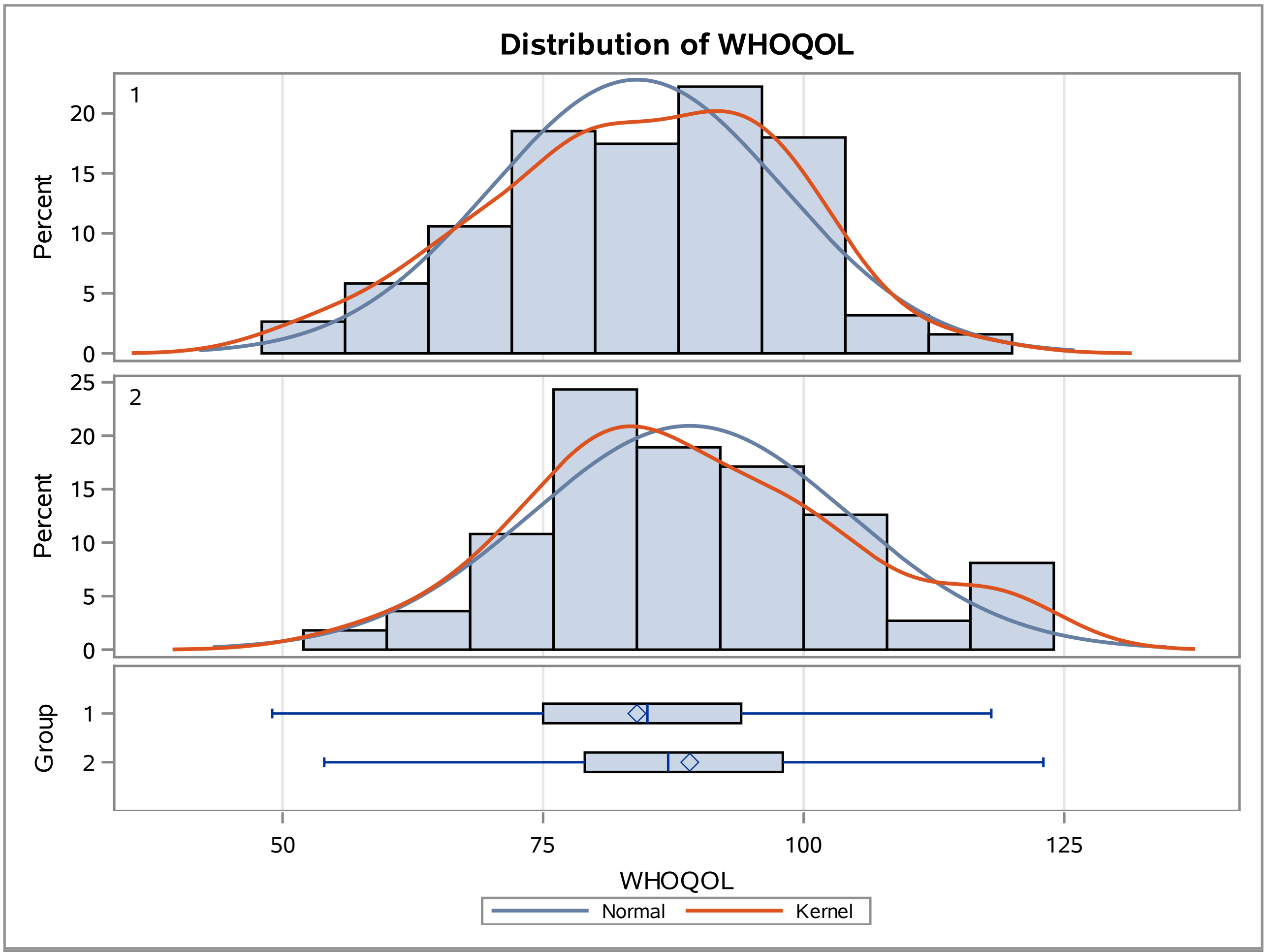

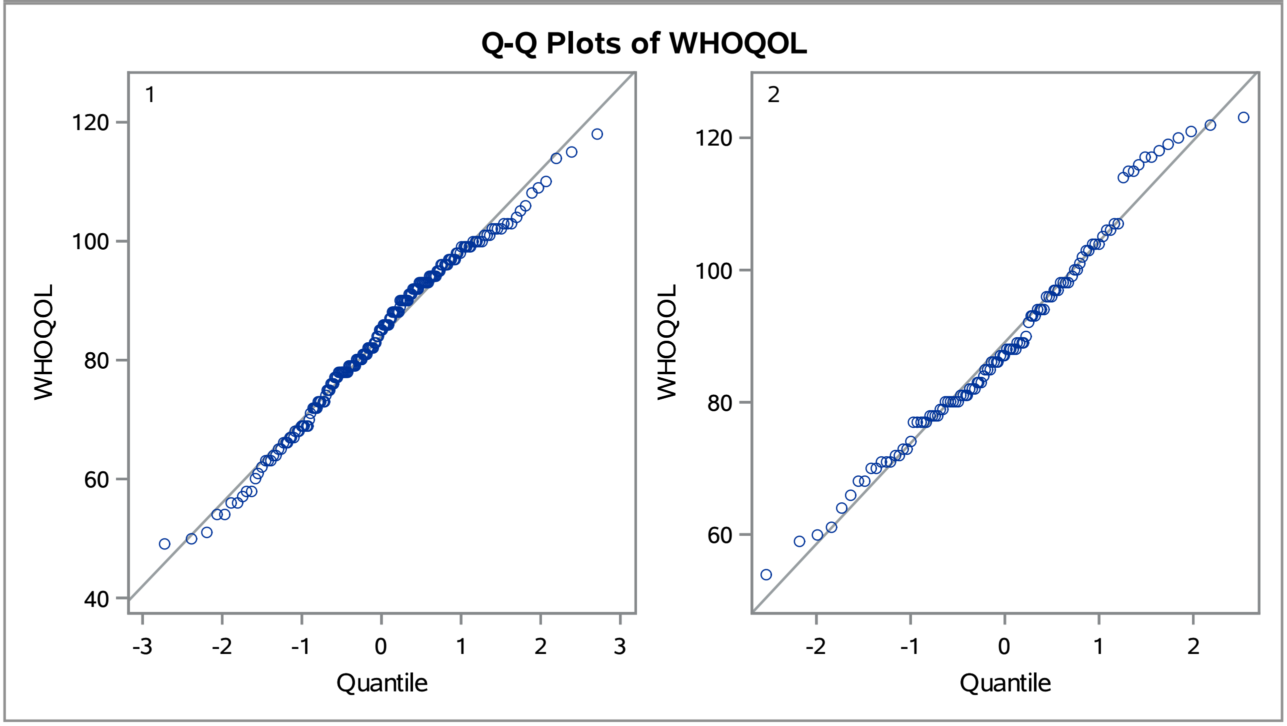

Variable: PRMQ
Group = 2

Variable: PRMQ

Variable: PRMQ

Group	N	Mean	Std Dev	Std Err	Minimum	Maximum
1	189	30.1693	8.6801	0.6314	16.0000	58.0000
2	111	32.6036	9.4852	0.9003	17.0000	60.0000
Diff (1-2)		-2.4343	8.9857	1.0745		

Group	Method	Mean	95% CL Mean		Std Dev	95% CL Std Dev	
1		30.1693	28.9238	31.4148	8.6801	7.8843	9.6558
2		32.6036	30.8194	34.3878	9.4852	8.3803	10.9283
Diff (1-2)	Pooled	-2.4343	-4.5489	-0.3197	8.9857	8.3185	9.7700
Diff (1-2)	Satterthwaite	-2.4343	-4.6017	-0.2668			

Method	Variances	DF	t Value	Pr > |t|
Pooled	Equal	298	-2.27	0.0242
Satterthwaite	Unequal	214.46	-2.21	0.0279

Equality of Variances				
Method	Num DF	Den DF	F Value	Pr > F
Folded F	110	188	1.19	0.2867

Variable: PRMQ
Group = 2

Variable: PRMQ

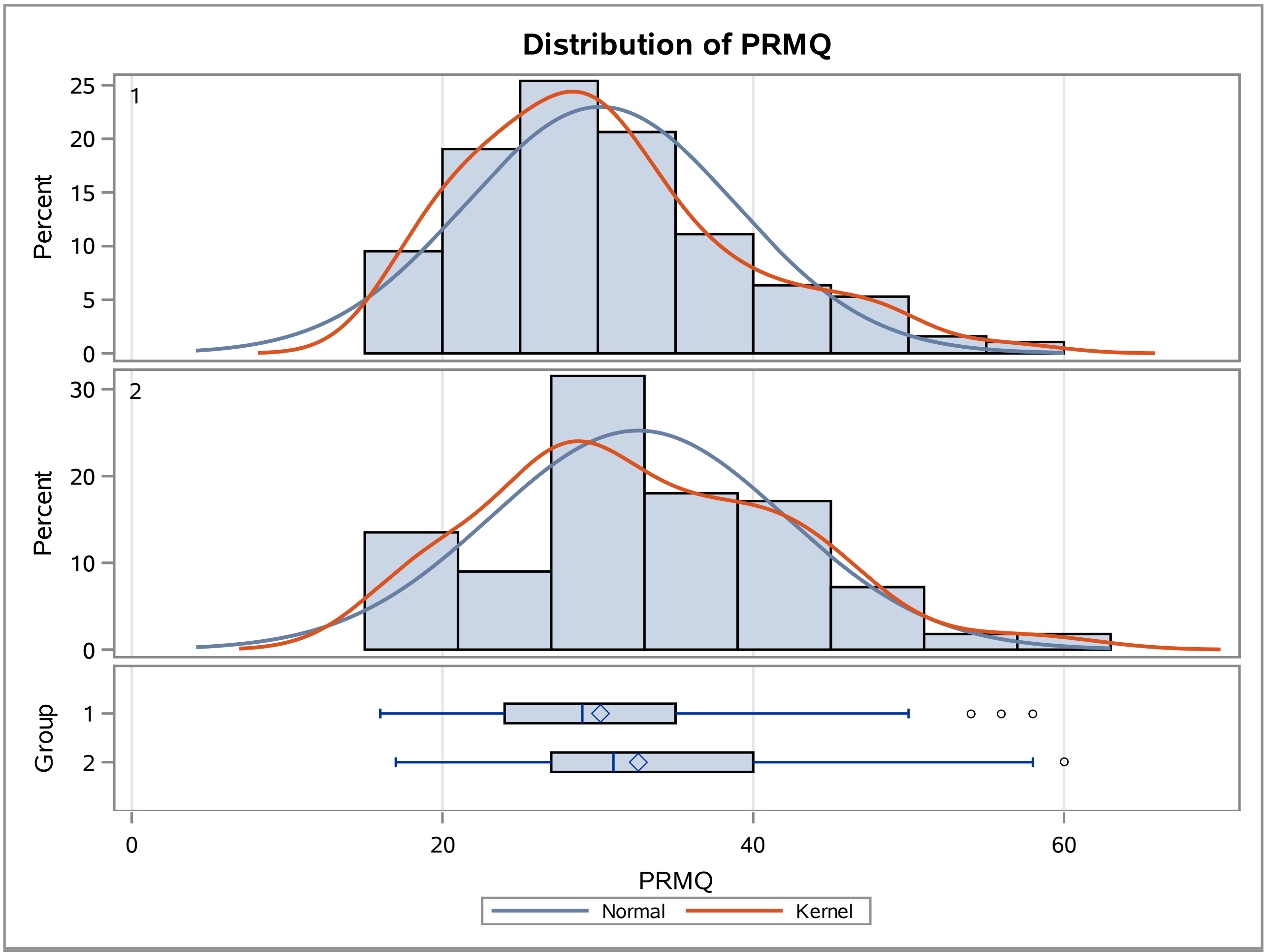

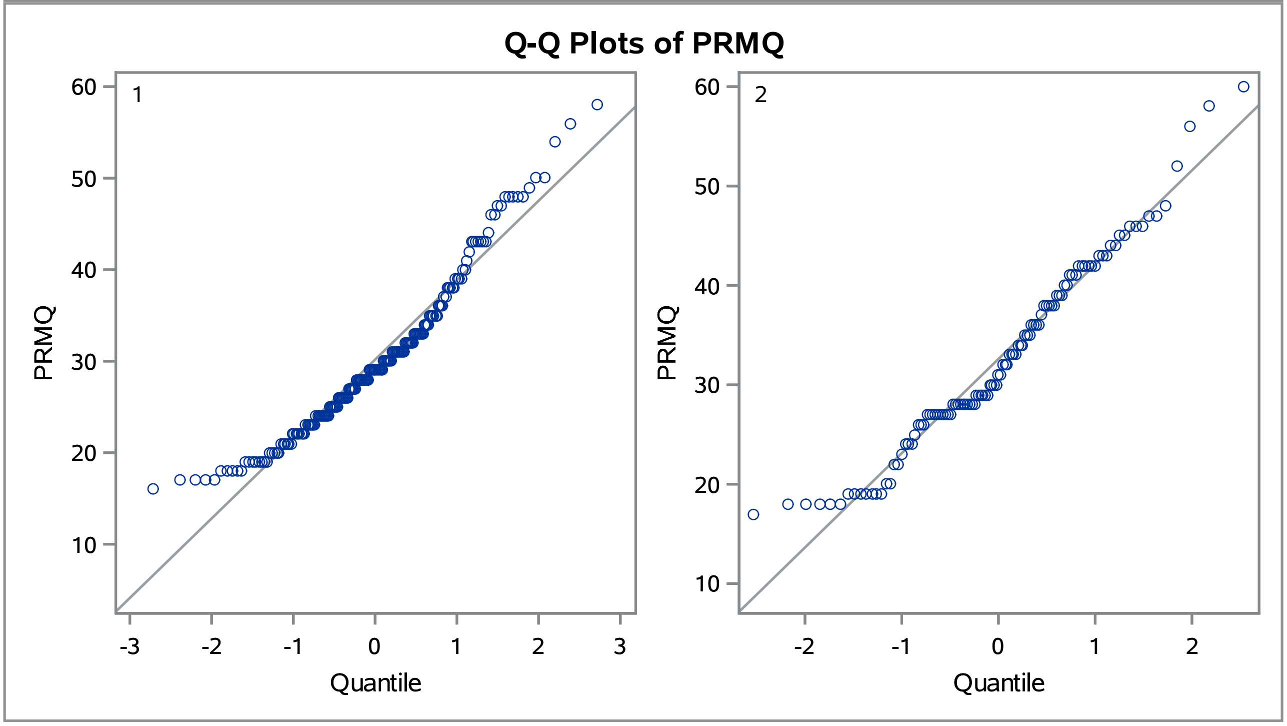

Variable: PM
Group = 2

Variable: PM

Variable: PM

Group	N	Mean	Std Dev	Std Err	Minimum	Maximum
1	189	15.4709	4.3486	0.3163	8.0000	28.0000
2	111	18.4775	5.6470	0.5360	9.0000	31.0000
Diff (1-2)		-3.0066	4.8683	0.5822		

Group	Method	Mean	95% CL Mean		Std Dev	95% CL Std Dev	
1		15.4709	14.8469	16.0949	4.3486	3.9499	4.8374
2		18.4775	17.4153	19.5397	5.6470	4.9892	6.5061
Diff (1-2)	Pooled	-3.0066	-4.1523	-1.8609	4.8683	4.5069	5.2933
Diff (1-2)	Satterthwaite	-3.0066	-4.2343	-1.7788			

Method	Variances	DF	t Value	Pr > |t|
Pooled	Equal	298	-5.16	<.0001
Satterthwaite	Unequal	186.71	-4.83	<.0001

Equality of Variances				
Method	Num DF	Den DF	F Value	Pr > F
Folded F	110	188	1.69	0.0017

Variable: PM
Group = 2

Variable: PM

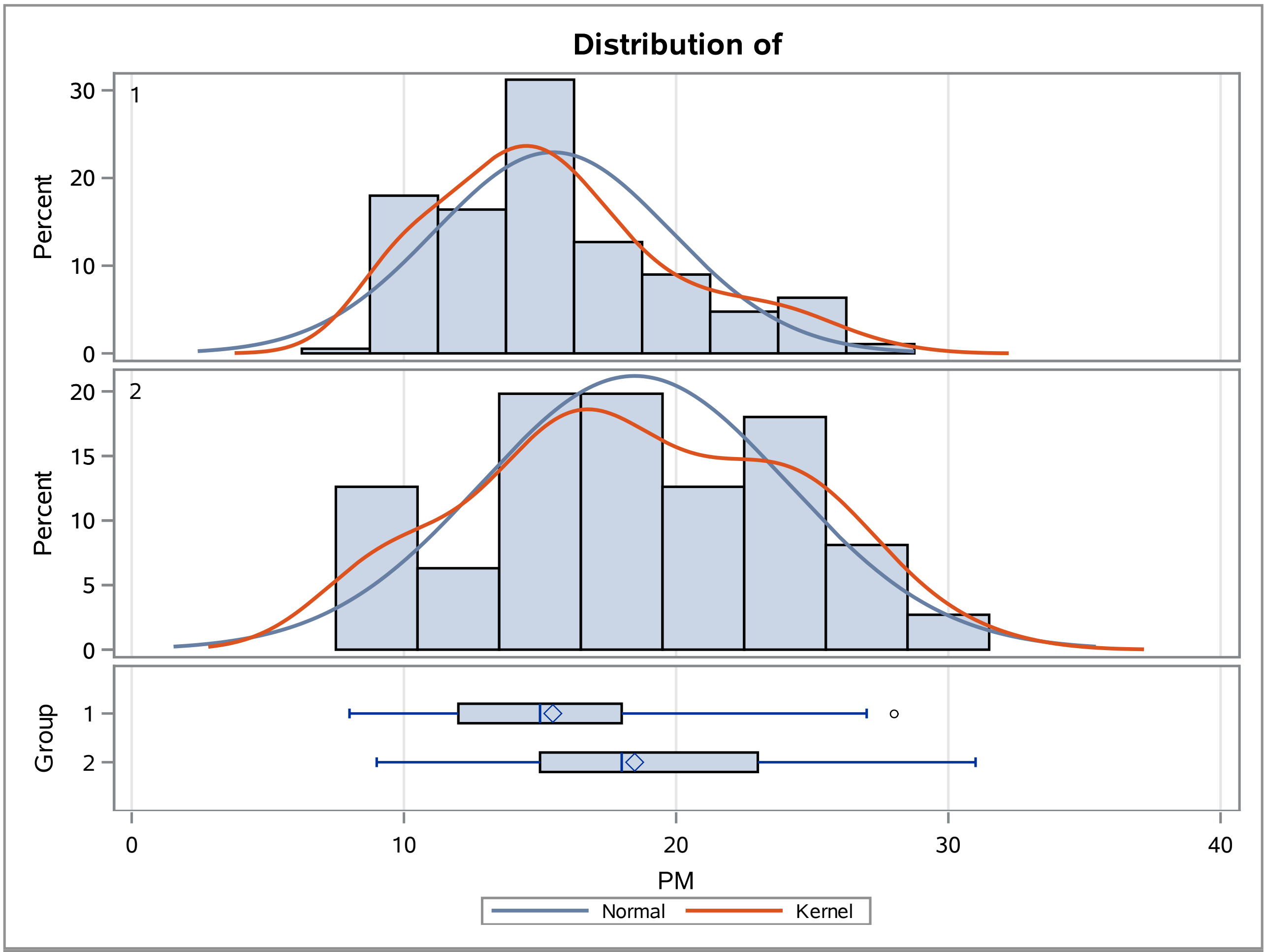

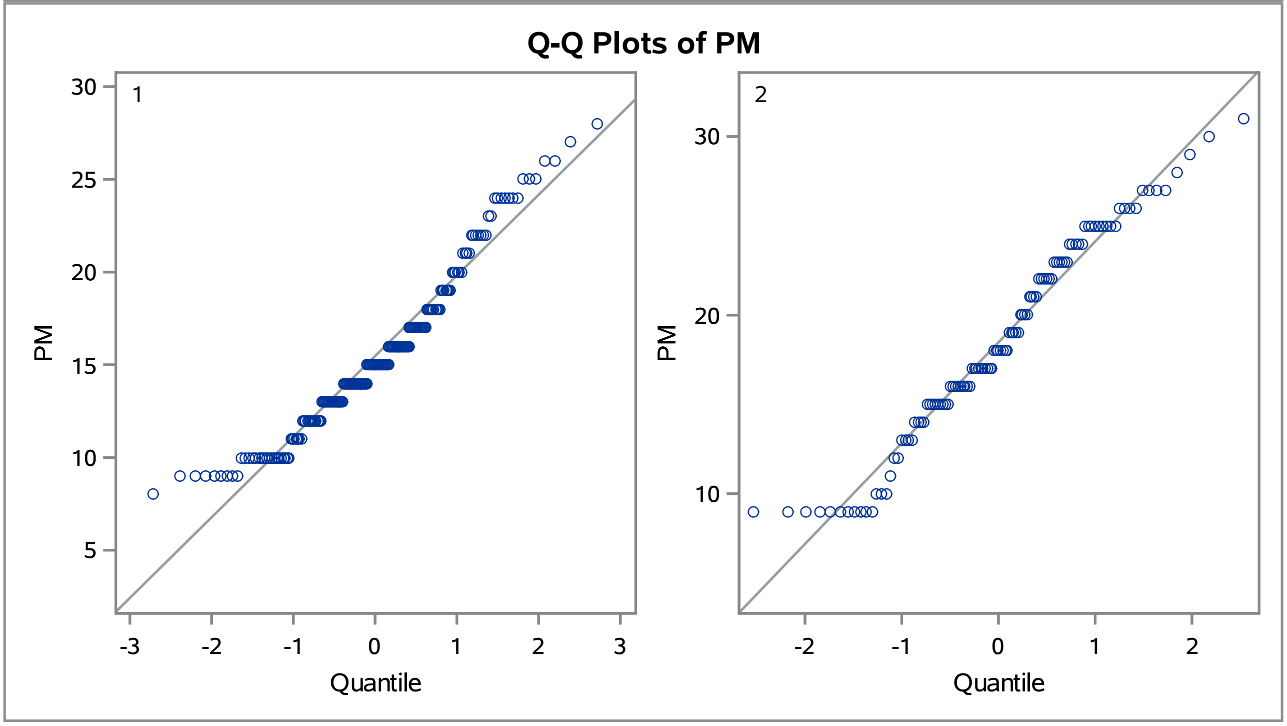

Variable: RM
Group = 2

Variable: RM

Variable: RM

Group	N	Mean	Std Dev	Std Err	Minimum	Maximum
1	189	14.6984	4.6102	0.3353	8.0000	30.0000
2	111	14.1261	4.4927	0.4264	8.0000	32.0000
Diff (1-2)		0.5723	4.5672	0.5462		

Group	Method	Mean	95% CL Mean		Std Dev	95% CL Std Dev	
1		14.6984	14.0369	15.3599	4.6102	4.1876	5.1285
2		14.1261	13.2811	14.9712	4.4927	3.9693	5.1762
Diff (1-2)	Pooled	0.5723	-0.5025	1.6471	4.5672	4.2281	4.9659
Diff (1-2)	Satterthwaite	0.5723	-0.4965	1.6410			

Method	Variances	DF	t Value	Pr > |t|
Pooled	Equal	298	1.05	0.2956
Satterthwaite	Unequal	235.44	1.05	0.2925

Equality of Variances				
Method	Num DF	Den DF	F Value	Pr > F
Folded F	188	110	1.05	0.7731

Variable: RM
Group = 2

Variable: RM

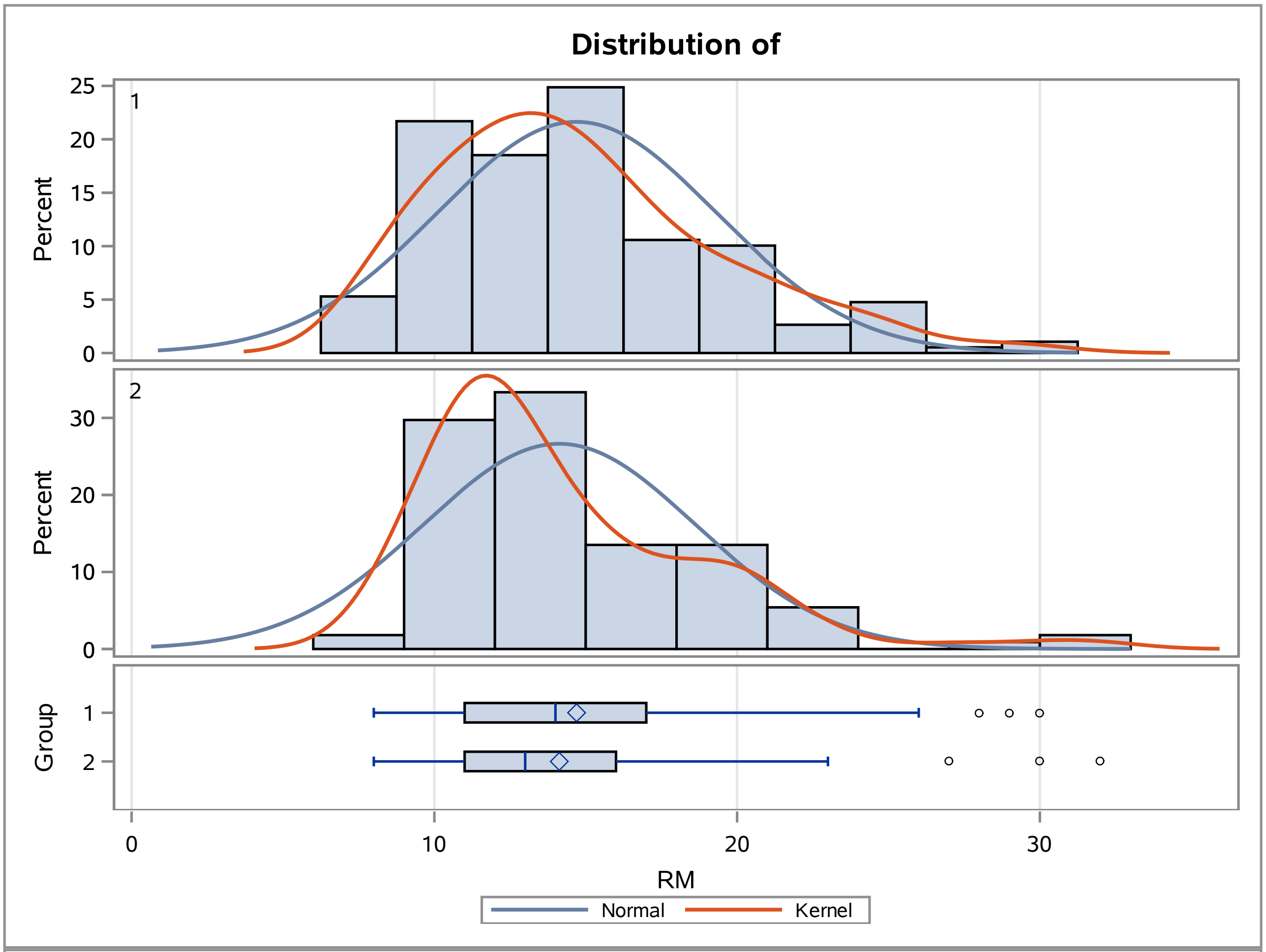

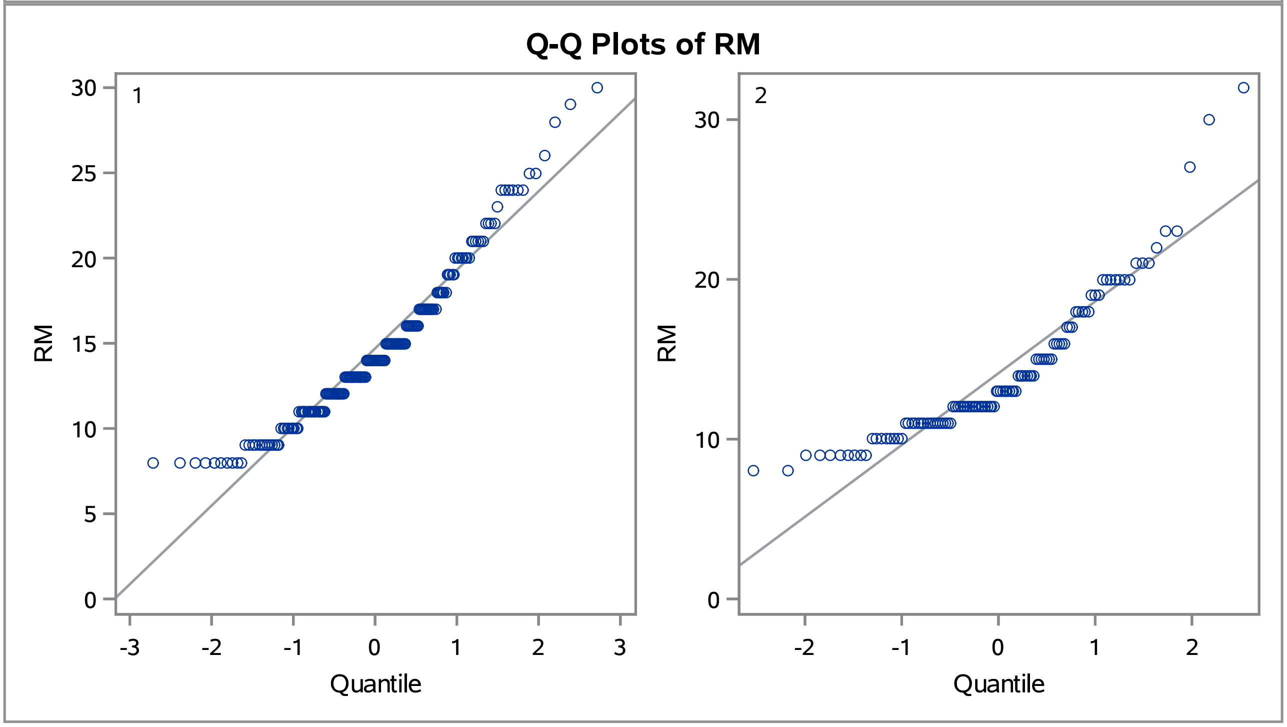

Variable: SMCQ
Group = 2

Variable: SMCQ

Variable: SMCQ

Group	N	Mean	Std Dev	Std Err	Minimum	Maximum
1	189	2.6402	2.5511	0.1856	0	14.0000
2	111	2.9640	2.6489	0.2514	0	10.0000
Diff (1-2)		-0.3238	2.5877	0.3094		

Group	Method	Mean	95% CL Mean		Std Dev	95% CL Std Dev	
1		2.6402	2.2742	3.0063	2.5511	2.3172	2.8379
2		2.9640	2.4657	3.4622	2.6489	2.3404	3.0519
Diff (1-2)	Pooled	-0.3238	-0.9327	0.2852	2.5877	2.3955	2.8135
Diff (1-2)	Satterthwaite	-0.3238	-0.9396	0.2920			

Method	Variances	DF	t Value	Pr > |t|
Pooled	Equal	298	-1.05	0.2963
Satterthwaite	Unequal	223.65	-1.04	0.3013

Equality of Variances				
Method	Num DF	Den DF	F Value	Pr > F
Folded F	110	188	1.08	0.6464

Variable: SMCQ
Group = 2

Variable: SMCQ

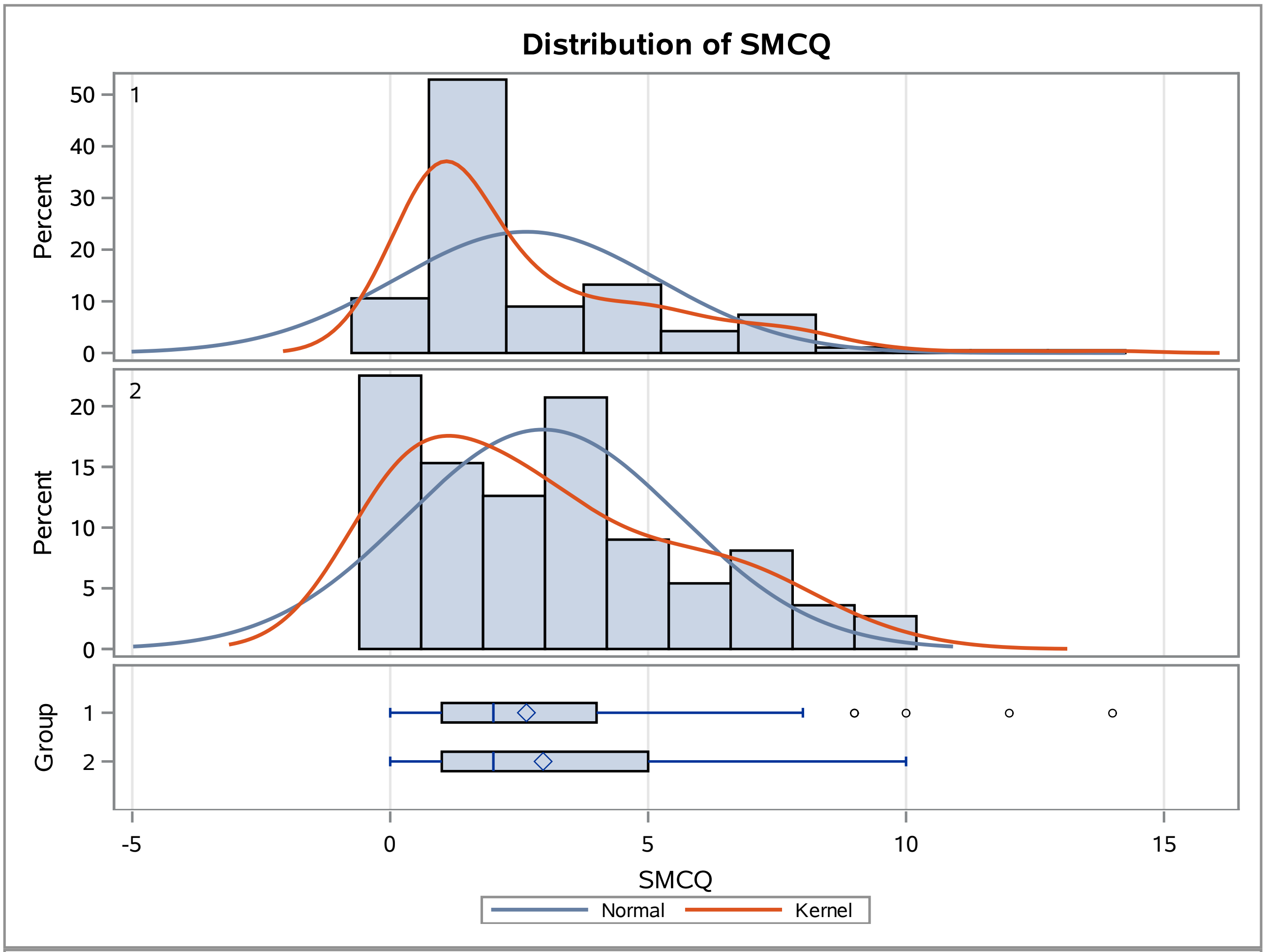

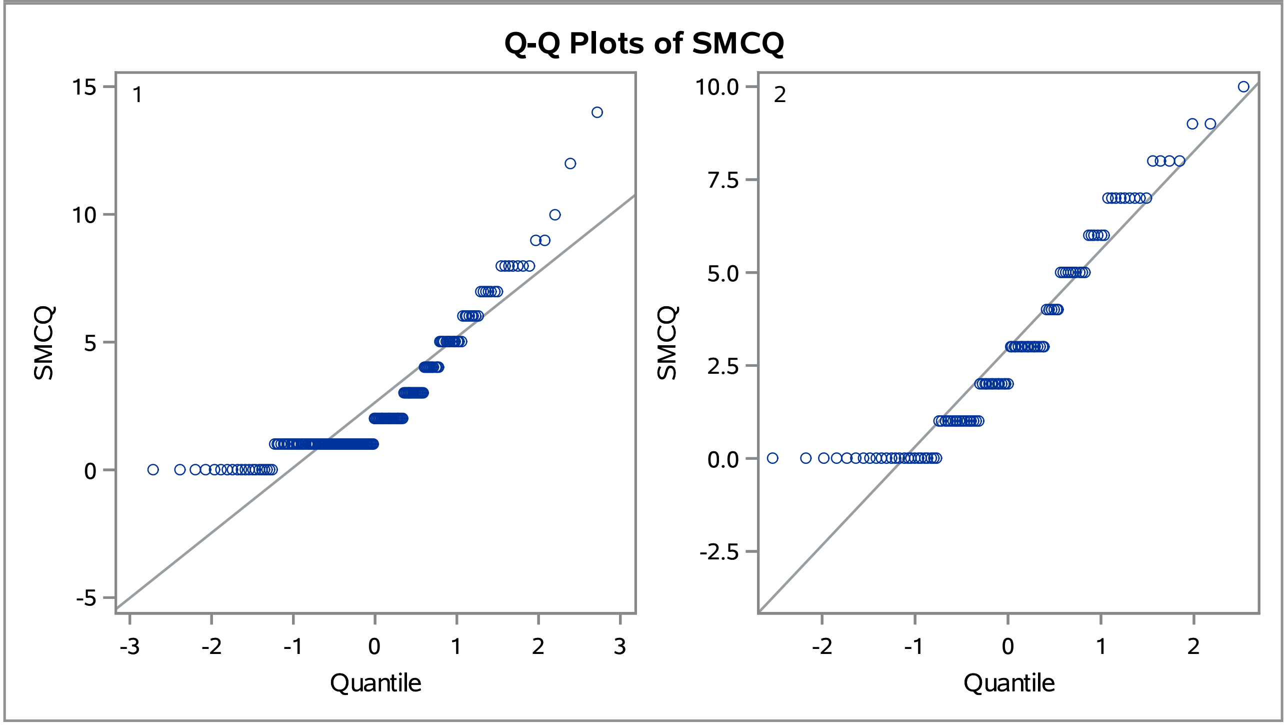

Supplement: Supplementary file 2 — Supplementary Material 2 [file 12877_2023_4646_MOESM2_ESM.pdf]
